# Multiple mechanisms of MYCN dysregulation in Wilms tumour

**DOI:** 10.18632/oncotarget.3377

**Published:** 2015-01-31

**Authors:** Richard D. Williams, Tasnim Chagtai, Marisa Alcaide-German, John Apps, Jenny Wegert, Sergey Popov, Gordan Vujanic, Harm van Tinteren, Marry M. van den Heuvel-Eibrink, Marcel Kool, Jan de Kraker, David Gisselsson, Norbert Graf, Manfred Gessler, Kathy Pritchard-Jones

**Affiliations:** ^1^ UCL Institute of Child Health, London, UK; ^2^ Theodor-Boveri-Institute/Biocenter, Developmental Biochemistry and Comprehensive Cancer Center Mainfranken, Wuerzburg University, Wuerzburg, Germany; ^3^ Institute of Cancer Research, Sutton, Surrey, UK; ^4^ Cardiff University School of Medicine, Heath Park, Cardiff, UK; ^5^ Biometrics Department, Netherlands Cancer Institute, Antonie van Leeuwenhoek Ziekenhuis, Amsterdam, The Netherlands; ^6^ Department of Pediatric Oncology/Hematology, Erasmus MC, Sophia Children's Hospital, Rotterdam, The Netherlands; ^7^ German Cancer Research Centre, Heidelberg, Germany; ^8^ Academic Medical Center, Amsterdam, The Netherlands; ^9^ Department of Clinical Genetics, Lund University, Sweden; ^10^ Department of Paediatric Oncology and Haematology, Saarland University Hospital, Homburg/Saar, Germany

**Keywords:** Wilms tumour, MYCN, copy number, DNA methylation, prognostic marker

## Abstract

Genomic gain of the proto-oncogene transcription factor gene *MYCN* is associated with poor prognosis in several childhood cancers. Here we present a comprehensive copy number analysis of *MYCN* in Wilms tumour (WT), demonstrating that gain of this gene is associated with anaplasia and with poorer relapse-free and overall survival, independent of histology. Using whole exome and gene-specific sequencing, together with methylation and expression profiling, we show that *MYCN* is targeted by other mechanisms, including a recurrent somatic mutation, P44L, and specific DNA hypomethylation events associated with *MYCN* overexpression in tumours with high risk histologies. We describe parallel evolution of genomic copy number gain and point mutation of *MYCN* in the contralateral tumours of a remarkable bilateral case in which independent contralateral mutations of *TP53* also evolve over time. We report a second bilateral case in which *MYCN* gain is a germline aberration. Our results suggest a significant role for *MYCN* dysregulation in the molecular biology of Wilms tumour. We conclude that *MYCN* gain is prognostically significant, and suggest that the novel P44L somatic variant is likely to be an activating mutation.

## INTRODUCTION

Wilms tumour (WT, nephroblastoma) is the commonest paediatric renal malignancy. A significant minority of WT cases have mutations in known genes, including *WT1* [1-6], *CTNNB1* [7, 8], *AMER1* (*WTX*) [9], and *TP53* [10]. Less frequent mutations targeting genes such as *FBXW7* [11] and *GPC3* [12] have also been reported, and epigenetic lesions affecting the *IGF2*/*H19* locus are common [13]. A recent whole exome study has indentified mutations in microRNA processing genes including *DROSHA*, *DGCR8* and *DICER1* [14]. Numerous recurrent copy number aberrations and loss of heterozygosity (LOH) events have been described, some of which affect known genes (e.g. 11p LOH, 17p loss, focal deletion of *AMER1* and *FBXW7*), while the functional significance of others (e.g. 1q gain, 1p loss, 16q loss) is unclear. Only a few of these aberrations have known associations with histology or outcome. *TP53* mutations are largely confined to (high risk) anaplastic tumours [10], while *WT1* mutations (which are frequently coincident with *CTNNB1* mutations) are associated with stromal predominant histology [15]. We have previously noted an association between 1q gain and relapse [16, 17], but the only genomic biomarker with a fully validated association with poor outcome that is currently used in treatment planning is simultaneous loss of heterozygosity of 1p and 16q [18].

One recurrent focal copy number gain on 2p24.3, encompassing the *MYCN* locus, has been observed in several previous WT studies [11, 19-23]. The *MYCN* gene encodes a proto-oncogenic MYC family transcription factor, MYCN. *MYCN* is known to be amplified in 16-25% of neuroblastomas, an aberration associated with a poorer prognosis, and it undergoes prognostically relevant copy number gain, and more rarely amplification, in other paediatric tumours including medulloblastoma [24, 25] and rhabdomyosarcoma [26]. In an earlier study [11], we used SNP arrays to analyse tumours from patients who had received pre-operative chemotherapy under International Society of Paediatric Oncology (SIOP) protocols, and noted an apparent association between *MYCN* gain and the high risk diffuse anaplastic subtype. In a larger study [22], in which genomic real-time PCR was used to measure *MYCN* copy number in a more heterogenous group of tumours from patients treated under various protocols (including immediate nephrectomy), we confirmed that *MYCN* gain is a common event, but found no particular subtype association. In the current report, we describe the largest analysis of *MYCN* status in WT to date, using multiplex ligation dependent probe amplification (MLPA) to assess its copy number in a series of samples drawn entirely from the SIOP WT 2001 clinical study and trial.

Recent developments in neuroblastoma genomics suggest that gain or amplification is not the only mechanism that affects MYCN function in paediatric tumours. Maris and co-workers, using whole genome sequencing, have shown that a specific somatic variant, P44L, can be detected in 2% of all neuroblastomas analysed [27], while isolated cases of the same variant have also been described in glioma, medulloblastoma, endometrial carcinoma, and neoplastic cysts of the pancreas [28-31]. This variant is postulated to be an activating ‘gain of function' mutation that, like increased MYCN dosage due to copy number gain, could cause oncogenic upregulation of downstream MYCN-dependent pathways.

Here we report the discovery of *MYCN* P44L mutations through whole exome analysis of Wilms tumour, leading us to hypothesise that multiple routes to MYCN-oncogenesis may exist in this paediatric tumour. We present targeted *MYCN* sequencing analysis of a large WT series to determine the frequency of these mutations, and investigate potential correlations between *MYCN* expression levels, copy number events, and gene-specific hypomethylation.

## RESULTS

### Point mutations in the *MYCN* coding sequence

In a whole exome analysis of a series of 51 WTs, enriched in cases with high risk histology or poor outcome, heterozygous somatic point mutations in the *MYCN* coding sequence were detected in three tumours. In all three cases, the same variant, a c.131C>T transition substituting proline with leucine at codon 44 (p.P44L), was detected (Table 1). 45 of the 51 cases gave informative exome data at this position, with sufficient depth of coverage for variant calling. Thus, 6.7% of the cases that could be analysed in the exome series carried this mutation. To determine the proportion of WTs harbouring this or other *MYCN* mutations in a larger, unselected cohort (Supplementary Table 1), we sequenced the complete *MYCN* coding region in 168 additional tumour samples. Where a mutation was detected, we sequenced matched normal kidney or blood germline DNA (if available) from the same case. Five additional tumours carrying P44L were identified (3.0%, Table 1) and, as in the exome series, all mutations were heterozygous in the tumour and absent from the germline. The overall frequency of P44L in the extended series of 213 cases was therefore 3.8%. The subset carrying the mutation had no particular distinguishing features. Multiple histological subtypes (as defined by SIOP) and clinical stages were represented (Table 1). Only one of the patients – the complex bilateral case described in Table 2 and below - has so far suffered a recurrence (minimum follow-up 25 months, median follow-up 54.5 months), and none have died.

**Table 1:** MYCN single nucleotide variants in Wilms tumour

ICH ID	Exome	Genomic (Chr2)	CDS	Protein	Germline	ESP5400	1000g	dbSNP 132	SIFT	Poly - Phen2	Histology	Stage	MYCN CN
2560	Y	g.16082317C>T	c.131C>T	p.P44L	Wild type				D	D	Focal Anaplastic	3	Normal
3863	Y	g.16082317C>T	c.131C>T	p.P44L	Wild type				D	D	Regressive	1	Normal
4788		g.16082317C>T	c.131C>T	p.P44L	Wild type				D	D	Regressive	3	Gain
4956		g.16082317C>T	c.131C>T	p.P44L	Wild type				D	D	Regressive	2	Normal
6613		g.16082317C>T	c.131C>T	p.P44L	Wild type				D	D	Mixed	1	Gain
8783	Y	g.16082317C>T	c.131C>T	p.P44L	Wild type				D	D	Blastemal	2	Normal
8819		g.16082317C>T	c.131C>T	p.P44L	Wild type				D	D	Regressive	3	Gain
9750		g.16082317C>T	c.131C>T	p.P44L	Wild type				D	D	Mixed	1	Normal
3136		g.16085677C>T	c.853C>T	p.R285W	Variant				D	D	Stromal	1	Normal
4692		g.16082413G>A	c.227G>A	p.S76N	N/A				T	B	Regressive	3	Normal
7512		g.16082659C>T	c.473C>T	p.A158V	Variant				T	P	Stromal	2	Gain
9431		g.16085917C>G	c.1093C>G	p.P365A	N/A	0.0005	0.0005		T	B	Regressive	3	Normal
8914		g.16082375C>A	c.189C>A	p.P63P	ND				-	-	Mixed	1	Gain
9140		g.16082393G>A	c.207G>A	p.E69E	ND	0.0027	0.0009	rs41264199	-	-	Regressive	1	Normal
9224		g.16082393G>A	c.207G>A	p.E69E	ND	0.0027	0.0009	rs41264199	-	-	Regressive	1	Normal

SIFT/PolyPhen2: D = damaging, T = tolerated, B = benign, P = possibly damaging. 1000g = variant frequency in 1000 Genome Project (Feb 2012). ESP5400 = variant frequency in Exome Sequencing Project (Dec 2011). N/A = material not available. ND = Not done.

Four further non-synonymous heterozygous variants elsewhere in the *MYCN* coding sequence, each affecting a single case, were also found (Table 1). The first of these, c.853C>T (p.R285W), was present in the germline. R285 is a highly conserved amino acid residue in protein domain pfam01056 (‘Myc amino-terminal region' [32]). This mutation was predicted as damaging by both SIFT [33] and PolyPhen2 [34]. The second variant, c.227G>A (p.S76N), was found in a case for which no blood or unambiguous normal kidney tissue was available, so it was not possible to determine if it was a germline variant. S76 is a poorly conserved residue, and the S76N variant was not predicted as damaging by either SIFT or PolyPhen. A third variant, c.473C>T (p.A158V), was located in another poorly conserved region and predicted as tolerated by SIFT, but possibly damaging by PolyPhen, and was confirmed as present in the germline. The fourth variant, c.1093C>G (p.P365A), was also predicted to be a benign change by both algorithms, and is a known rare variant previously observed in the 1000 Genome Project data [35]. Data from unambiguous germline material were not available. No congenital abnormalities or family history of cancer were reported in three of these cases; no data were available for the A158V case. Two further variants (c.189C>A, c.207G>A), one of which was a rare known SNP, were also identified in three samples (Table 1), but they did not alter the amino acid sequence or overlap with splice sites and were not analysed further.

MLPA copy number analysis of all the cases with *MYCN* sequence variants (as below) showed that three of the eight P44L tumours (ICH-4788, 6613 and 8819) also had evidence of *MYCN* gain, while single control probes on 2p (*DYSF*) and 2q (*PAX3*) detected normal copy number, indicating a focal or regional event rather than whole arm or chromosome gain. ICH-7512, the A158V case, had large scale or complete gain of chromosome 2, as did one of the samples with a silent mutation, ICH-8914.

### Germline *MYCN* gain in a bilateral WT case

**Figure 1:**
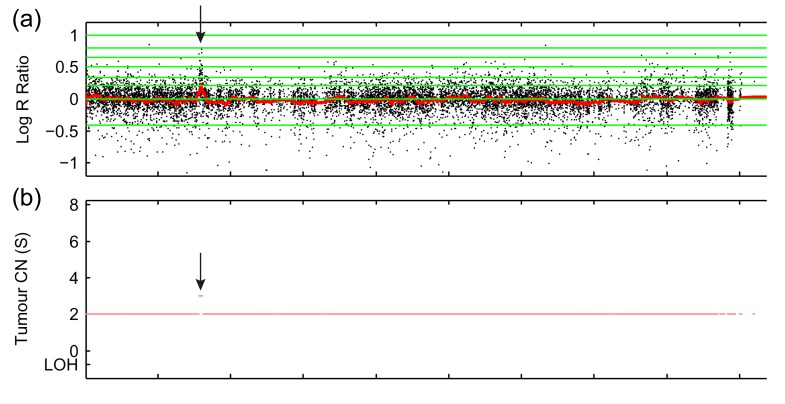
Focal gain of *MYCN* in the germline of a bilateral WT case (a) Illumina CytoSNP-12 raw copy number data (Log R Ratio) from blood sample ICH-10529 showing probes aligned to the 2p region. (b) Copy number segmentation by OncoSNP (linear scale). Arrows: position of region of focal gain. LOH = segmented loss of heterozygosity (none detected).

In a SNP array analysis of a bilateral case, a relatively focal single copy gain of *MYCN* was detected in both contralateral tumours. To determine if this was a germline aberration, or a somatic change (an early event in kidney development or a contralateral metastasis), copy number profiles were also obtained from the (right) normal kidney and the patient's blood DNA. Both samples harboured the region of gain, confirming that this was a germline event. Segmentation of the copy number data with OncoSNP detected a <0.5 Mb region of single copy gain (Figure 1, Supplementary Figure 1), spanning 98 SNP probes from rs2287273 (chr2: 15658523) to rs6748658 (chr2: 16145517), and flanked by rs2287272 (chr2: 15651846) and rs13008990 (chr2: 16150760), the nearest probes which detected normal copy number. The region of gain contained the complete coding sequences of *MYCN* and *DDX1*, and the 5′ region of *NBAS*, and was consistent with the focal gains typically observed in WT samples in our previous SNP array study [11].

### Parallel Evolution of *MYCN* mutations and copy number aberrations in a bilateral WT case

**Figure 2:**
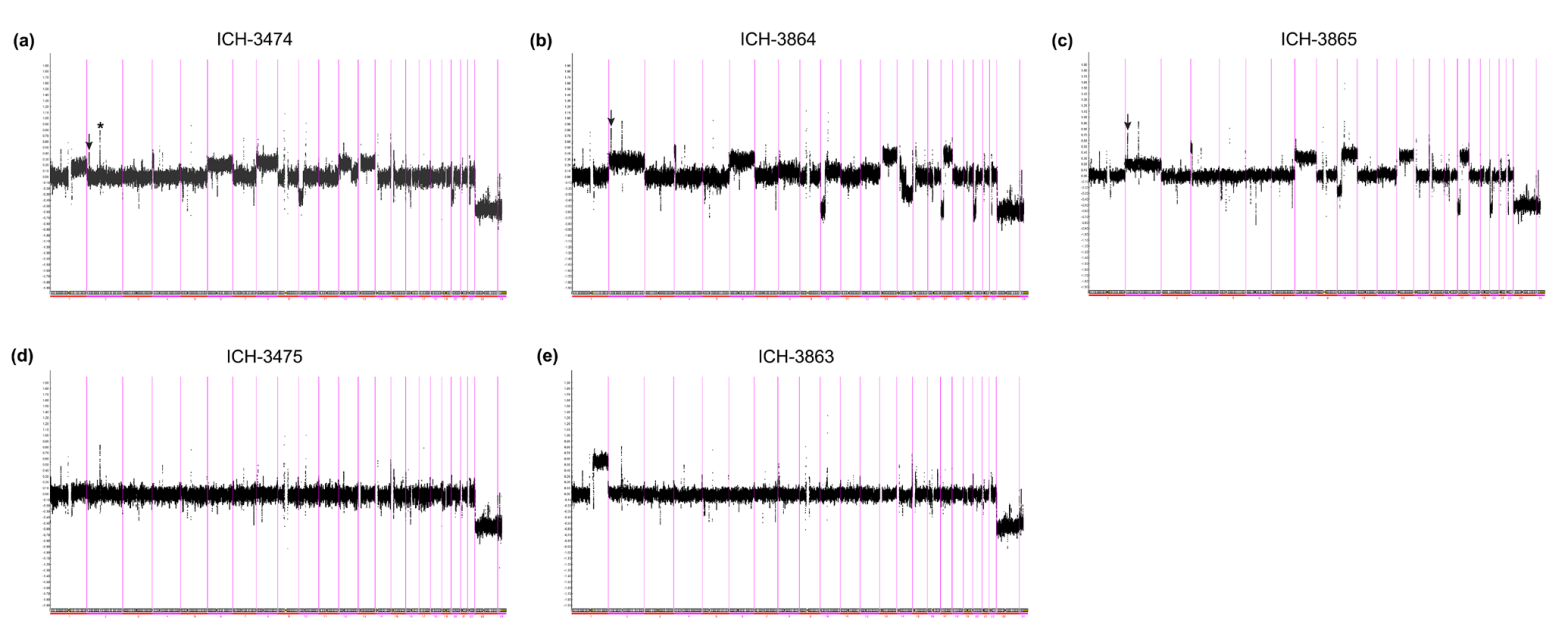
Copy number heterogeneity in a bilateral WT case Affymetrix SNP 6.0 smoothed log2 ratios. (a) ICH-3474 (left, initial biopsy). (b) ICH-3864 (left, partial nephrectomy sample 1). (c) ICH-3865 (left, partial nephrectomy sample 2). (d) ICH-3475 (right, initial biopsy). (e) ICH-3863 (right, partial nephrectomy). Arrows: position of MYCN gain. x-axis: genomic position (divided by chromosome). y-axis: relative copy number (log2 scale). Known copy number variants detected by CNV-specific probes on the SNP 6.0 array were not excluded from this analysis, and are visible as multiple focal peaks in all profiles; the most prominent peak on 2p, labelled ‘*' in panel (a), corresponds to the variable *ANKRD36BP2* locus, and should not be confused with the *MYCN* peak.

One of the samples in which a P44L mutation was detected by exome sequencing was taken from the right side of a second bilateral case we have previously described [36]. This case is notable for its significant intra-tumour heterogeneity on the left side (Figure 2, Supplementary Figure 2, Table 2), and for the evolution of different anaplasia-associated *TP53* mutations in each contralateral tumour. We assessed codon 44 status, major copy number aberrations, and copy neutral loss of heterozygosity (LOH) in multiple samples taken at various time points (Table 2). For *MYCN*, the copy number measurements by SNP array were confirmed by MLPA (as below) in three samples (ICH 3863, ICH-3864 and ICH-3865), which gave fully concordant results. Two spatially separated samples from the initial right partial nephrectomy carried the P44L mutation, and a SNP array of one of these indicated normal *MYCN* copy number. There were no *MYCN* mutations in any other material from the right kidney (Table 2), but as previously reported [36], this tumour carried a p.I195T mutation in *TP53* at relapse, consistent with its anaplastic histology; high quality DNA suitable for copy number analysis was not available for the sample at this time point.

**Table 2:** *MYCN* and TP53 aberrations in a bilateral WT case

ICH ID	Side	Sample	Histology	TP53	MYCN seq data	MYCN CN	Other aberrations	Treatment Month
5179		Blood		wild type	wild type	NA	NA	
3474	Left	Initial biopsy	Wilms tumour	wild type	ND	Gain	+1q, +6, +8, -10p, +12p, +13, -20p	1
6583	Left	Partial nephrectomy	Wilms tumour (stromal area)	wild type	wild type	NA	NA	6
6580	Left	Partial nephrectomy	Wilms tumour (anaplastic area)	c.517G>T: p.173V>L	wild type	NA	NA	6
3864	Left	Partial nephrectomy	Wilms tumour (diffuse anaplastic)	c.517G>T: p.173V>L	wild type	Gain	+2, +6, -10p, +13, -14q, -17p, +17q, -20p	6
3865	Left	Partial nephrectomy	Wilms tumour (diffuse anaplastic)	c.517G>T: p.173V>L	wild type	Gain	+2, LOH 6, +8, -10p, +10q, +13, -17p, +17q, -20p	6
6579	Left	Partial nephrectomy	Normal kidney	wild type	wild type	NA	NA	6
6578	Left	Partial nephrectomy	Nephrogenic rest	wild type	wild type	NA	NA	6
3475	Right	Initial biopsy	Wilms tumour	wild type	wild type	Normal	Neutral profile	1
6581	Right	Partial nephrectomy	Wilms tumour (regressive)	wild type	c.C131T:p.P44L	NA	NA	6
3863	Right	Partial nephrectomy	Wilms tumour (regressive)	wild type	c.C131T:p.P44L	Normal	+1q, LOH 14	6
6582	Right	Partial nephrectomy	Normal kidney	wild type	wild type	NA	NA	6
9934	Right	Partial nephrectomy	Nephrogenic rest	n/a	wild type	NA	NA	6
8426	Right	Recurrence	Wilms tumour (diffuse anaplastic)	c.584T>C: p.I195T	wild type	NA	NA	54

NA = Data not available

In the left kidney, P44 was wild type in all material, including 3 tumour samples that carried the previously reported contralateral p.V173L mutation in *TP53* [36]. SNP arrays were available for two of these samples and from an initial biopsy (Figure 2); all showed clear evidence of *MYCN* gain. Thus, in the contralateral kidneys of a single case with considerable spatial heterogeneity we observed separate *MYCN* aberrations (P44L on the right, copy number gain on the left) and distinct *TP53* mutations (I195T on the right, V173L on the left).

### *MYCN* gain is associated with anaplastic histology and poorer outcome

**Figure 3:**
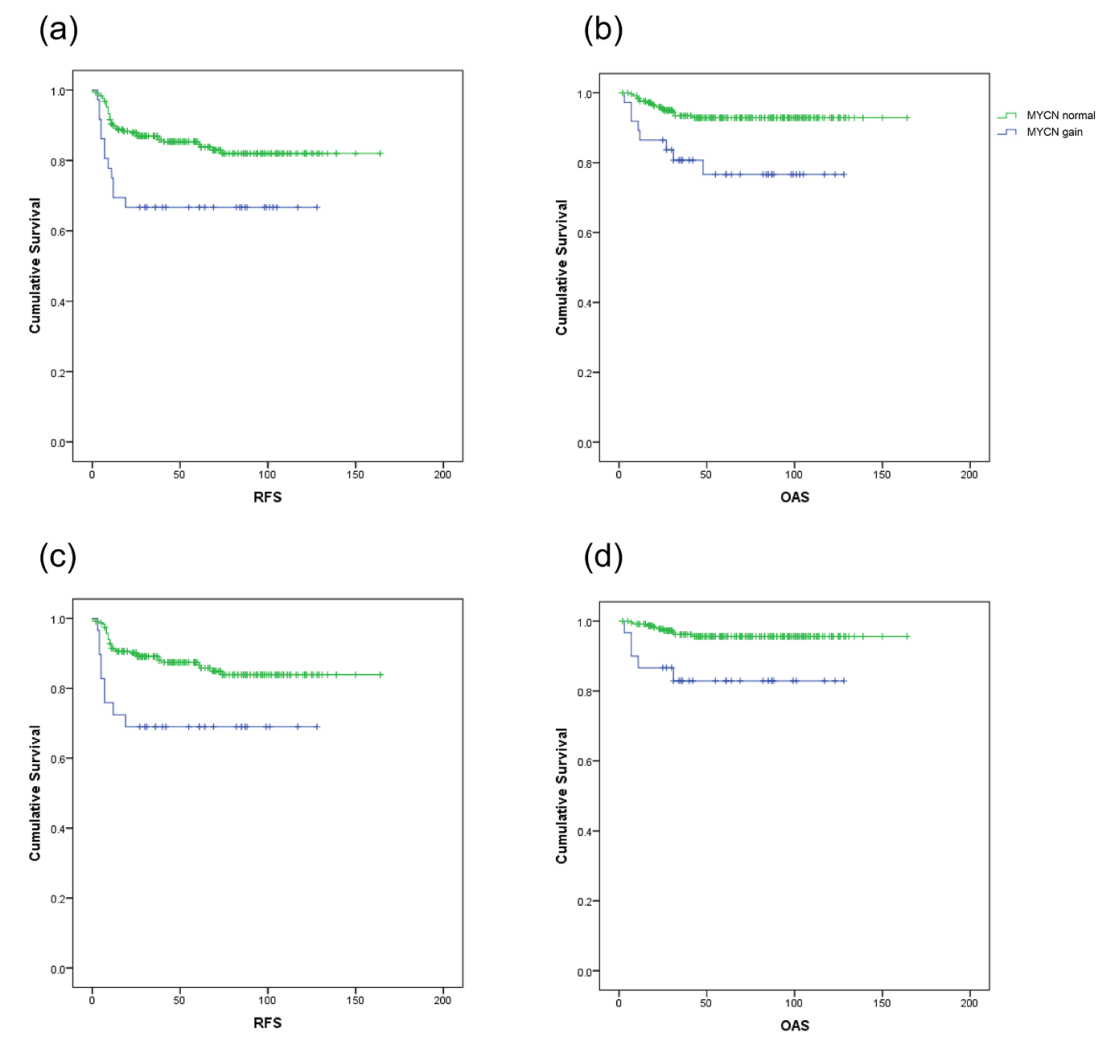
Survival analysis in SIOP patients with or without *MYCN* gain (a) Relapse-free survival (RFS) in cases of all histologies with *MYCN* gain (blue) or with normal *MYCN* copy number (green). (b) Overall survival (OAS) in patients of all histologies. (c) Relapse-free survival in cases with diffuse anaplasia excluded. (d) Overall survival in cases with diffuse anaplasia excluded.

*MYCN* gain was measured in 293 WT cases (Supplementary Table 1) using the P380 Wilms Tumour probemix (gain threshold = 1.2, determined empirically with reference to Affymetrix and Illumina SNP array data). A single sample with copy number loss at the *MYCN* locus was excluded from further analysis. Associations between histological subtype and gain were assessed by two-tailed Fisher's exact test. There was no significant association (p > 0.05) between histology and *MYCN* gain for the blastemal, epithelial, stromal, mixed, regressive or focal anaplastic subtypes. However, for the diffuse anaplastic subtype, where 7/23 (30.4%) samples had *MYCN* gain compared to 30/269 (11.2%) in the remaining series, the association was significant (p = 0.0159). Survival analyses factored by *MYCN* copy number status (gained / normal) were carried out using the Kaplan-Meier model (Figure 3); two samples with no outcome data were excluded, and two further samples from patients who died without a defined relapse event were included in the analyses of overall, but not relapse-free, survival. *MYCN* gain was associated with significantly poorer relapse-free (p = 0.005) and overall (p = 0.002) survival by the Log Rank (Mantel-Cox) test. Using the Cox Proportional Hazards model, we calculated the hazard ratios for *MYCN* gain as 2.443 (relapse) and 3.545 (death). However, since anaplastic histology is itself associated with poor outcome, survival analysis was also carried out with the anaplastic samples removed from the series (Figure 3). Again, the *MYCN* gain group had significantly poorer relapse-free (p = 0.006) and overall (p = 0.003) survival by the Log Rank test, with hazard ratios of 2.697 (relapse) and 4.610 (death). 11 of the 37 samples with *MYCN* gain (2p24.3) also had gain of the *DYSF* locus (control probe) on 2p13.3, indicating that *MYCN* gain is not always focal. We therefore determined whether gain of *DYSF*, as an example of a 2p locus that is distinct from *MYCN*, is associated with a particular subtype or outcome, using the same analyses we applied to *MYCN* (excluding cases with *DYSF* loss). There was no association between *DYSF* gain and any of the histological subtypes, and no significant difference between the relapse free or overall survival of cases with gained or normal *DYSF* at the p < 0.05 level.

### *MYCN* overexpression is associated with hypomethylation of specific loci

**Figure 4:**
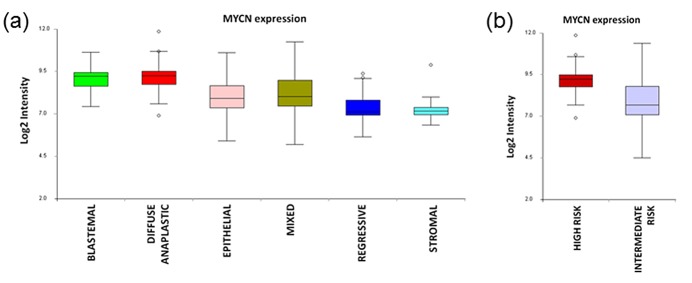
*MYCN* expression in Wilms tumour Box plots of *MYCN* expression in (a) six SIOP histological WT subtypes and (b) with histologies grouped together as high risk or intermediate risk. Values are the mean log2 intensities of expression detected by both Illumina HT-12 probes within each subtype: blastemal type (n = 9, high risk), diffuse anaplastic (n = 11, high risk), epithelial type (n = 9, intermediate risk), mixed type (n = 17, intermediate risk), regressive type (n = 10, intermediate risk) and stromal type (n = 9, intermediate risk). Focal anaplastics were excluded due to insufficient numbers.

**Figure 5:**
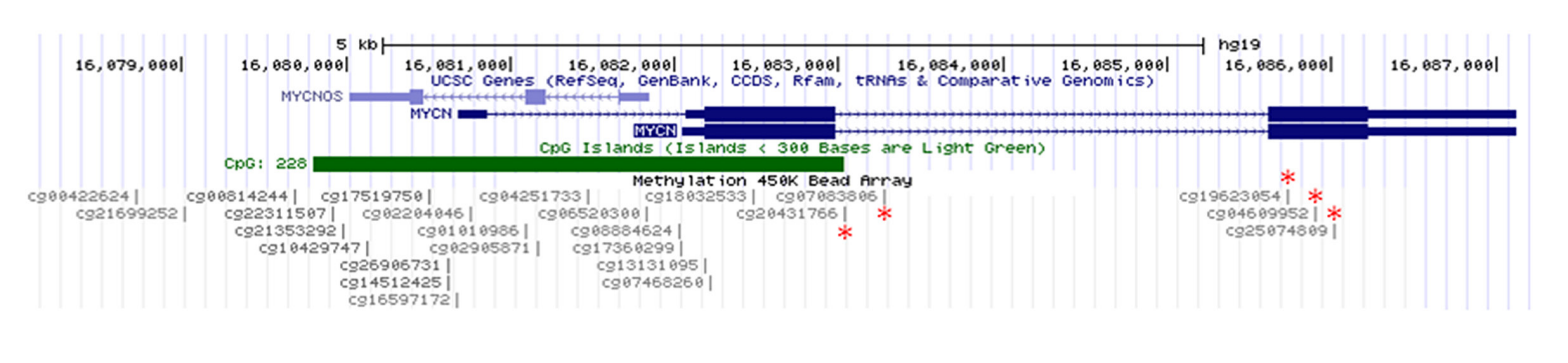
Illumina 450K methylation probes in the *MYCN* region * = locus where expression and methylation are significantly correlated.

In a parallel study in our laboratory, we have recently completed an integrated genomic analysis of WT in which 65 samples were profiled on Affymetrix 250K Nsp / SNP 6.0 arrays for copy number, Illumina HT-12 arrays for mRNA expression, and Illumina 450K arrays for DNA methylation (manuscript in preparation). We therefore examined these data to determine if there is evidence of significant differential regulation of *MYCN* expression in this tumour series. *MYCN* expression levels were assessed in each of six histological subtypes (Figure 4, Supplementary Table 2). Levels of expression appeared notably higher in the high risk diffuse anaplastic (DA) and blastemal type (BT) tumours than in the four intermediate risk (IR: epithelial, stromal, mixed and regressive type) tumours examined. Univariate class comparisons (two-sample t-test) between the IR tumours (taken together as a group) and the tumours in either of the high risk groups identified *MYCN* as a relatively overexpressed gene in both DA and BT WT. When DA tumours were compared with IR tumours at the p < 0.01 significance level, 1786/34339 (5%) of the probes on the array detected differential expression, including both probes for *MYCN* (ILMN_1653761: p = 0.0001, fold-change 2.10; ILMN_2219767: p = 0.0002, fold-change 3.34). Similarly, for BT vs IR, 2521/34339 (7%) of probes detected differential expression, including both *MYCN* probes (ILMN_1653761: p = 0.0019, fold-change 1.66; ILMN_2219767: p = 0.0013, fold-change 2.87). *MYCN* overexpression also appeared to be associated with poorer outcome. Applying the Cox proportional hazards model to the expression data (Wald statistic, p < 0.01) identified 803 probes that detected genes in which overexpression was associated with poorer relapse-free survival (hazard ratio ≥ 1.5), including both *MYCN* probes (ILMN_1653761: p = 0.0082, hazard ratio = 1.9; ILMN_2219767: p = 0.0097, hazard ratio = 1.5). To establish whether the expression patterns we observed were potentially driven by DNA methylation events or by copy number changes, we carried out Spearman rank correlation analyses across the sample series between the expression data (log2 intensity) and either the segmented log2 copy number data, or the logit-transformed methylation value (M value), using all probes with annotation that mapped them to *MYCN*. By this metric we detected only a weak positive correlation between *MYCN* expression and copy number that did not quite reach statistical significance (ILMN_1653761: rho = 0.23, p = 0.0672; ILMN_2219767: rho = 0.24, p = 0.0522). However, we detected a strong negative correlation (rho = −0.50 to −0.71, p < 0.0001) between *MYCN* expression detected by both probes and methylation level at five loci probed by the 450K array (Figure 5). Two of these loci (cg20431766 and cg07083806) are in the intron downstream of the first *MYCN* coding exon, and the more 5′ probe, cg20431766, is within the CpG island that spans the *MYCN* promoter. The other three loci (cg19623054, cg04609952, cg25074809) overlap with the second coding exon. Although the methylation array includes another 18 probes that are annotated to *MYCN* and either overlap with the gene or are located in the intergenic region immediately upstream of its start site, none of these detected differential methylation that significantly correlated with gene expression (Spearman rho < −0.5 or > 0.5; p < 0.05).

## DISCUSSION

The results presented here suggest that MYCN function in Wilms tumour is potentiated by several mechanisms. Copy number gains that included the *MYCN* locus were detected in 37/292 (12.7%) of tumours overall, and in 7/23 (30.4%) of diffuse anaplastic WTs. Relative overexpression of *MYCN* is associated with hypomethylation of two differentially methylated regions within the *MYCN* gene. 8/213 (3.8%) of samples carried a recurrent point mutation, c.131C>T (p.P44L), that has been identified in neuroblastoma as an acquired somatic variant with presumed gain of function [27]. *MYCN* mutations had not previously been described in WT, but during the review of this manuscript another group [37] reported two further examples of the P44L mutation. We note also that our previous discovery of *FBXW7* aberrations in a small proportion of WTs [11] may represent an additional mechanism of MYCN regulation, since FBXW7 is part of a ubiquitin ligase complex that degrades MYCN.

Our results suggest that *MYCN* dysregulation is significantly associated with adverse outcome. *MYCN* gain was associated with high risk diffuse anaplastic histology, and with poorer relapse-free and overall survival, even when diffuse anaplastic cases were excluded from the analysis. This is consistent with a previous study that found an association between *MYCN* expression and relapse [38]. *MYCN* was relatively overexpressed in diffuse anaplastic and blastemal type tumours, and there was an association between expression level and poorer relapse-free survival. However, it is unclear if all potential mechanisms of dysregulation have the same functional effect on downstream pathways or tumour behaviour. The number of P44L mutations detected to date is too small to draw firm conclusions about associations with outcome or histology. A recurrent somatic variant at a single highly conserved residue is most consistent with an activating mutation, though it has not yet been subject to a full functional analysis in WT or in neuroblastoma. We did detect one additional somatic variant, R285W, that is predicted to be deleterious, but its exact functional impact is currently unknown. Three of the P44L mutations were also associated with *MYCN* copy number gain. This may indicate further selection by the tumour cell for multiple copies of a variant that confers a growth advantage.

The case with *MYCN* mutation in one kidney and copy number gain in the other is a striking example of parallel evolution in bilateral disease. As we reported previously [36], this case also has independent contralateral *TP53* mutations, albeit with apparently dissimilar timing of the mutational events. In the left kidney, *MYCN* gain was detected in all specimens examined (Table 2), including the initial pre-chemotherapy biopsy, while the V173L *TP53* mutation was absent at initial biopsy and in a precursor lesion (nephrogenic rest). A copy number profile was not available for the nephrogenic rest, so it was not possible to determine if *MYCN* gain was present in the earliest phase of tumourigenesis, but it is notable that the *MYCN* aberration, together with the large-scale copy number changes on 10p, 20p and 13 that were common to all profiled samples, appear to have preceded disruption of *TP53* by point mutation and 17p loss. In the right kidney, both genes were wild type at initial biopsy, but the *MYCN* P44L mutation was detected at the time of the first (partial) resection, again before any *TP53* aberrations were observed. A subsequent relapse, 4 years after the original tumour was excised, carried the I195T *TP53* mutation, concomitant with the development of anaplasia, but the *MYCN* sequence was wild type. It therefore appears that similar but independent evolutionary solutions affecting two different pathways have been selected for at different stages of the development of contralateral tumours in the same patient, with *MYCN* disruption the earlier event on each side. We note, however, that interpretation of this case is complicated by spatial heterogeneity, which is obviously extensive, at least at the copy number level. It is, for example, not possible to determine from our current analysis whether the relapse in the right kidney, which has a novel *TP53* mutation but lacks the *MYCN* mutation, arose from some minority clone not sampled or undetectable in the first (partial) nephrectomy, or if the relapse itself (of which there was only a single sample) had developed significant spatial heterogeneity. It is even possible that the ‘recurrence' was a novel primary tumour.

*MYCN* gain, even when focal, typically also results in gain of the adjacent *DDX1* gene. All the focal events at this locus analysed by SNP arrays in our previous study [11] had *DDX1* gain, as did the germline gain detected here. Recently, a similar region of germline gain encompassing both genes was detected in a patient with bilateral nephroblastomatosis and a family history of WT [39]. A genome-wide association study has also identified potential WT susceptibility loci in this commonly gained region, mapping to SNPs that flank *DDX1* [40]. We cannot therefore rule out a significant role for *DDX1* in Wilms tumour. However, our finding that *MYCN* is also subject to putatively activating point mutations strongly suggests that *MYCN* is a primary target for dysregulation at this locus.

In addition to neuroblastoma and WT, *MYCN* gain or amplification is now emerging as a common aberration associated with adverse outcome in multiple paediatric malignancies, including rhabdomyosarcoma [26] and medulloblastoma [24, 25]. This raises the possibility that any novel therapies that target the MYCN pathway and prove useful in one of these tumours could be of significant value in the others. In WT, anaplastic tumours that are metastatic at presentation respond poorly to current treatment. While *TP53* mutation remains the most common aberration in this group of tumours, our finding of an association between *MYCN* gain and anaplasia, as well as outcome, makes the MYCN pathway an attractive target for further research into new approaches to treatment.

## MATERIALS AND METHODS

### Clinical samples

Frozen Wilms tumour samples were obtained with informed consent and ethical approval according to national regulations from UK, German, Dutch, and Swedish centres participating in the SIOP WT 2001 clinical study and trial (2001-2011) (Supplementary Table 1). A frozen section was prepared directly or paraffin-embedded from the same specimen used in nucleic acid extraction, stained with haematoxylin and eosin, and assessed for tumour content by an experienced paediatric pathologist. Only samples from WT cases treated by the standard neoadjuvant chemotherapy treatment approach were included in this study.

### Nucleic acid extraction

Frozen tumour material was pulverised at liquid nitrogen temperatures using a Sartorius Mikro-Dismembrator. Genomic DNA was prepared by standard detergent lysis and phenol-chloroform extraction methods. Total RNA was extracted using Trizol reagent (Life Technologies) according to the manufacturer's instructions.

### Whole exome Sequencing

Target enrichment using the Agilent SureSelect V4+UTR system and sequencing on the Illumina HiSeq 2000 instrument were carried out by an external service provider. Data were analysed using a somatic variant detection pipeline on the UCL Computer Science HPC cluster. Sequence reads were aligned to human genome build hg19 using BWA [41]. Removal of PCR duplicates, local realignment around indels, and quality score recalibration were carried out using GATK [42] and Picard (http://picard.sourceforge.net/), according to the GATK ‘best practice' recommendations. Somatic variants were detected with MuTect [43], annotated with ANNOVAR [44], and assessed in IGV [45, 46]. All *MYCN* variants are described with reference to the translational start codon of NCBI RefSeq cDNA NM_005378.4, corresponding to protein NP_005369.2.

### PCR and Sanger sequencing

The complete coding sequence of *MYCN* was amplified from genomic DNA using primers: Ex2Af: 5′- GGTATTAAAACGAACGGGGC-3′; Ex2Ar: 5′-GGACTGGGCGGTGGAAC-3′; Ex2Bf: 5′- ATCCTCCAGGACTGCATGTG-3′; Ex2Br: 5′-CAGGCCAAGACATACGAGC-3′; Ex3Af: 5′- GCCGGAAGAGACAGATAAGC-3′; Ex3Ar: 5′- GATGTTGTGGTTTCTGCGAC-3′; Ex3Bf: 5′- CCACCAGCAGCACAACTATG-3′; Ex3Br: 5′-TACTGCCCACCCAGAGCC-3′. PCR products were sequenced using the ABI BigDye cycle sequencing kit on an ABI Prism 3100 Genetic Analyzer. Variants were detected and assessed with GeneScreen [47] with reference to the hg19 reference sequence.

### MLPA

MLPA was performed according to the manufacturer's instructions using the P380 Wilms Tumour probemix, developed by MRC-Holland in association with our laboratory. This probemix includes 3 probes for *MYCN*, a *DYSF* control probe on 2p outside the common region of genomic gain previously observed on SNP arrays, a *PAX3* control probe on 2q, further test probes to other loci of interest in the Wilms tumour genome, and 9 reference probes to loci on chromosomes 3, 5, 15, 19 and 21 found to have comparatively stable copy number in previous Wilms tumour microarray experiments. Since MYCN binding sites are distributed throughout the genome, we reasoned that the most biologically relevant measurement of *MYCN* status was its copy number relative to the genomic baseline copy number. All copy number measurements were therefore made with respect to the 9 reference probes; the *DYSF* and *PAX3* probes served only to determine whether any *MYCN* gain observed was in the context of larger scale gain of 2p or of the whole chromosome.

### Methylation arrays

DNA methylation profiling was carried out according to the manufacturer's instructions using the Zymo EZ DNA Methylation kit kit and Illumina Infinium HumanMethylation450 BeadChip. Arrays were run and scanned at the UCL Genomics ICH Microarray facility. Data were normalised and analysed using the Bioconductor [48] packages minfi [49] and limma [50].

### Expression arrays

Expression profiling was carried out on the Illumina HumanHT-12 v3 Expression BeadChip at the UCL Genomics ICH Microarray facility, according to the manufacturer's instructions. Data were normalised and analysed using the lumi [51] Bioconductor package and BRB-ArrayTools [52].

### SNP arrays

DNA samples were profiled on Affymetrix Human Mapping 250K Nsp or Genome-Wide Human SNP Array 6.0 arrays at UCL Genomics, UK or AROS Applied Biotechnology A/S, Denmark. Copy number and LOH analysis was carried out using the Affymetrix Genotyping Console, using HapMap reference samples, and visualised in CGH Explorer [53] or IGV [45, 46]. Samples from a single case with germline *MYCN* gain were profiled on the Illumina HumanCytoSNP-12 BeadChip, and analysed with Illumina GenomeStudio and OncoSNP [54]. Raw data are available from the authors on request.

### Survival analysis

Survival analyses were carried out in IBM SPSS 21 using the Kaplan-Meier model, with the Log Rank test for equality of the survival distributions. Hazard ratios were determined using the Cox Proportional Hazards model.

## SUPPLEMENTARY MATERIAL, FIGURES AND TABLES


