# Supplementary material for: Multiple mechanisms of MYCN dysregulation in Wilms tumour
Source: Oncotarget. 2015 Jan 31;6(9):7232–43. doi: 10.18632/oncotarget.3377 (PMC4466681; doi:10.18632/oncotarget.3377)
Supplement: Supplementary file 1 [file oncotarget-06-7232-s001.pdf]

Supplementary Table 1

Sample (ICH) ID	Anon Case ID	Exome data	MYCN codon 44 covered in exome	MYCN Sanger sequencing data	MYCN Variant detected	Germline variant	MLPA data	Relapse free survival (months)	Relapse (1 = relapse; 0 = no relapse)	Overall survival (months)	Death (1 = dead; 0 = alive)	Reviewed histology (AH = anaplastic)	MYCN status	DYSF status
201	2	y	y				y	139	0	139	0	Mixed	normal	normal
205	4	y	y				y	4	1	123	0	Mixed	GAIN	normal
213	21			y (all coding exons)			y	128	0	128	0	Stromal	normal	normal
302	16	y	y				y	2	1	7	1	Blastemal	normal	normal
325	29	y	y				y	8	1	113	0	Regressive	normal	normal
765	35			y (all coding exons)			y	117	0	117	0	Mixed	GAIN	GAIN
1154	332						y	117	0	117	0	Mixed	normal	normal
1159	44			y (all coding exons)			y	113	0	113	0	Stromal	normal	normal
1493	51	y	y				y	105	0	105	0	Mixed	normal	normal
1619	33			y (all coding exons)			y	72	0	72	0	Stromal	normal	normal
1622	7			y (all coding exons)			y	122	0	122	0	Blastemal	normal	normal
1706	334						y	103	0	103	0	Diffuse AH	GAIN	normal
1752	11			y (all coding exons)				113	0	113	0	Mixed		
1761	17			y (all coding exons)				121	0	121	0	Regressive		
1765	12			y (all coding exons)			y	69	1	121	0	Stromal	normal	normal
1769	14			y (all coding exons)			y	128	0	128	0	Stromal	GAIN	GAIN
1770	13	y	y				y	121	0	121	0	Epithelial	normal	normal
1775	24			y (all coding exons)			y	107	0	107	0	Regressive	normal	normal
1779	45	y	y				y	100	0	100	0	Focal AH	normal	normal
1783	28			y (all coding exons)			y	105	0	105	0	Regressive	normal	normal
1784	34			y (all coding exons)			y	59	0	59	0	Epithelial	normal	normal
1785	22			y (all coding exons)			y	106	0	106	0	Mixed	normal	normal
2219	68			y (all coding exons)			y	11	1	31	1	Mixed	normal	normal
2220	65			y (all coding exons)			y	94	0	94	0	Diffuse AH	normal	normal
2261	23			y (all coding exons)			y	8	1	114	0	Regressive	normal	normal
2377	26			y (all coding exons)			y	125	0	125	0	Regressive	normal	normal
2379	20			y (all coding exons)				118	0	118	0	Regressive		
2382	5			y (all coding exons)			y	125	0	125	0	Mixed	normal	normal
2408	77	y	y				y	4	1	7	1	Stromal	GAIN	normal
2409	76			y (all coding exons)			y	94	0	94	0	Stromal	normal	normal
2557	83			y (all coding exons)			y	9	1	11	1	Diffuse AH	normal	normal
2558	75			y (all coding exons)			y	81	0	81	0	Stromal	normal	normal
2560	86	y	y	y (to confirm variant found in exome)	P44L	n	y	98	0	98	0	Focal AH	normal	normal
2594	333						y	10	1	94	0	Epithelial	normal	normal
2649	341						y	120	0	120	0	Mixed	normal	normal
2652	31			y (all coding exons)			y	111	0	111	0	Mixed	normal	normal
2655	48			y (all coding exons)				105	0	105	0	Regressive		
2663	3			y (all coding exons)			y	116	0	116	0	Stromal	normal	normal
2664	344						y	106	0	106	0	Mixed	normal	normal
2665	30			y (all coding exons)			y	9	1	96	0	Epithelial	normal	normal
2670	52			y (all coding exons)				91	0	91	0	Stromal		
2714	37	y	y				y	121	0	121	0	Mixed	normal	normal
2716	10			y (all coding exons)			y	26	1	42	1	Blastemal	normal	normal
2717	59	y	y				y	90	0	90	0	Blastemal	normal	normal
2720	55	y	y				y	101	0	101	0	Blastemal	normal	normal
2732	95			y (all coding exons)			y	87	0	87	0	Regressive	GAIN	normal
2733	93			y (all coding exons)			y	96	0	96	0	Mixed	normal	normal
2734	91			y (all coding exons)				95	0	95	0	Regressive		
2964	74			y (all coding exons)			y	12	0	12	0	Mixed	normal	normal
2967	78			y (all coding exons)				5	1	6	1	Diffuse AH		
2969	89	y	y				y	9	1	12	1	Diffuse AH	GAIN	normal
2970	338						y	96	0	96	0	Regressive	normal	normal
2987	82			y (all coding exons)			y	96	0	96	0	Regressive	normal	normal
2990	98	y	y				y	61	1	87	0	Epithelial	normal	normal
2994	96			y (all coding exons)			y	88	0	88	0	Regressive	normal	normal
3010	66			y (all coding exons)			y	104	0	104	0	Stromal	normal	normal
3020	79			y (all coding exons)			y	5	1	7	1	Blastemal	GAIN	normal
3133	8			y (all coding exons)			y	66	0	66	0	Diffuse AH	normal	normal
3136	15			y (all coding exons)	R285W	y	y	102	0	102	0	Stromal	normal	normal
3140	25	y	y				y	97	0	97	0	Stromal	normal	normal
3141	36	y	y				y	111	0	111	0	Stromal	normal	normal
3142	39	y	y				y	113	0	113	0	Stromal	normal	normal
3143	40	y	n				y	105	0	105	0	Diffuse AH	GAIN	GAIN
3144	41			y (all coding exons)			y	108	0	108	0	Diffuse AH	normal	normal

3145	43	y	y				y	18	1	19	1	Diffuse AH	normal	normal
3146	47			y (all coding exons)			y	86	0	86	0	Regressive	normal	normal
3147	54	y	y				y	97	0	97	0	Epithelial	normal	normal
3148	72	y	y				y	38	1	93	0	Mixed	normal	normal
3150	340						y	78	0	78	0	Regressive	normal	normal
3152	97			y (all coding exons)				71	0	71	0	Stromal		
3156	19			y (all coding exons)			y	94	0	94	0	Regressive	normal	normal
3160	38			y (all coding exons)			y	73	0	73	0	Stromal	normal	normal
3162	90			y (all coding exons)			y	11	1	57	0	Stromal	normal	normal
3166	94	y	y					65	0	65	0	Stromal		
3201	336						y	99	0	99	0	Regressive	normal	normal
3477	108	y	y				y	84	0	84	0	Diffuse AH	GAIN	normal
3738	70			y (all coding exons)			y	70	0	70	0	Regressive	normal	normal
3745	335						y	97	0	97	0	Stromal	normal	normal
3747	46	y	y					108	0	108	0	Mixed		
3750	6			y (NB: 2nd coding exon incompletely covered)			y	129	0	129	0	Mixed	normal	normal
3751	339						y	87	0	87	0	Mixed	normal	normal
3863	107	y	y	y (to confirm variant found in exome)	P44L	n		52	1	88	0	Regressive		
3871	100	y	y				y	90	0	90	0	Focal AH	normal	normal
3886	102			y (all coding exons)			y	11	1	20	1	Epithelial	normal	normal
4081	27						y	22	1	125	0	Mixed	normal	normal
4083	27	y	y					22	1	125	0	Mixed		
4092	67	y	y				y	74	1	101	0	Regressive	normal	normal
4212	117			y (all coding exons)				66	0	66	0	Regressive		
4624	124			y (all coding exons)			y	69	0	69	0	Stromal	normal	normal
4632	126	y	y					40	0	40	0	Stromal		
4644	71			y (all coding exons)			y			3	1	Mixed	GAIN	normal
4646	85			y (all coding exons)			y	98	0	98	0	Stromal	normal	normal
4648	88			y (all coding exons)			y	93	0	93	0	Regressive	normal	normal
4667	92			y (all coding exons)			y	6	1	87	0	Diffuse AH	normal	normal
4671	112	y	y				y	73	0	73	0	Focal AH	normal	normal
4674	115	y	y				y	75	0	75	0	Blastemal	normal	normal
4681	128			y (all coding exons)			y	61	0	61	0	Mixed	GAIN	GAIN
4686	53			y (all coding exons)			y	105	0	105	0	Epithelial	normal	normal
4692	113			y (all coding exons)	S76N	N/A		74	0	74	0	Regressive		
4706	106	y	y					38	1	80	0	Regressive		
4709	110	y	n				y	85	0	85	0	Epithelial	normal	normal
4714	337						y	92	0	92	0	Stromal	normal	normal
4718	121	y	y				y	11	1	27	1	Diffuse AH	GAIN	normal
4721	60	y	y					9	1	105	0	Mixed		
4722	60						y	9	1	105	0	Mixed	normal	normal
4733	104			y (all coding exons)			y	70	0	70	0	Mixed	normal	normal
4735	114	y	y				y	10	1	53	0	Epithelial	normal	normal
4737	116			y (all coding exons)			y	54	0	54	0	Regressive	normal	normal
4759	343						y	122	0	122	0	Epithelial	normal	normal
4765	99			y (all coding exons)			y	48	0	48	0	Epithelial	normal	normal
4773	131			y (all coding exons)			y	69	0	69	0	Mixed	normal	normal
4778	61			y (all coding exons)			y	88	0	88	0	Mixed	normal	normal
4783	127			y (all coding exons)			y	66	0	66	0	Stromal	normal	normal
4788	81			y (all coding exons)	P44L	n		86	0	86	0	Regressive		
4802	342						y	80	0	80	0	Blastemal	normal	normal
4807	120			y (all coding exons)			y	67	0	67	0	Mixed	normal	normal
4897	132	y	y				y	28	0	28	0	Stromal	normal	normal
4912	103			y (all coding exons)			y	15	1	25	1	Diffuse AH	normal	normal
4914	105			y (all coding exons)				79	0	79	0	Regressive		
4918	111	y	y					88	0	88	0	Mixed		
4928	119			y (all coding exons)				69	0	69	0	Mixed		
4934	123			y (all coding exons)			y	70	0	70	0	Mixed	normal	normal
4950	135			y (all coding exons)				62	0	62	0	Regressive		
4956	136			y (all coding exons)	P44L	n		55	0	55	0	Regressive		
4968	137			y (all coding exons)			y	62	0	62	0	Regressive	normal	normal
5249	73			y (all coding exons)				19	1	98	0	Regressive		
5252	84			y (all coding exons)				15	1	26	1	Stromal		
5254	87			y (all coding exons)			y	62	1	100	0	Mixed	normal	normal
5258	101			y (all coding exons)			y	83	0	83	0	Mixed	normal	normal
5260	109			y (all coding exons)				81	0	81	0	Stromal		
5666	69	y	n				y	12	1	32	1	Diffuse AH	normal	normal
6601	141			y (all coding exons)				44	0	44	0	Mixed		

6603	141						y	44	0	44	0	Mixed	normal	normal
6613	144			y (all coding exons)	P44L	n		42	0	42	0	Mixed		
6614	144						y	42	0	42	0	Mixed	GAIN	normal
6993	147	y	y				y	0	1	54	0	Stromal	normal	normal
7000	153			y (all coding exons)				44	0	44	0	Diffuse AH		
7422	138			y (all coding exons)				64	0	64	0	Regressive		
7426	63	y	y				y	8	1	97	0	Regressive	normal	normal
7432	58			y (all coding exons)				97	0	97	0	Epithelial		
7434	56			y (all coding exons)			y	102	0	102	0	Regressive	normal	normal
7436	9			y (all coding exons)			y	131	0	131	0	Mixed	normal	normal
7462	182			y (all coding exons)			y	94	0	94	0	Mixed	normal	normal
7464	183			y (all coding exons)			y	99	0	99	0	Mixed	GAIN	normal
7478	184			y (all coding exons)			y	112	0	112	0	Blastemal	normal	normal
7482	185			y (all coding exons)			y	7	1	87	0	Regressive	GAIN	GAIN
7484	186			y (all coding exons)			y	86	0	86	0	Mixed	normal	normal
7488	187			y (all coding exons)			y	55	0	55	0	Stromal	normal	normal
7490	188			y (all coding exons)			y	85	0	85	0	Mixed	GAIN	normal
7492	189			y (all coding exons)			y	88	0	88	0	Regressive	normal	normal
7498	190			y (all coding exons)			y	58	0	58	0	Mixed	normal	normal
7502	191			y (all coding exons)				57	0	57	0	Stromal		
7506	192			y (all coding exons)			y	39	0	39	0	Mixed	normal	normal
7512	195			y (all coding exons)	A158V	y	y	64	0	64	0	Stromal	GAIN	GAIN
7514	194			y (all coding exons)			y	71	0	17	1	Mixed	normal	normal
7533	196			y (all coding exons)			y	64	0	64	0	Epithelial	normal	normal
7537	197			y (all coding exons)			y	25	0	25	0	Diffuse AH	normal	normal
7539	200			y (all coding exons)			y	12	1	48	1	Diffuse AH	GAIN	GAIN
7541	198			y (all coding exons)			y	7	1	26	1	Regressive	normal	normal
7545	199			y (all coding exons)			y	69	0	69	0	Epithelial	normal	normal
7552	201			y (all coding exons)			y	47	0	47	0	Stromal	normal	normal
7555	202			y (all coding exons)			y	59	0	59	0	Stromal	normal	normal
7561	206			y (all coding exons)			y	51	0	51	0	Regressive	normal	normal
7563	204			y (all coding exons)			y	53	0	53	0	Stromal	normal	normal
7567	207			y (all coding exons)				1	1	9	1	Diffuse AH		
7570	209			y (all coding exons)			y	45	0	45	0	Regressive	normal	normal
7574	212			y (all coding exons)			y	39	0	39	0	Diffuse AH	normal	normal
7578	215			y (all coding exons)			y	19	1	31	1	Blastemal	GAIN	normal
7582	211			y (all coding exons)				17	1	19	1	Diffuse AH		
7584	218			y (all coding exons)			y	35	0	35	0	Mixed	normal	normal
7595	203			y (all coding exons)			y	11	0	11	1	Diffuse AH	normal	normal
7597	205			y (all coding exons)				39	0	39	0	Mixed		
7601	193			y (all coding exons)			y	10	0	10	0	Stromal	normal	normal
7605	208			y (all coding exons)				33	0	33	0	Mixed		
7609	210			y (all coding exons)				31	0	31	0	N/A		
7613	216			y (all coding exons)			y	23	0	23	0	Mixed	normal	normal
7615	213			y (all coding exons)			y	29	0	29	0	Mixed	normal	normal
7617	214			y (all coding exons)			y	4	1	12	1	Diffuse AH	normal	normal
7621	219			y (all coding exons)				16	0	16	0	Mixed		
7623	217			y (all coding exons)			y	20	0	20	0	Stromal	normal	normal
7639	309						y	129	0	129	0	Regressive	normal	normal
7640	312						y	121	0	121	0	Mixed	normal	normal
7642	310						y	86	0	86	0	Mixed	normal	normal
7643	311						y	72	0	72	0	Blastemal	normal	normal
7645	313						y	109	0	109	0	Stromal	normal	normal
7650	314						y	164	0	164	0	Blastemal	normal	normal
7655	316						y	108	0	108	0	Mixed	normal	normal
7660	315						y	102	0	102	0	Diffuse AH	normal	normal
7662	308						y	5	1	11	1	Blastemal	GAIN	normal
7674	317						y	150	0	150	0	Regressive	normal	normal
7678	221						y	69	0	69	0	Mixed	normal	normal
7679	318						y	134	0	134	0	Mixed	normal	normal
7680	220						y	62	0	62	0	Epithelial	normal	normal
7682	222						y	139	0	139	0	Blastemal	normal	normal
7683	223						y	105	0	105	0	Regressive	normal	normal
7684	224						y	83	0	83	0	Mixed	normal	normal
7691	225						y	126	0	126	0	Regressive	normal	normal
7693	226						y	122	0	122	0	Mixed	normal	normal
7698	227						y	41	1	126	0	Focal AH	normal	normal
7708	228						y	101	0	101	0	Mixed	GAIN	GAIN
7725	230						y	9	1	105	0	Regressive	normal	normal
7726	229						y	70	0	70	0	Regressive	normal	normal
7729	231						y	61	0	61	0	Regressive	GAIN	normal
7854	232						y	82	0	82	0	Mixed	GAIN	normal

7893	233						y	88	0	88	0	Stromal	GAIN	normal
7927	234						y	80	0	80	0	Stromal	normal	normal
7935	235						y	69	0	69	0	Regressive	GAIN	normal
7941	236						y	11	0	11	0	Stromal	normal	normal
7952	237						y	74	0	74	0	Regressive	normal	normal
8011	238						y	62	0	62	0	Regressive	LOSS	LOSS
8014	239						y	61	0	61	0	Stromal	normal	normal
8016	240						y	62	0	62	0	Blastemal	normal	normal
8024	242						y	58	0	58	0	Regressive	normal	normal
8025	241						y	62	0	62	0	Regressive	normal	normal
8030	243						y	62	0	62	0	Mixed	normal	normal
8043	244						y	60	0	60	0	Stromal	normal	normal
8052	245						y	58	0	58	0	Stromal	normal	normal
8066	246						y	47	0	47	0	Mixed	normal	normal
8067	247						y	43	0	43	0	Stromal	normal	normal
8075	249						y	58	0	58	0	Mixed	normal	normal
8077	250						y	55	0	55	0	Stromal	normal	normal
8094	248						y	50	0	50	0	Stromal	normal	normal
8099	251						y	53	0	53	0	Stromal	normal	normal
8101	252						y	55	0	55	0	Regressive	GAIN	normal
8102	253						y	52	0	52	0	Stromal	normal	normal
8109	254						y	50	0	50	0	Mixed	normal	LOSS
8112	256						y	37	0	37	0	Mixed	normal	LOSS
8113	255						y	6	1	29	0	Regressive	normal	normal
8122	258						y	49	0	49	0	Regressive	normal	normal
8125	259						y	28	0	28	0	Regressive	normal	LOSS
8127	257						y	52	0	52	0	Blastemal	normal	normal
8128	260						y	45	0	45	0	Mixed	normal	LOSS
8143	261						y	26	0	26	0	Regressive	normal	normal
8146	262						y	45	0	45	0	Mixed	normal	normal
8157	263						y	46	0	46	0	Regressive	normal	normal
8162	266						y	43	0	43	0	Mixed	normal	normal
8163	264						y	41	0	41	0	Stromal	normal	LOSS
8170	265						y	43	0	43	0	Regressive	normal	LOSS
8180	267						y	41	0	41	0	Mixed	normal	LOSS
8187	268						y	29	0	29	0	Stromal	normal	normal
8197	270						y	37	0	37	0	Mixed	normal	normal
8200	269						y	38	0	38	0	Regressive	normal	normal
8209	272						y	28	0	28	0	Regressive	normal	normal
8217	273						y	36	0	36	0	Regressive	GAIN	normal
8222	274						y	32	0	32	0	Regressive	normal	normal
8225	275						y	15	0	15	0	Regressive	normal	normal
8232	279						y	15	0	15	0	Mixed	normal	LOSS
8233	276						y	20	0	20	0	Mixed	normal	normal
8236	277						y	18	0	18	0	Stromal	normal	normal
8243	278						y	30	0	30	0	Regressive	normal	normal
8248	280						y	30	0	30	0	Regressive	normal	normal
8249	281						y	30	0	30	0	Mixed	normal	normal
8252	282						y	2	0	2	0	Regressive	normal	normal
8254	283						y	18	0	18	0	Regressive	normal	normal
8255	287						y	27	0	27	0	Mixed	GAIN	normal
8257	285						y	26	0	26	0	Regressive	normal	normal
8267	291						y	26	0	26	0	Regressive	normal	normal
8268	284						y	26	0	26	0	Stromal	normal	LOSS
8269	288						y	26	1	28	0	Blastemal	normal	LOSS
8271	289						y	3	1	25	0	Regressive	GAIN	LOSS
8272	293						y	26	0	26	0	Mixed	normal	normal
8274	292						y	27	0	27	0	Regressive	normal	normal
8277	286						y	27	0	27	0	Stromal	normal	normal
8278	271						y					Regressive	normal	normal
8281	294						y	24	0	24	0	Stromal	normal	normal
8282	296						y	23	0	23	0	Mixed	normal	normal
8286	295						y	24	0	24	0	Blastemal	normal	normal
8287	290						y	25	0	25	0	Mixed	normal	normal
8293	297						y	23	0	23	0	Blastemal	normal	normal
8300	298						y	17	0	17	0	Mixed	normal	normal
8304	300						y	7	1	19	0	Mixed	normal	normal
8305	299						y	18	0	18	0	Regressive	normal	normal
8307	301						y	17	0	17	0	Mixed	normal	normal
8308	304						y	18	0	18	0	Mixed	normal	LOSS
8310	303						y	10	1	17	0	Diffuse AH	normal	LOSS
8311	305						y	8	1	19	0	Mixed	normal	normal

8312	302						y	5	0	5	0	Mixed	normal	LOSS
8313	306						y	17	0	17	0	Regressive	normal	normal
8314	307						y	4	1	17	0	Mixed	normal	normal
8702	175	y	n				y	31	0	31	0	Stromal	GAIN	GAIN
8774	176	y	n				y	30	0	30	0	Stromal	normal	normal
8783	178	y	y	y (to confirm variant found in exome)	P44L	n	y	25	0	25	0	Blastemal	normal	normal
8817	345						y	26	0	26	0	Regressive	normal	normal
8819	180			y (all coding exons)	P44L	n		26	0	26	0	Regressive		
8834	177			y (all coding exons)				30	0	30	0	Mixed		
8837	177						y	30	0	30	0	Mixed	GAIN	normal
8853	179	y	y							8	1	Mixed		
8855	179						y			8	1	Mixed	normal	normal
8860	346						y	25	0	25	0	Mixed	normal	normal
8890	181			y (all coding exons)				26	0	26	0	Mixed		
8893	181						y	26	0	26	0	Mixed	normal	normal
8896	1	y	y									N/A		
8903	166			y (all coding exons)			y	36	0	36	0	Regressive	GAIN	normal
8914	171			y (all coding exons)	P63P	ND	y	7	1	34	0	Mixed	GAIN	normal
8922	165			y (all coding exons)				33	0	33	0	Regressive		
8925	172			y (all coding exons)			y	12	1	35	0	Mixed	GAIN	GAIN
9048	161			y (all coding exons)			y	45	0	45	0	Diffuse AH	normal	normal
9115	159			y (all coding exons)						1	1	Regressive		
9118	168			y (all coding exons)			y	36	0	36	0	Stromal	normal	normal
9127	173	y	y					35	0	35	0	Mixed		
9128	173						y	35	0	35	0	Mixed	normal	normal
9132	167			y (all coding exons)			y	38	0	38	0	Mixed	normal	normal
9140	157			y (all coding exons)	E69E	ND	y	43	0	43	0	Regressive	normal	normal
9161	49			y (all coding exons)			y	109	0	109	0	Epithelial	normal	normal
9165	80			y (all coding exons)			y	98	0	98	0	Diffuse AH	GAIN	normal
9173	118			y (all coding exons)			y	76	0	76	0	Epithelial	normal	normal
9179	125			y (all coding exons)				73	0	73	0	Stromal		
9189	64	y	y					17	1	17	1	Diffuse AH		
9197	169	y	y				y	35	0	35	0	Blastemal	normal	normal
9219	163			y (all coding exons)			y	30	0	30	0	Regressive	normal	normal
9224	162			y (all coding exons)	E69E	ND		32	0	32	0	Regressive		
9413	140			y (all coding exons)				53	0	53	0	Blastemal		
9415	139			y (all coding exons)				56	0	56	0	Mixed		
9431	164			y (all coding exons)	P365A	N/A		39	0	39	0	Regressive		
9442	133	y	n				y	59	0	59	0	Mixed	normal	normal
9455	134			y (all coding exons)			y	58	0	58	0	Mixed	normal	normal
9460	50			y (all coding exons)				14	1	111	0	Stromal		
9462	170			y (all coding exons)				36	0	36	0	Mixed		
9532	145	y	y				y	40	0	40	0	Focal AH	GAIN	GAIN
9537	158			y (all coding exons)				32	0	32	0	Diffuse AH		
9543	142			y (all coding exons)			y	46	0	46	0	Stromal	normal	normal
9547	156			y (all coding exons)				11	0	17	1	N/A		
9554	18			y (all coding exons)			y	87	0	87	0	Stromal	normal	normal
9574	155			y (all coding exons)			y	44	0	44	0	Mixed	normal	normal
9629	152			y (all coding exons)			y	14	0	14	0	Epithelial	normal	normal
9645	32			y (all coding exons)				124	0	124	0	Regressive		
9647	42			y (all coding exons)			y	13	1	102	0	Stromal	normal	normal
9657	57			y (all coding exons)			y	12	0	12	1	Diffuse AH	normal	normal
9660	62			y (all coding exons)			y	108	0	108	0	Stromal	normal	normal
9682	122			y (all coding exons)			y	14	1	23	1	Regressive	normal	normal
9702	129			y (all coding exons)			y	38	1	76	0	Epithelial	normal	normal
9707	130			y (all coding exons)			y	66	0	66	0	Mixed	normal	normal
9709	148			y (all coding exons)			y	75	0	75	0	Blastemal	normal	normal
9742	154			y (all coding exons)			y	46	0	46	0	Stromal	normal	normal
9748	151			y (all coding exons)			y	50	0	50	0	Stromal	normal	normal
9750	146			y (all coding exons)	P44L	n		54	0	54	0	Mixed		
9755	160			y (all coding exons)			y	43	0	43	0	Mixed	normal	normal
9759	149			y (all coding exons)			y	49	0	49	0	Epithelial	normal	normal
9989	143			y (all coding exons)				43	0	43	0	Mixed		
9991	150			y (all coding exons)				40	0	40	0	Mixed		
10001	174			y (all coding exons)				23	0	23	0	Stromal		
11062	324						y	67	0	67	0	Mixed	normal	normal
11063	328						y	15	0	15	0	Regressive	normal	normal
11065	323						y	10	1	32	1	Focal AH	normal	normal
11066	322						y	81	0	81	0	Mixed	normal	normal
11071	320						y	9	1	103	0	Regressive	normal	normal

11072	327						y	74	0	74	0	Mixed	normal	normal
11073	329						y	69	0	69	0	Blastemal	normal	normal
11075	331						y	75	0	75	0	Focal AH	normal	normal
11077	325						y	31	0	31	0	Epithelial	normal	normal
11078	330						y	46	0	46	0	Regressive	normal	normal
11079	321						y					Stromal	normal	normal
11086	326						y	11	0	11	0	Regressive	normal	normal
11362	319						y	7	0	7	0	Regressive	normal	normal

Supplementary Table 2

				Expression log2 intensity	Expression log2 intensity	Segmented log2 copy number	methylation (M)	methylation (M)	methylation (M)	methylation (M)	methylation (M)	methylation (M)	methylation (M)	methylation (M)
ICH ID	Histology	RFS (months)	Relapse	ILMN_2219767	ILMN_1653761	MYCN	cg00814244	cg22311507	cg21353292	cg10429747	cg17519750	cg26906731	cg14512425	cg16597172
201	Mixed	126	0	7.871	6.961	1.000	-4.104	-5.471	-4.993	-5.301	-5.362	-5.505	-3.847	-4.376
205	Mixed	4	1	9.750	7.773	1.000	-4.330	-5.430	-4.371	-4.326	-4.778	-4.488	-3.396	-4.276
302	Blastemal	2	1	10.194	8.178	1.000	-4.160	-5.268	-4.476	-5.048	-4.628	-4.761	-3.598	-4.540
325	Regressive	8	1	6.964	6.859	1.000	-4.300	-4.807	-4.276	-4.687	-4.694	-4.604	-3.371	-4.061
1159	Stromal	90	0	7.276	6.913	1.000	-3.788	-5.832	-4.378	-4.857	-4.631	-4.395	-3.205	-4.699
1493	Mixed	93	0	11.604	9.453	1.000	-3.918	-5.294	-4.416	-4.347	-4.120	-4.101	-3.327	-4.071
1706	Diffuse Anaplastic	90	0	10.183	8.340	1.585	-4.661	-5.104	-6.335	-4.703	-5.154	-5.056	-3.237	-4.915
1784	Epithelial	59	0	7.255	6.899	1.000	-3.803	-5.740	-5.079	-4.341	-4.108	-4.540	-3.556	-4.175
2220	Diffuse Anaplastic	94	0	7.009	6.826	1.000	-3.541	-4.748	-4.744	-4.367	-3.816	-4.386	-3.501	-4.203
2261	Regressive	8	1	9.814	8.481	1.000	-4.609	-5.980	-7.637	-4.442	-5.159	-4.965	-3.580	-4.039
2382	Mixed	125	0	10.223	7.983	1.000	-3.737	-5.170	-3.657	-4.397	-3.844	-4.436	-3.358	-3.677
2388	Regressive	111	0	6.958	6.833	1.000	-4.284	-4.827	-5.041	-4.644	-4.188	-4.332	-3.366	-4.140
2557	Diffuse Anaplastic	9	1	9.797	7.747	1.000	-3.921	-5.526	-4.033	-4.226	-4.500	-4.250	-3.558	-4.261
2594	Epithelial	10	1	10.701	8.018	1.000	-4.043	-6.160	-4.530	-4.988	-4.792	-5.009	-3.823	-4.762
2652	Mixed	111	0	8.211	7.854	1.000	-4.532	-5.211	-4.902	-4.728	-4.559	-4.613	-3.346	-4.513
2663	Stromal	116	0	6.907	6.811	1.000	-3.704	-5.982	-5.529	-4.809	-4.822	-4.717	-3.331	-4.632
2664	Mixed	106	0	7.624	7.017	1.000	-3.921	-4.668	-4.735	-4.096	-4.375	-4.400	-3.743	-4.031
2716	Blastemal	26	1	10.125	8.353	1.000	-3.769	-5.214	-4.827	-5.080	-4.300	-4.435	-3.368	-4.132
2717	Blastemal	90	0	10.303	7.867	1.000	-3.891	-4.369	-4.167	-4.338	-4.067	-4.171	-3.185	-4.255
2720	Blastemal	101	0	10.264	8.388	1.000	-4.153	-5.087	-4.253	-4.266	-4.186	-4.319	-3.436	-4.079
2734	Regressive	83	0	6.976	6.994	1.000	-4.513	-5.225	-4.796	-4.409	-4.521	-4.470	-3.348	-4.422
2964	Mixed	12	0	9.866	7.878	1.585	-4.124	-4.816	-3.734	-3.912	-3.990	-4.392	-3.456	-3.930
2967	Diffuse Anaplastic	5	1	12.568	11.195	3.000	-2.745	-4.817	-5.600	-2.873	-5.333	-3.658	-2.531	-3.043
2969	Diffuse Anaplastic	9	1	9.687	7.738	2.322	-4.316	-5.351	-5.129	-4.588	-4.879	-4.729	-3.093	-4.339
2970	Regressive	86	0	7.111	6.805	1.000	-4.638	-5.761	-4.690	-4.684	-4.367	-4.490	-3.254	-4.573
2987	Regressive	82	0	10.539	8.255	1.000	-4.066	-5.154	-4.677	-4.627	-4.304	-4.306	-3.517	-3.949
2990	Epithelial	61	1	8.424	7.514	1.000	-3.569	-5.147	-4.326	-4.376	-4.014	-4.355	-3.348	-4.121
2994	Epithelial	75	0	8.235	7.323	1.000	-3.591	-5.968	-5.066	-4.253	-4.102	-4.052	-3.479	-4.095
3010	Stromal	87	0	7.740	7.008	1.000	-3.889	-4.906	-3.709	-4.352	-4.251	-4.426	-3.340	-4.010
3020	Blastemal	5	1	10.994	8.593	2.000	-4.145	-5.123	-4.795	-4.179	-4.177	-4.318	-3.496	-4.190
3136	Stromal	102	0	7.772	7.034	1.000	-4.575	-4.949	-5.972	-4.582	-4.414	-4.645	-3.420	-4.570
3140	Stromal	97	0	7.382	6.808	1.585	-4.626	-8.044	-4.712	-4.702	-4.022	-4.478	-3.234	-4.138
3143	Diffuse Anaplastic	105	0	10.464	8.623	1.000	-3.878	-8.101	-4.752	-4.526	-4.451	-4.323	-3.426	-4.183
3145	Diffuse Anaplastic	18	1	10.448	8.439	1.000	-3.551	-4.804	-3.819	-4.352	-4.035	-4.593	-3.553	-3.868
3148	Mixed	38	1	7.781	7.032	1.000	-4.031	-5.157	-5.111	-4.623	-4.250	-4.235	-3.324	-4.429
3150	Regressive	78	0	7.157	6.896	1.000	-4.417	-5.347	-4.525	-4.098	-4.328	-4.085	-3.278	-4.300
3477	Diffuse Anaplastic	71	0	12.147	9.263	1.585	-3.975	-4.859	-5.998	-4.807	-4.569	-4.470	-2.868	-3.980
3738	Regressive	70	0	8.452	7.175	1.000	-3.980	-5.498	-4.705	-5.137	-5.046	-5.137	-3.559	-4.822
3745	Stromal	84	0	6.918	6.705	1.000	-3.899	-5.572	-4.589	-5.165	-4.853	-4.588	-3.193	-4.254
3750	Mixed	116	0	7.918	7.205	1.000	-3.894	-5.124	-5.480	-4.915	-4.897	-4.879	-3.716	-4.500
3751	Mixed	85	0	9.122	7.702	1.000	-4.153	-5.141	-4.127	-4.231	-4.315	-4.789	-3.403	-3.961
3864	Diffuse Anaplastic	52	1	9.639	8.345	2.000	-4.122	-4.962	-4.626	-4.479	-4.775	-4.403	-3.200	-4.028
3886	Epithelial	11	1	7.380	6.634	1.000	-4.220	-6.043	-5.745	-4.911	-4.668	-4.514	-3.599	-4.665
4083	Blastemal	22	1	8.995	7.424	1.000	-3.956	-6.488	-4.416	-5.008	-5.054	-4.653	-3.472	-3.883
4092	Regressive	74	1	8.224	7.283	1.000	-3.883	-5.357	-6.006	-5.247	-3.688	-4.311	-3.370	-4.030
4609	Blastemal	39	0	9.909	8.632	1.000	-4.301	-5.029	-5.379	-4.472	-4.170	-4.335	-3.148	-4.459
4612	Blastemal	72	0	8.519	6.919	1.000	-4.502	-4.984	-5.060	-4.755	-4.651	-4.366	-3.149	-3.859
4622	Stromal	63	0	7.467	6.984	1.000	-3.206	-5.036	-3.446	-4.653	-4.102	-4.627	-3.326	-4.568
4644	Mixed		0	7.326	6.876	1.585	-3.774	-5.650	-4.544	-4.632	-4.013	-4.851	-3.608	-4.230
4648	Regressive	55	0	7.516	6.939	1.000	-3.911	-5.552	-4.671	-4.236	-4.374	-4.367	-3.570	-4.383
4668	Diffuse Anaplastic	6	1	10.504	8.478	1.000	-3.633	-4.913	-4.433	-4.551	-4.476	-4.330	-3.104	-4.217
4674	Blastemal	75	0	10.475	8.658	1.000	-4.000	-5.461	-5.424	-4.334	-4.164	-4.823	-3.478	-4.206
4681	Mixed	61	0	8.352	7.026	1.585	-4.049	-4.847	-4.765	-4.235	-4.278	-4.235	-3.142	-3.782
4709	Epithelial	42	0	8.532	7.346	1.000	-3.914	-5.257	-5.771	-4.692	-4.832	-4.941	-3.593	-4.510
4714	Stromal	80	0	10.961	8.861	1.000	-3.948	-5.901	-5.219	-4.198	-4.596	-4.279	-3.415	-4.407
4735	Epithelial	10	1	10.076	8.045	1.585	-3.714	-4.629	-4.648	-4.251	-4.420	-4.416	-3.300	-3.670
4765	Epithelial	48	0	8.191	7.131	1.000	-3.771	-4.875	-3.870	-4.460	-3.677	-3.705	-3.354	-3.883
4766	Epithelial	64	0	9.110	7.448	1.000	-3.456	-4.664	-3.859	-3.861	-4.053	-4.470	-3.321	-3.984
4773	Mixed	56	0	8.578	7.203	1.000	-4.013	-5.136	-3.970	-4.014	-4.317	-4.174	-3.307	-4.143
4778	Mixed	88	0	8.440	7.210	1.000	-3.897	-3.585	-5.232	-4.676	-4.583	-4.571	-3.391	-4.385
4783	Stromal	59	0	7.416	6.933	1.000	-3.897	-5.796	-4.208	-4.319	-4.035	-4.285	-3.365	-4.533
4918	Mixed	71	0	10.065	8.124	1.585	-4.250	-4.762	-4.361	-4.596	-4.269	-4.360	-3.587	-3.826
5254	Mixed	62	1	10.160	7.981	1.000	-4.068	-5.293	-4.753	-5.000	-5.137	-4.278	-3.354	-4.190
5258	Mixed	77	0	10.060	7.796	1.000	-3.685	-4.711	-4.774	-4.214	-4.726	-3.978	-3.100	-3.984
5666	Diffuse Anaplastic	12	1	9.678	7.936	1.000	-3.892	-4.795	-5.396	-4.167	-5.653	-4.324	-3.134	-4.265

	methylation (M)	methylation (M)	methylation (M)	methylation (M)	methylation (M)	methylation (M)	methylation (M)	methylation (M)	methylation (M)	methylation (M)	methylation (M)	methylation (M)	methylation (M)	methylation (M)	methylation (M)
ICH ID	cg02204046	cg01010986	cg02905871	cg04251733	cg06520300	cg08884624	cg17360299	cg13131095	cg07468260	cg18032533	cg20431766	cg07083806	cg19623054	cg04609952	cg25074809
201	-3.539	-3.151	-4.080	-3.435	-4.139	-3.663	-5.210	-4.010	-3.248	-2.866	-1.631	-1.039	3.018	4.151	5.471
205	-3.808	-3.657	-3.496	-3.777	-3.937	-3.618	-4.949	-3.494	-3.324	-3.047	-3.345	-2.961	0.077	1.012	1.031
302	-4.076	-3.427	-4.049	-3.777	-4.821	-3.785	-4.355	-4.023	-3.410	-3.306	-3.671	-2.558	0.556	1.792	2.717
325	-3.509	-3.425	-3.071	-3.657	-3.647	-3.081	-3.851	-3.076	-3.110	-2.807	-1.835	-1.157	2.031	2.699	3.352
1159	-3.796	-3.626	-3.764	-3.642	-3.683	-3.345	-4.076	-3.654	-3.256	-3.463	-0.873	-0.581	3.814	4.487	5.796
1493	-3.611	-3.460	-3.674	-3.386	-3.388	-3.692	-3.283	-3.826	-3.294	-2.902	-2.695	-1.558	-0.121	1.263	1.099
1706	-4.090	-3.806	-3.721	-4.131	-5.151	-3.686	-4.269	-3.936	-3.350	-3.478	-3.002	-2.047	0.192	1.691	1.975
1784	-3.706	-3.448	-3.399	-3.089	-3.579	-3.624	-4.256	-4.035	-3.462	-3.235	-1.822	-1.113	2.898	3.292	4.139
2220	-4.083	-3.479	-3.367	-3.348	-3.047	-3.322	-3.891	-3.391	-3.136	-2.718	-2.662	-1.485	2.786	3.163	3.107
2261	-3.799	-3.986	-3.732	-4.143	-4.074	-3.775	-5.253	-4.068	-3.003	-3.070	-3.334	-3.508	-2.781	-1.783	-2.098
2382	-3.835	-3.298	-3.433	-3.329	-3.486	-3.558	-4.255	-3.488	-3.277	-2.918	-2.469	-1.731	0.336	1.709	1.078
2388	-3.967	-3.855	-3.485	-3.456	-3.167	-3.595	-3.206	-3.666	-3.433	-3.233	-1.363	-0.581	2.564	2.970	3.526
2557	-3.721	-3.452	-3.304	-4.040	-3.984	-3.504	-4.867	-3.769	-3.117	-2.824	-3.055	-2.539	-1.604	-0.630	-0.694
2594	-4.124	-3.695	-4.129	-4.298	-4.207	-3.707	-4.641	-3.944	-3.398	-3.175	-3.912	-3.478	-2.783	-1.911	-1.810
2652	-3.824	-3.729	-4.216	-3.748	-3.914	-3.765	-5.107	-3.702	-3.377	-3.435	-2.490	-1.707	2.489	3.425	4.052
2663	-3.809	-3.339	-3.724	-3.927	-4.099	-3.857	-4.649	-4.142	-3.195	-3.768	-1.151	-0.578	3.047	3.817	4.702
2664	-3.774	-3.461	-3.590	-3.323	-3.288	-3.555	-3.109	-3.816	-3.648	-2.567	-2.123	-1.263	2.408	3.015	3.787
2716	-3.816	-3.459	-3.492	-3.953	-3.761	-3.682	-3.662	-3.471	-3.168	-2.727	-2.884	-1.956	1.873	2.806	3.632
2717	-4.048	-3.444	-3.237	-3.600	-3.471	-3.383	-3.994	-3.438	-3.216	-2.995	-3.322	-2.900	-0.990	-0.093	0.174
2720	-3.783	-3.496	-3.307	-3.676	-3.149	-3.272	-4.618	-3.710	-3.211	-2.998	-3.539	-3.191	-1.067	-0.245	0.044
2734	-3.899	-3.647	-3.393	-3.618	-3.515	-3.714	-4.414	-3.588	-3.287	-2.687	-1.523	-1.012	2.782	3.559	4.240
2964	-4.168	-3.460	-3.400	-3.915	-3.357	-3.489	-3.973	-3.608	-3.287	-2.672	-3.148	-2.427	-0.317	1.055	1.019
2967	-2.473	-2.920	-2.522	-3.120	-3.211	-2.778	-4.806	-2.153	-2.337	-3.015	-2.675	-2.718	0.841	0.939	2.670
2969	-3.759	-3.754	-3.148	-3.872	-3.869	-4.047	-4.573	-3.547	-3.109	-2.815	-2.224	-1.470	0.758	1.803	2.170
2970	-3.902	-3.850	-3.329	-3.322	-3.873	-3.776	-3.475	-4.194	-3.521	-2.899	-1.623	-0.948	2.373	2.672	3.787
2987	-3.905	-3.289	-3.547	-3.742	-3.900	-3.575	-3.800	-3.402	-3.328	-2.655	-3.919	-4.054	-3.084	-2.855	-2.271
2990	-3.851	-3.688	-3.377	-3.845	-3.935	-3.315	-4.124	-3.579	-3.156	-2.395	-3.080	-2.740	-1.489	-0.850	-0.432
2994	-4.042	-3.279	-3.436	-3.676	-3.780	-3.737	-3.534	-3.346	-3.389	-2.856	-2.174	-1.547	0.803	2.221	1.648
3010	-3.720	-3.432	-3.438	-3.320	-3.601	-3.731	-3.661	-3.690	-3.222	-2.754	-3.560	-4.131	2.135	2.357	4.830
3020	-3.827	-3.517	-3.229	-3.815	-3.977	-3.453	-4.904	-3.619	-3.032	-2.657	-3.267	-2.759	-0.155	0.825	1.307
3136	-3.540	-3.505	-4.384	-3.202	-3.931	-3.560	-4.596	-3.803	-3.342	-3.028	-2.040	-1.360	1.995	2.314	2.758
3140	-3.938	-3.336	-3.325	-4.301	-3.392	-3.266	-4.457	-3.400	-3.011	-2.778	-1.544	-0.789	1.615	2.932	2.967
3143	-4.107	-3.597	-4.452	-2.999	-4.380	-3.710	-4.282	-4.018	-3.209	-3.791	-3.694	-3.692	-2.587	-1.617	-1.601
3145	-4.021	-3.683	-3.810	-3.494	-3.470	-3.483	-4.298	-3.842	-3.713	-2.870	-3.494	-3.699	-1.007	0.927	1.152
3148	-3.867	-3.234	-4.384	-3.057	-4.262	-3.844	-4.804	-3.612	-3.224	-3.850	-2.166	-1.787	2.332	2.862	3.726
3150	-3.579	-3.374	-3.118	-3.448	-3.713	-3.572	-4.389	-3.484	-3.258	-2.629	-1.010	-0.592	2.418	3.327	3.711
3477	-3.534	-3.496	-4.091	-4.227	-4.283	-4.098	-5.072	-3.691	-2.966	-3.148	-3.105	-2.917	0.078	0.898	1.420
3738	-3.691	-3.686	-3.947	-3.750	-4.090	-3.765	-4.804	-3.973	-3.273	-3.760	-1.126	-0.863	1.078	2.565	2.353
3745	-3.808	-3.185	-3.325	-3.824	-4.404	-3.275	-4.068	-3.799	-3.134	-4.538	-1.543	-1.058	2.691	2.948	3.048
3750	-3.606	-3.682	-3.487	-4.122	-3.863	-3.685	-3.894	-3.727	-2.802	-3.432	-1.948	-0.985	1.726	3.379	3.585
3751	-3.625	-3.401	-3.075	-3.570	-3.600	-3.599	-3.531	-3.502	-3.646	-2.554	-1.735	-1.517	1.465	2.612	2.967
3864	-3.457	-3.262	-3.101	-4.161	-3.950	-3.261	-4.774	-3.387	-2.983	-2.927	-3.569	-4.100	-0.154	0.933	1.347
3886	-3.867	-3.455	-4.637	-3.918	-4.329	-3.536	-5.764	-3.743	-3.011	-3.535	-3.500	-3.639	3.113	3.620	5.183
4083	-4.342	-3.235	-4.016	-3.780	-3.855	-3.528	-4.533	-3.908	-3.237	-3.425	-3.282	-2.146	-1.320	0.660	0.019
4092	-3.971	-3.607	-3.695	-3.513	-3.390	-2.966	-4.343	-3.632	-3.314	-2.796	-2.951	-1.917	2.261	3.279	3.139
4609	-3.633	-3.452	-4.246	-3.586	-3.724	-3.582	-4.774	-3.593	-2.981	-3.891	-3.745	-4.373	3.158	3.501	5.351
4612	-3.571	-3.132	-4.212	-3.206	-4.387	-3.527	-4.468	-3.859	-3.178	-3.100	-2.675	-2.219	1.417	2.532	3.627
4622	-3.733	-3.268	-3.431	-3.459	-3.247	-3.394	-2.977	-3.368	-3.291	-2.791	-1.998	-1.072	2.257	2.618	2.551
4644	-3.475	-4.044	-3.464	-3.685	-3.897	-3.515	-3.822	-3.781	-3.215	-2.657	-1.477	-0.908	2.500	3.303	3.940
4648	-3.708	-3.609	-3.307	-3.547	-3.582	-3.609	-3.754	-3.533	-3.357	-2.618	-2.416	-1.712	2.525	3.201	3.502
4668	-3.987	-3.414	-3.385	-3.807	-3.891	-3.765	-3.711	-3.793	-3.248	-2.302	-3.051	-2.921	1.298	2.720	3.281
4674	-3.658	-3.665	-3.709	-3.642	-3.787	-3.429	-4.405	-3.472	-3.120	-2.658	-3.419	-3.524	1.954	2.860	5.267
4681	-3.435	-3.409	-3.451	-3.565	-3.623	-3.391	-4.140	-3.500	-3.085	-2.676	-2.188	-1.441	1.945	2.799	3.411
4709	-3.865	-3.570	-4.093	-3.703	-4.257	-3.780	-4.485	-3.902	-3.353	-3.288	-2.283	-1.408	1.243	2.952	2.680
4714	-3.384	-3.458	-4.854	-3.327	-3.535	-3.762	-5.682	-3.758	-3.667	-3.240	-1.834	-0.837	2.248	3.511	2.926
4735	-3.879	-3.378	-3.003	-3.879	-3.538	-3.590	-3.953	-3.727	-3.128	-1.954	-1.772	-0.938	0.459	1.810	1.974
4765	-3.878	-3.506	-3.606	-3.047	-2.884	-2.905	-2.913	-3.543	-3.794	-1.516	-2.549	-2.301	2.312	2.820	4.213
4766	-3.906	-3.176	-3.353	-3.266	-3.619	-3.516	-3.368	-3.521	-3.356	-3.273	-2.324	-1.888	2.608	3.453	4.872
4773	-3.850	-3.232	-3.376	-3.937	-3.505	-3.526	-3.433	-3.622	-3.168	-2.324	-2.987	-2.364	0.638	1.714	1.982
4778	-3.867	-3.143	-3.653	-3.533	-3.664	-3.900	-4.395	-3.501	-3.245	-2.712	-2.099	-1.128	2.169	3.008	2.955
4783	-3.750	-3.572	-3.258	-3.571	-3.956	-3.616	-3.778	-3.564	-3.155	-3.427	-2.387	-1.526	1.536	2.450	2.086
4918	-3.938	-3.457	-3.475	-4.026	-3.436	-3.305	-3.842	-3.669	-3.160	-2.941	-2.171	-1.303	0.319	1.644	1.775
5254	-3.572	-3.111	-3.497	-4.115	-3.602	-3.461	-3.813	-3.651	-3.019	-3.472	-2.591	-2.046	0.950	2.090	2.034
5258	-3.531	-2.814	-3.038	-3.785	-3.626	-3.287	-3.875	-3.343	-2.802	-3.099	-1.948	-1.488	0.429	1.631	1.587
5666	-3.546	-2.795	-3.172	-3.684	-3.499	-3.042	-4.023	-3.318	-2.967	-2.729	-3.338	-3.181	2.737	3.968	5.220

Supplementary Figure 1

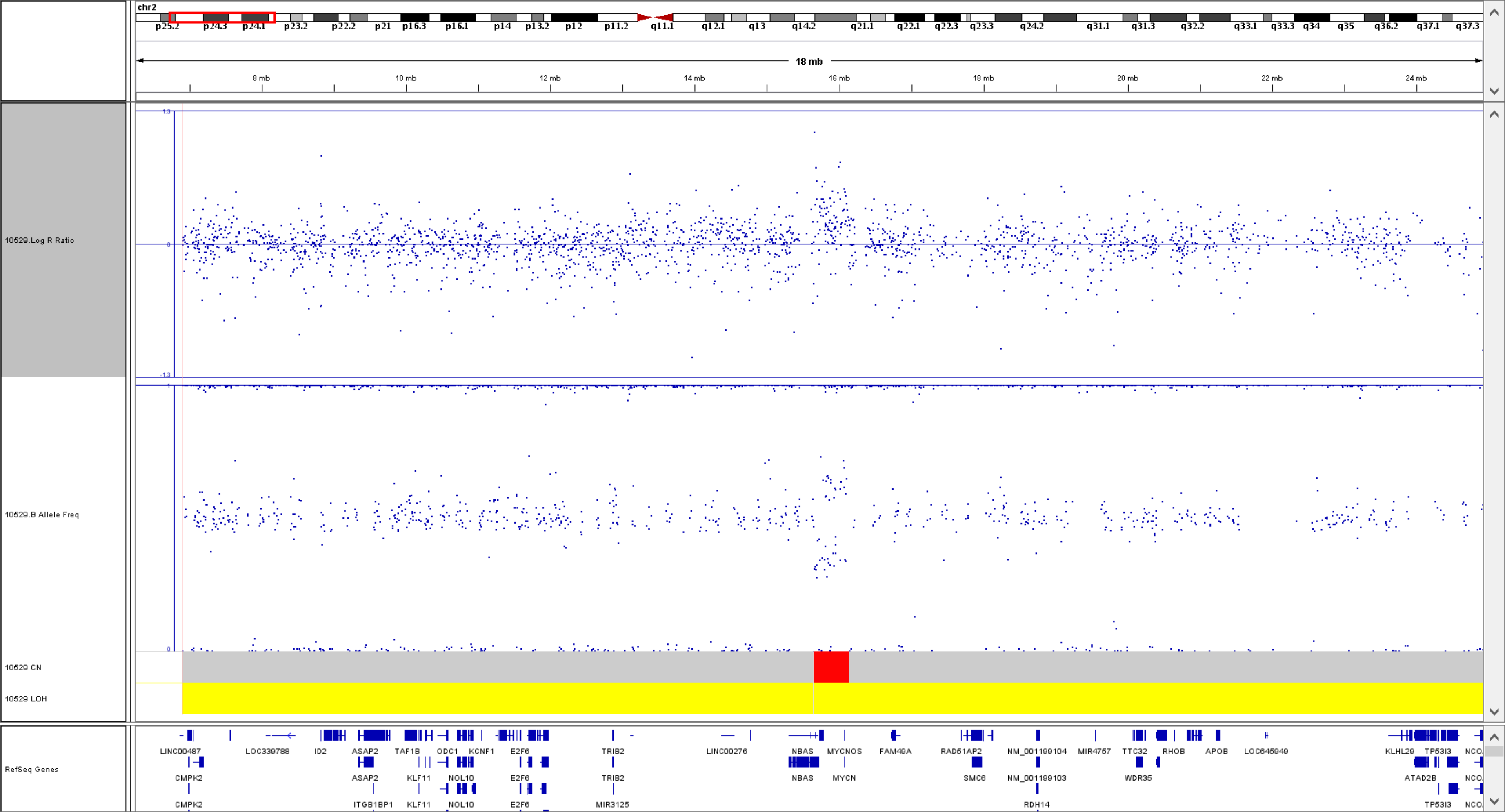

chr2
p25.2 p24.3 p24.1 p23.2 p22.2 p21 p16.3 p16.1 p14 p13.2 p12 p11.2 q11.1 q12.1 q13 q14.2 q21.1 q22.1 q22.3 q23.3 q24.2 q31.1 q31.3 q32.2 q33.1 q33.3 q34 q35 q36.2 q37.1 q37.3
18 mb
8 mb 10 mb 12 mb 14 mb 16 mb 18 mb 20 mb 22 mb 24 mb
10529.Log R Ratio
10529.B Allele Freq
10529 CN
10529 LOH
RefSeq Genes
LINC00487 LOC339788 ID2 ASAP2 TAF1B ODC1 KCNF1 E2F6 TRIB2 LINC00276 NBAS MYCNOS FAM49A RAD51AP2 NM_001199104 MIR4757 TTC32 RHOB APOB LOC645949 KLHL29 TP53I3
CMPK2 ASAP2 KLF11 NOL10 E2F6 TRIB2 NBAS MYCN SMC6 NM_001199103 WDR35 ATAD2B
CMPK2 ITGB1BP1 KLF11 NOL10 E2F6 MIR3125 RDH14 TP53I3

Supplementary Figure 2

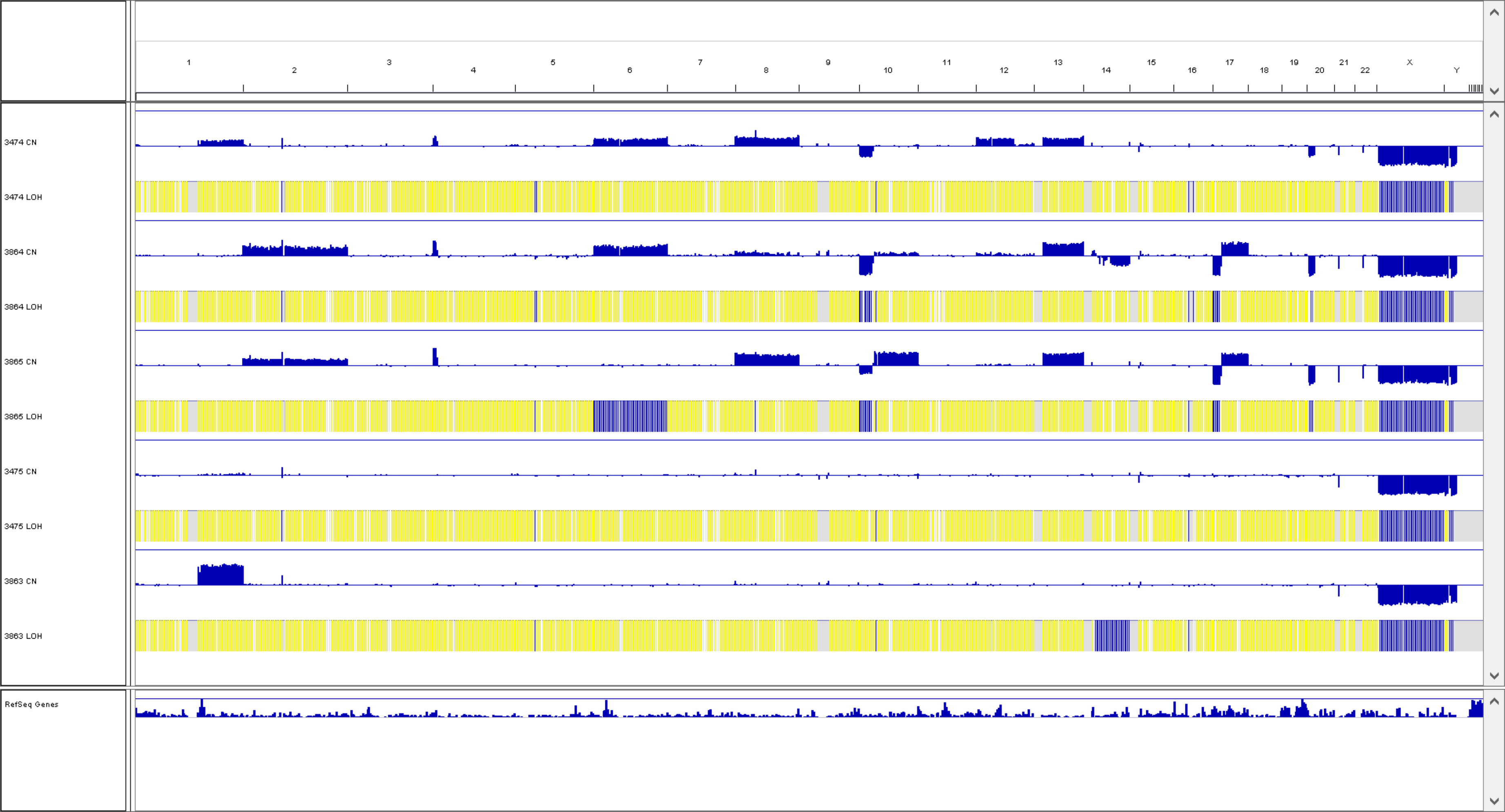
1
2
3
4
5
6
7
8
9
10
11
12
13
14
15
16
17
18
19
20
21
22
X
Y
3474 CN
3474 LOH
3864 CN
3864 LOH
3865 CN
3865 LOH
3475 CN
3475 LOH
3863 CN
3863 LOH
RefSeq Genes

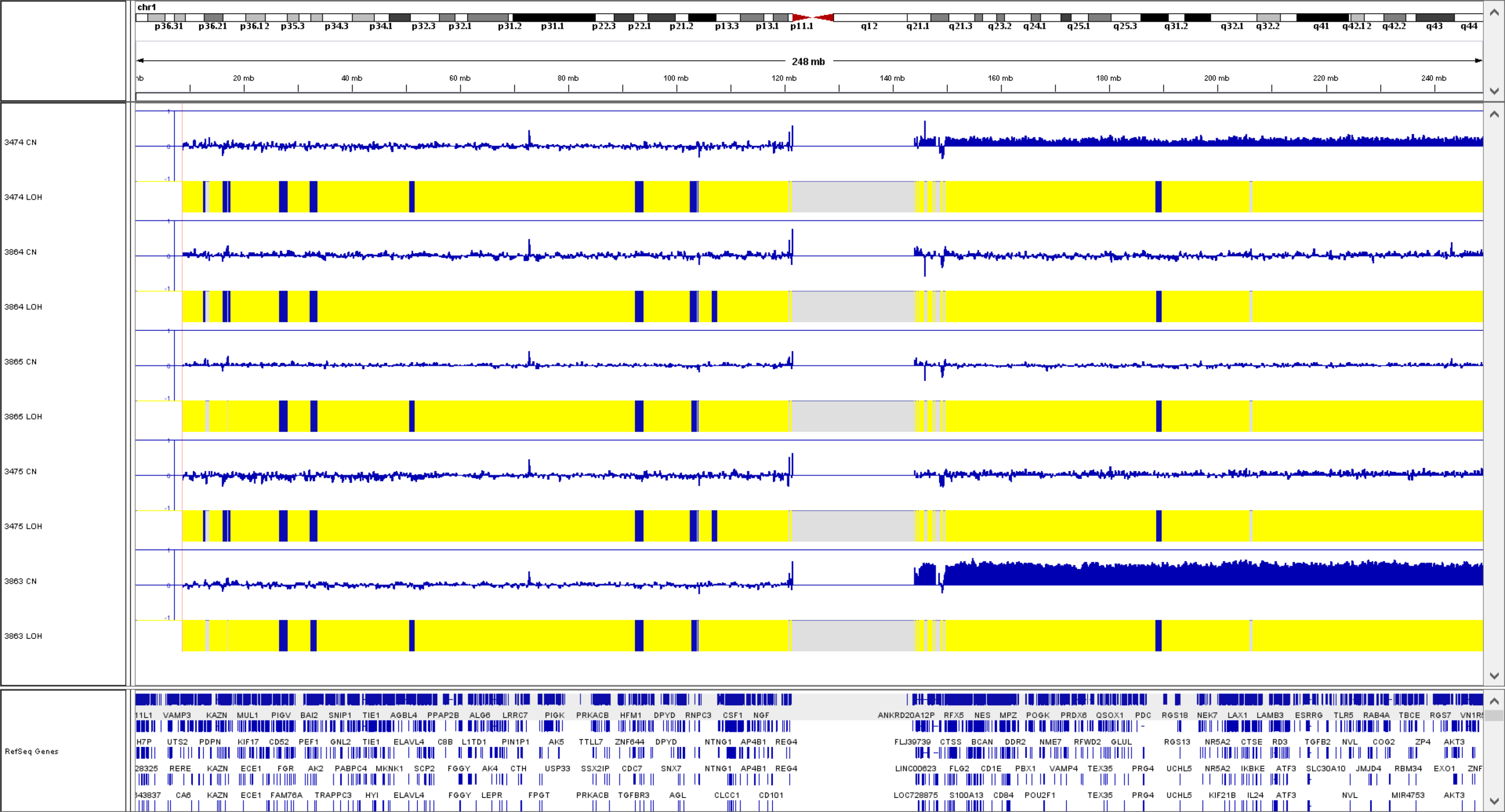

chr1
p36.31 p36.21 p36.12 p35.3 p34.3 p34.1 p32.3 p32.1 p31.2 p31.1 p22.3 p22.1 p21.2 p13.3 p13.1 p11.1 q12 q21.1 q21.3 q23.2 q24.1 q25.1 q25.3 q31.2 q32.1 q32.2 q41 q42.12 q42.2 q43 q44
248 mb
20 mb
40 mb
60 mb
80 mb
100 mb
120 mb
140 mb
160 mb
180 mb
200 mb
220 mb
240 mb
3474 CN
3474 LOH
3864 CN
3864 LOH
3865 CN
3865 LOH
3475 CN
3475 LOH
3863 CN
3863 LOH
RefSeq Genes

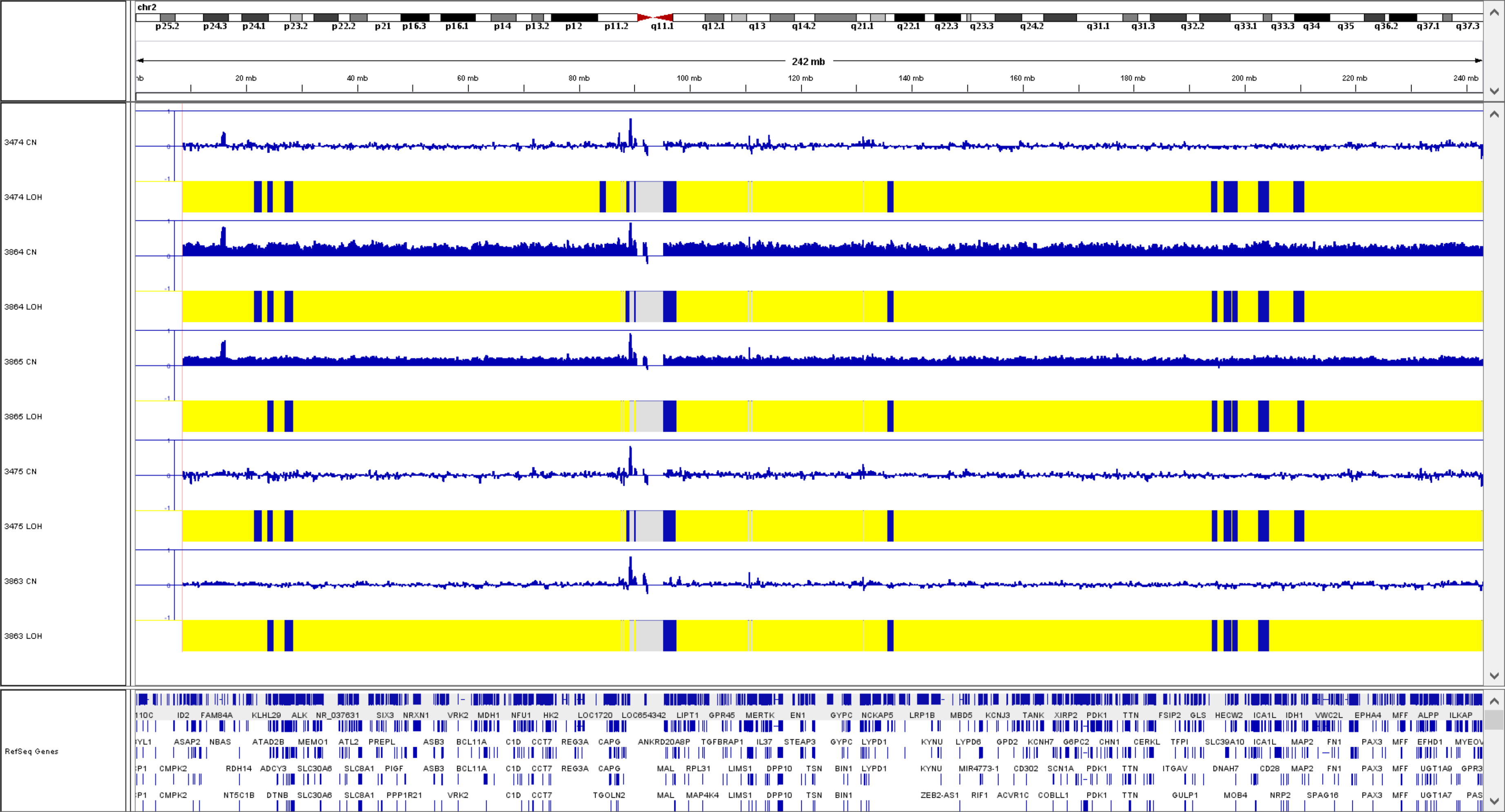
chr2
p25.2 p24.3 p24.1 p23.2 p22.2 p21 p16.3 p16.1 p14 p13.2 p12 p11.2 q11.1 q12.1 q13 q14.2 q21.1 q22.1 q22.3 q23.3 q24.2 q31.1 q31.3 q32.2 q33.1 q33.3 q34 q35 q36.2 q37.1 q37.3
242 mb
20 mb 40 mb 60 mb 80 mb 100 mb 120 mb 140 mb 160 mb 180 mb 200 mb 220 mb 240 mb
3474 CN
3474 LOH
3864 CN
3864 LOH
3865 CN
3865 LOH
3475 CN
3475 LOH
3863 CN
3863 LOH
RefSeq Genes
ID2 FAM84A KLHL29 ALK NR_037631 SIX3 NRXN1 VRK2 MDH1 NFU1 HK2 LOC1720 LOC654342 LIPT1 GPR45 MERTK EN1 GYPC NCKAP5 LRP1B MBD5 KCNJ3 TANK XIRP2 PDK1 TTN FSIP2 GLS HECW2 ICA1L IDH1 VWC2L EPHA4 MFF ALPP ILKAP
ASAP2 NBAS ATAD2B MEMO1 ATL2 PREPL ASB3 BCL11A C1D CCT7 REG3A CAPG ANKRD20A8P TGFBRAP1 IL37 STEAP3 GYPC LYPD1 KYNU LYPD6 GPD2 KCNH7 G6PC2 CHN1 CERKL TFPI SLC39A10 ICA1L MAP2 PAX3 MFF EFHD1
CMPK2 RDH14 ADCY3 SLC30A6 SLC8A1 PIGF ASB3 BCL11A C1D CCT7 REG3A CAPG MAL RPL31 LIMS1 DPP10 TSN BIN1 LYPD1 KYNU MIR4773-1 CD302 SCN1A PDK1 TTN ITGAV DNAH7 CD28 MAP2 FN1 PAX3 MFF UGT1A9
CMPK2 NT5C1B DTNB SLC30A6 SLC8A1 PPP1R21 VRK2 C1D CCT7 TGOLN2 MAL MAP4K4 LIMS1 DPP10 TSN BIN1 ZEB2-AS1 RIF1 ACVR1C COBLL1 PDK1 TTN GULP1 MOB4 NRP2 SPAG16 PAX3 MFF UGT1A7

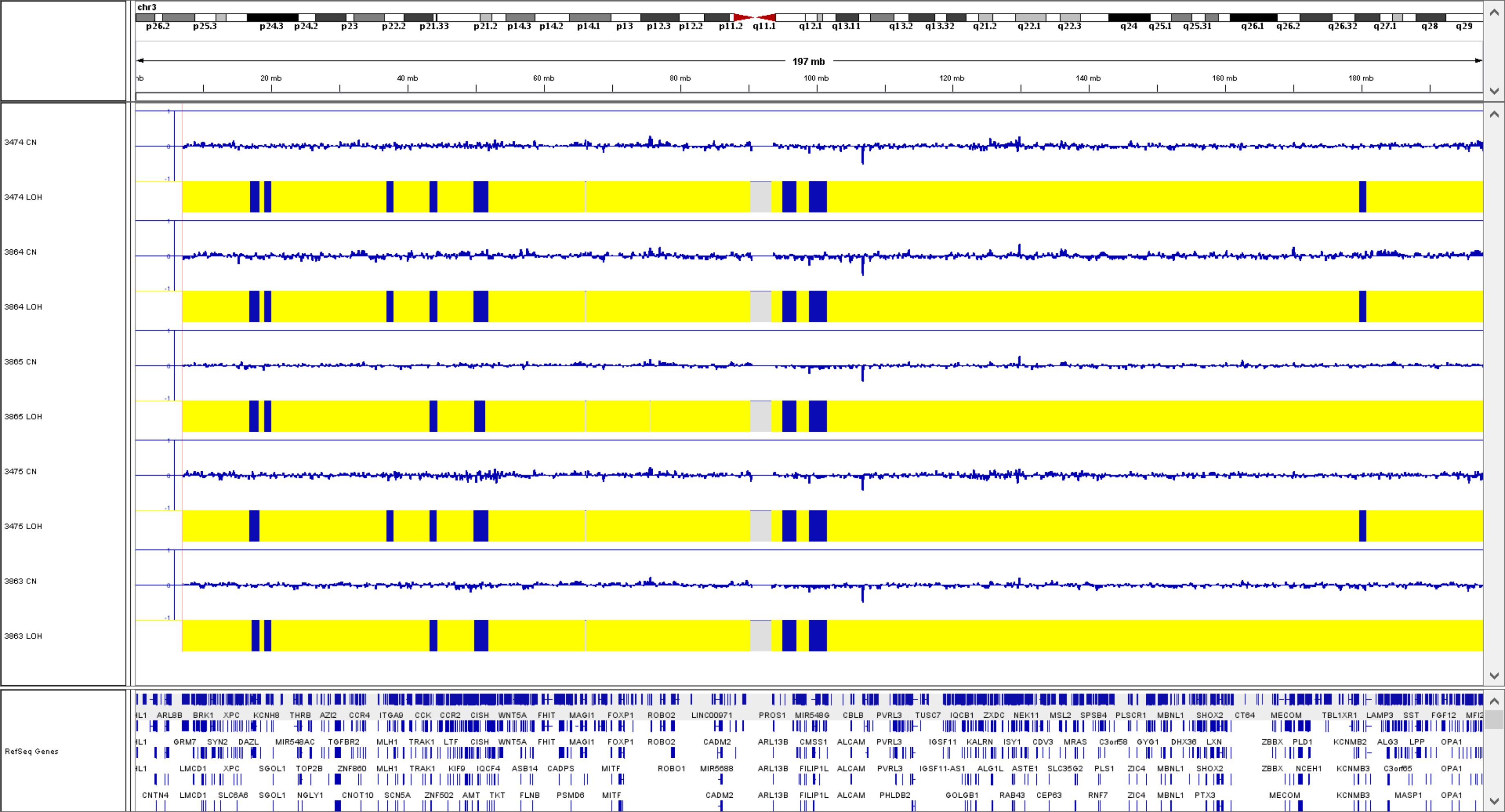

chr3
p26.2 p25.3 p24.3 p24.2 p23 p22.2 p21.33 p21.2 p14.3 p14.2 p14.1 p13 p12.3 p12.2 p11.2 q11.1 q12.1 q13.11 q13.2 q13.32 q21.2 q22.1 q22.3 q24 q25.1 q25.31 q26.1 q26.2 q26.32 q27.1 q28 q29
197 mb
20 mb
40 mb
60 mb
80 mb
100 mb
120 mb
140 mb
160 mb
180 mb
3474 CN
3474 LOH
3864 CN
3864 LOH
3865 CN
3865 LOH
3475 CN
3475 LOH
3863 CN
3863 LOH
RefSeq Genes

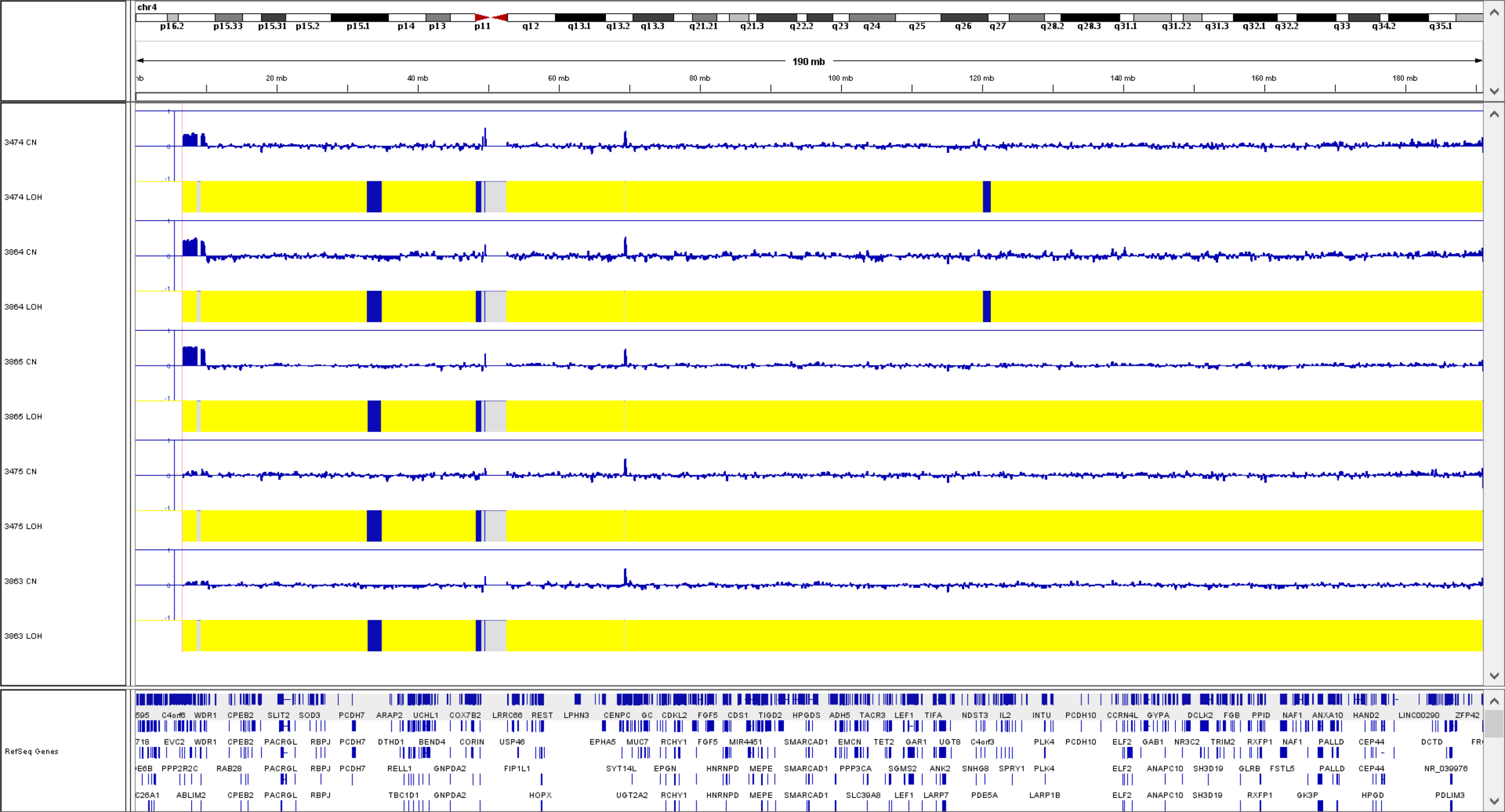
chr4
p16.2 p15.33 p15.31 p15.2 p15.1 p14 p13 p11 q12 q13.1 q13.2 q13.3 q21.21 q21.3 q22.2 q23 q24 q25 q26 q27 q28.2 q28.3 q31.1 q31.22 q31.3 q32.1 q32.2 q33 q34.2 q35.1
190 mb
20 mb 40 mb 60 mb 80 mb 100 mb 120 mb 140 mb 160 mb 180 mb
3474 CN
3474 LOH
3864 CN
3864 LOH
3865 CN
3865 LOH
3475 CN
3475 LOH
3863 CN
3863 LOH
RefSeq Genes
C4orf6 WDR1 CPEB2 SLIT2 SOD3 PCDH7 ARAP2 UCHL1 COX7B2 LRRC66 REST LPHN3 CENPC GC CDKL2 FGF5 CDS1 TIGD2 HPGDS ADH5 TACR3 LEF1 TIFA NDST3 IL2 INTU PCDH10 CCRN4L GYPA DCLK2 FGB PPID NAF1 ANXA10 HAND2 LINC00290 ZFP42
EVC2 WDR1 CPEB2 PACRGL RBPJ PCDH7 DTHD1 BEND4 CORIN USP46 EPHA5 MUC7 RCHY1 FGF5 MIR4451 SMARCAD1 EMCN TET2 GAR1 UGT8 C4orf3 PLK4 PCDH10 ELF2 GAB1 NR3C2 TRIM2 RXFP1 NAF1 PALLD CEP44 DCTD
PPP2R2C RAB28 PACRGL RBPJ PCDH7 RELL1 GNPDA2 FIP1L1 SYT14L EPGN HNRNPD MEPE SMARCAD1 PPP3CA SGMS2 ANK2 SNHG8 SPRY1 PLK4 ELF2 ANAPC10 SH3D19 GLRB FSTL5 PALLD CEP44 NR_039976
ABLIM2 CPEB2 PACRGL RBPJ TBC1D1 GNPDA2 HOPX UGT2A2 RCHY1 HNRNPD MEPE SMARCAD1 SLC39A8 LEF1 LARP7 PDE5A LARP1B ELF2 ANAPC10 SH3D19 RXFP1 GK3P HPGD PDLIM3

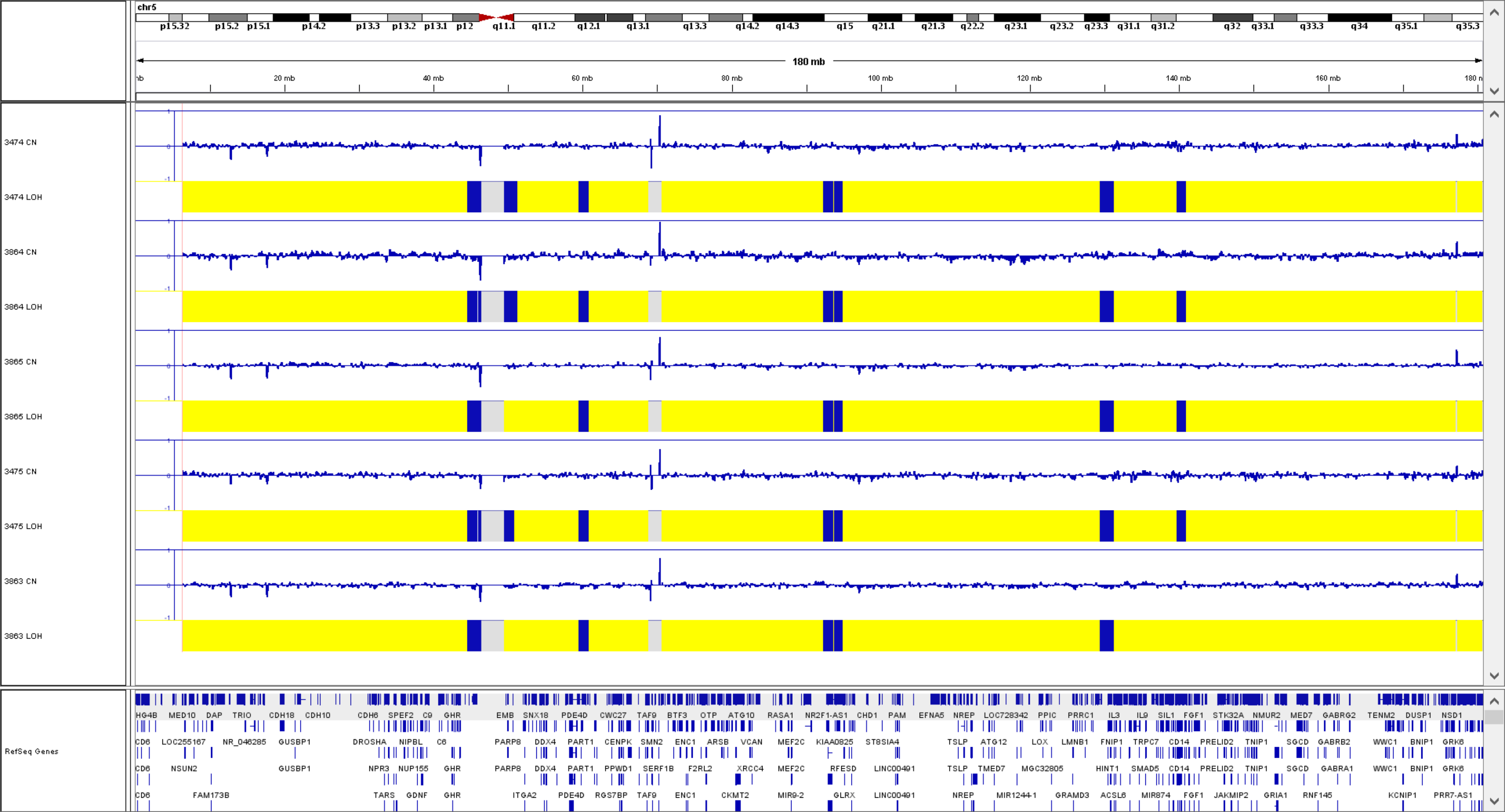
chr5
p15.32 p15.2 p15.1 p14.2 p13.3 p13.2 p13.1 p12 q11.1 q11.2 q12.1 q13.1 q13.3 q14.2 q14.3 q15 q21.1 q21.3 q22.2 q23.1 q23.2 q23.3 q31.1 q31.2 q32 q33.1 q33.3 q34 q35.1 q35.3
180 mb
20 mb
40 mb
60 mb
80 mb
100 mb
120 mb
140 mb
160 mb
3474 CN
3474 LOH
3864 CN
3864 LOH
3865 CN
3865 LOH
3475 CN
3475 LOH
3863 CN
3863 LOH
RefSeq Genes
MED10 DAP TRIO CDH18 CDH10 CDH6 SPEF2 C9 GHR EMB SNX18 PDE4D CWC27 TAF9 BTF3 OTP ATG10 RASA1 NR2F1-AS1 CHD1 PAM EFNA5 NREP LOC728342 PPIC PRRC1 IL3 IL9 SIL1 FGF1 STK32A NMUR2 MED7 GABRG2 TENM2 DUSP1 NSD1
CD6 LOC255167 NR_046285 GUSBP1 DROSHA NIPBL C6 PARP8 DDX4 PART1 CENPK SMN2 ENC1 ARSB VCAN MEF2C KIAA0825 ST8SIA4 TSLP ATG12 LOX LMNB1 FNIP1 TRPC7 CD14 PRELID2 TNIP1 SGCD GABRB2 WWC1 BNIP1 GRK6
CD6 NSUN2 GUSBP1 NPR3 NUP155 GHR PARP8 DDX4 PART1 PPWD1 SERF1B F2RL2 XRCC4 MEF2C RFESD LINC00491 TSLP TMED7 MGC32805 HINT1 SMAD5 CD14 PRELID2 TNIP1 SGCD GABRA1 WWC1 BNIP1 GRK6
CD6 FAM173B TARS GDNF GHR ITGA2 PDE4D RGS7BP TAF9 ENC1 CKMT2 MIR9-2 GLRX LINC00491 NREP MIR1244-1 GRAMD3 ACSL6 MIR874 FGF1 JAKMIP2 GRIA1 RNF145 KCNIP1 PRR7-AS1

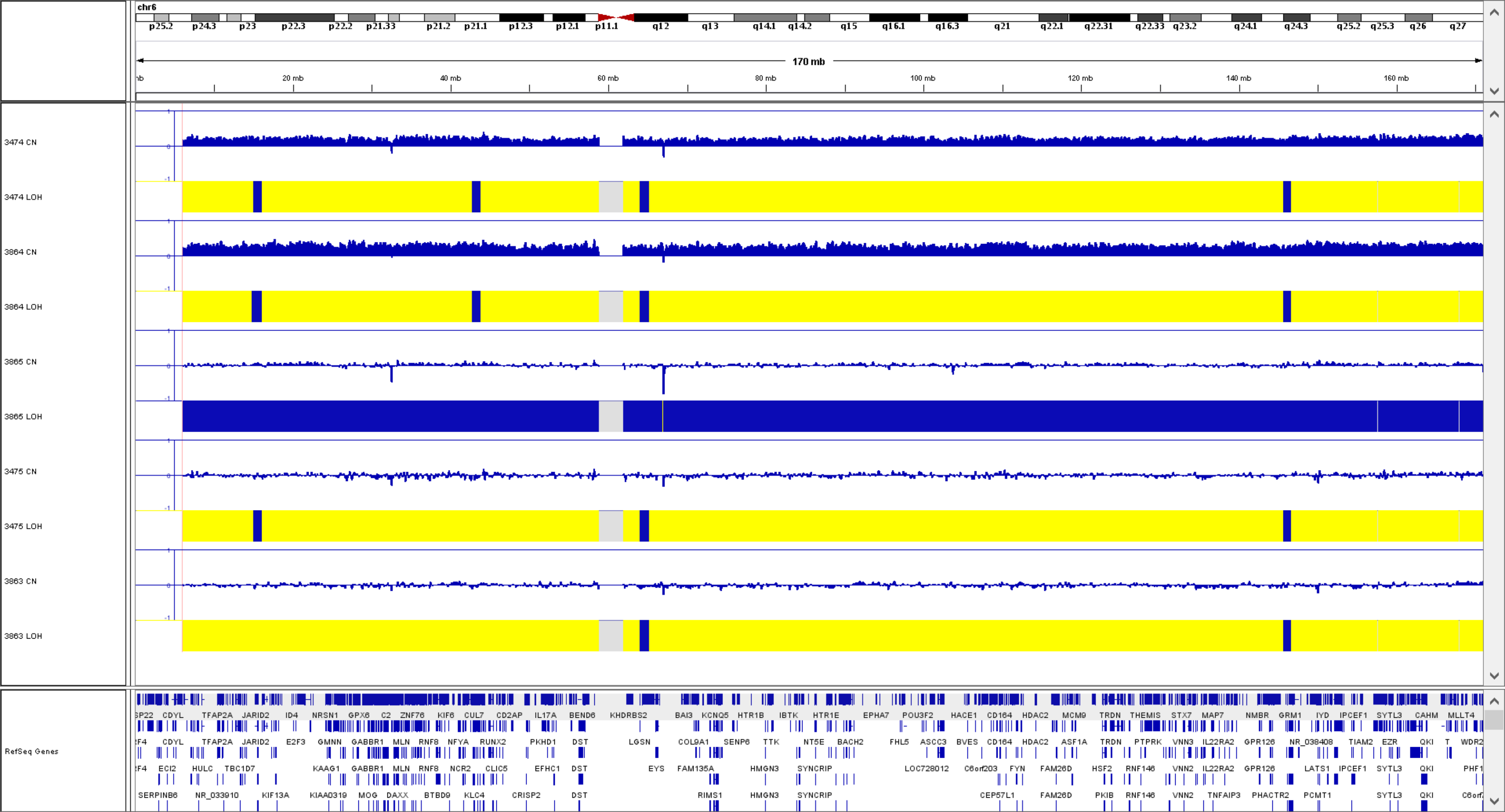
chr6
p25.2
p24.3
p23
p22.3
p22.2
p21.33
p21.2
p21.1
p12.3
p12.1
p11.1
q12
q13
q14.1
q14.2
q15
q16.1
q16.3
q21
q22.1
q22.31
q22.33
q23.2
q24.1
q24.3
q25.2
q25.3
q26
q27
170 mb
20 mb
40 mb
60 mb
80 mb
100 mb
120 mb
140 mb
160 mb
3474 CN
3474 LOH
3864 CN
3864 LOH
3865 CN
3865 LOH
3475 CN
3475 LOH
3863 CN
3863 LOH
RefSeq Genes

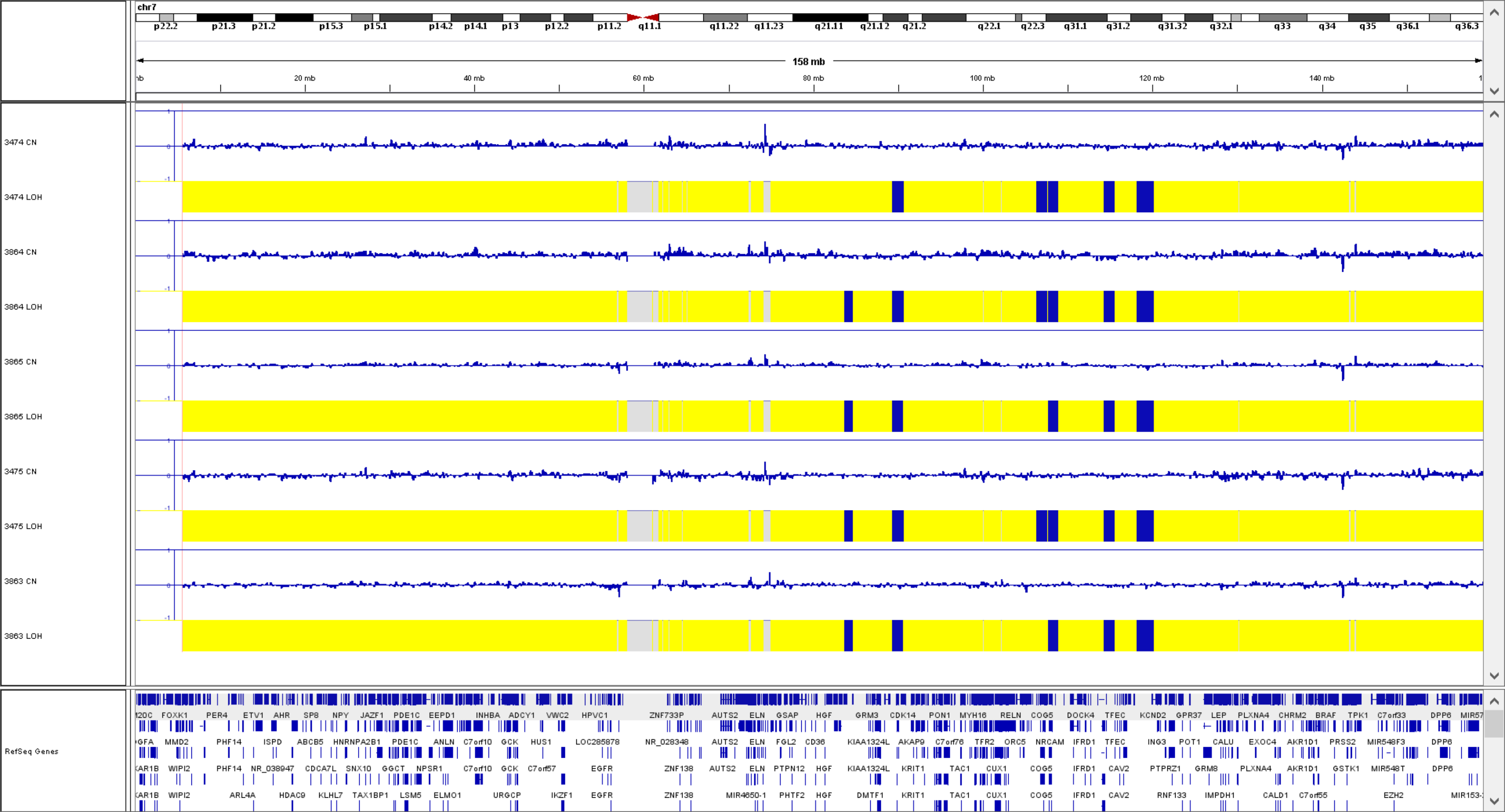
chr7
p22.2 p21.3 p21.2 p15.3 p15.1 p14.2 p14.1 p13 p12.2 p11.2 q11.1 q11.22 q11.23 q21.11 q21.12 q21.2 q22.1 q22.3 q31.1 q31.2 q31.32 q32.1 q33 q34 q35 q36.1 q36.3
158 mb
20 mb
40 mb
60 mb
80 mb
100 mb
120 mb
140 mb
3474 CN
3474 LOH
3864 CN
3864 LOH
3865 CN
3865 LOH
3475 CN
3475 LOH
3863 CN
3863 LOH
RefSeq Genes

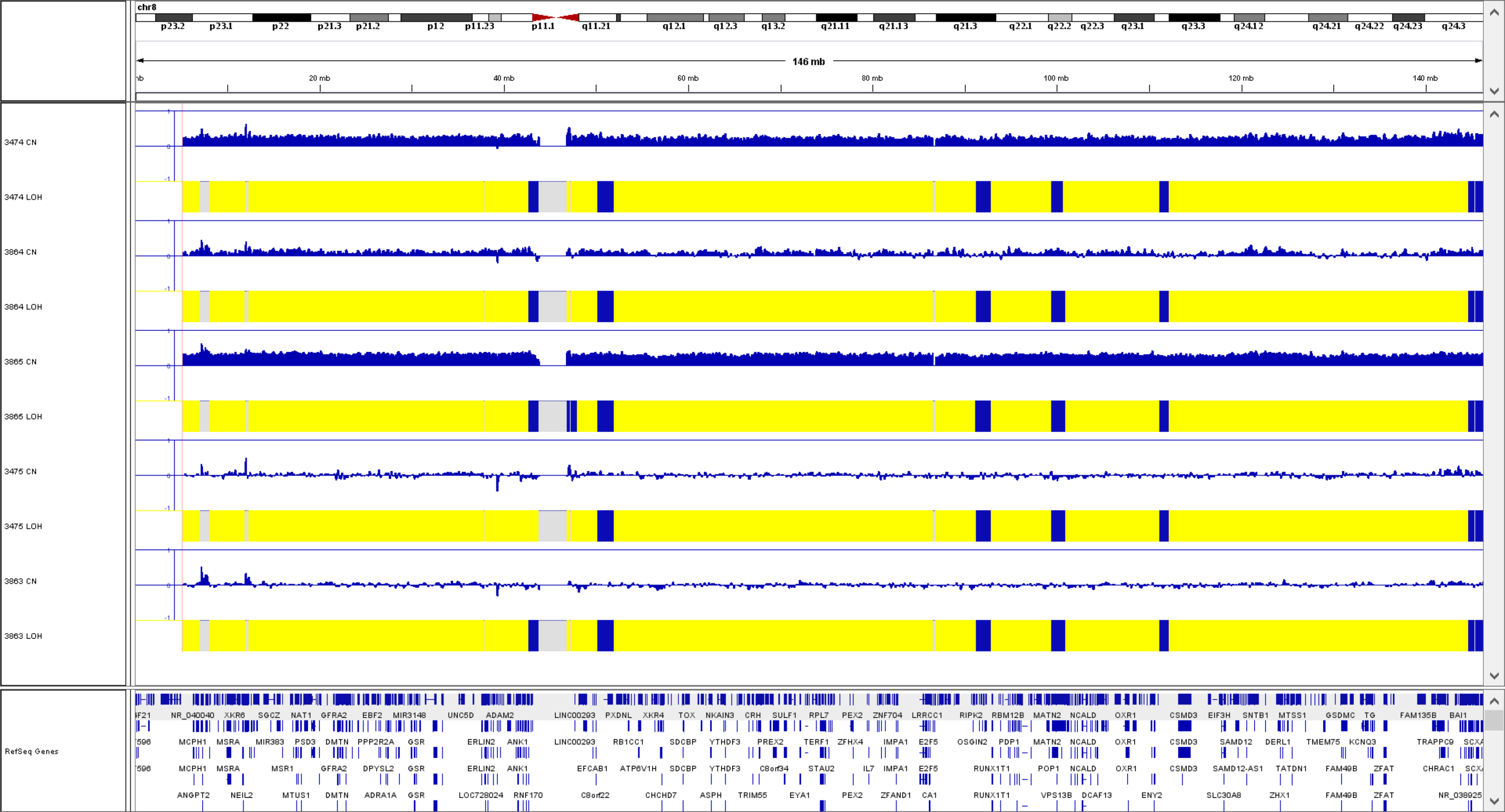
chr8
p23.2 p23.1 p22 p21.3 p21.2 p12 p11.23 p11.1 q11.21 q12.1 q12.3 q13.2 q21.11 q21.13 q21.3 q22.1 q22.2 q22.3 q23.1 q23.3 q24.12 q24.21 q24.22 q24.23 q24.3
146 mb
20 mb
40 mb
60 mb
80 mb
100 mb
120 mb
140 mb
3474 CN
3474 LOH
3864 CN
3864 LOH
3865 CN
3865 LOH
3475 CN
3475 LOH
3863 CN
3863 LOH
RefSeq Genes
NR_040040 XKR6 SGCZ NAT1 GFRA2 EBF2 MIR3148 UNC5D ADAM2 LINC00293 PXDNL XKR4 TOX NKAIN3 CRH SULF1 RPL7 PEX2 ZNF704 LRRCC1 RIPK2 RBM12B MATN2 NCALD OXR1 CSMD3 EIF3H SNTB1 MTSS1 GSDMC TG FAM135B BAI1
MCPH1 MSRA MIR383 PSD3 DMTN PPP2R2A GSR ERLIN2 ANK1 LINC00293 RB1CC1 SDCBP YTHDF3 PREX2 TERF1 ZFHX4 IMPA1 E2F5 OSGIN2 PDP1 MATN2 NCALD OXR1 CSMD3 SAMD12 DERL1 TMEM75 KCNQ3 TRAPPC9
MCPH1 MSRA MSR1 GFRA2 DPYSL2 GSR ERLIN2 ANK1 EFCAB1 ATP6V1H SDCBP YTHDF3 C8orf34 STAU2 IL7 IMPA1 E2F5 RUNX1T1 POP1 NCALD OXR1 CSMD3 SAMD12-AS1 TATDN1 FAM49B ZFAT CHRAC1
ANGPT2 NEIL2 MTUS1 DMTN ADRA1A GSR LOC728024 RNF170 C8orf22 CHCHD7 ASPH TRIM55 EYA1 PEX2 ZFAND1 CA1 RUNX1T1 VPS13B DCAF13 ENY2 SLC30A8 ZHX1 FAM49B ZFAT NR_038925

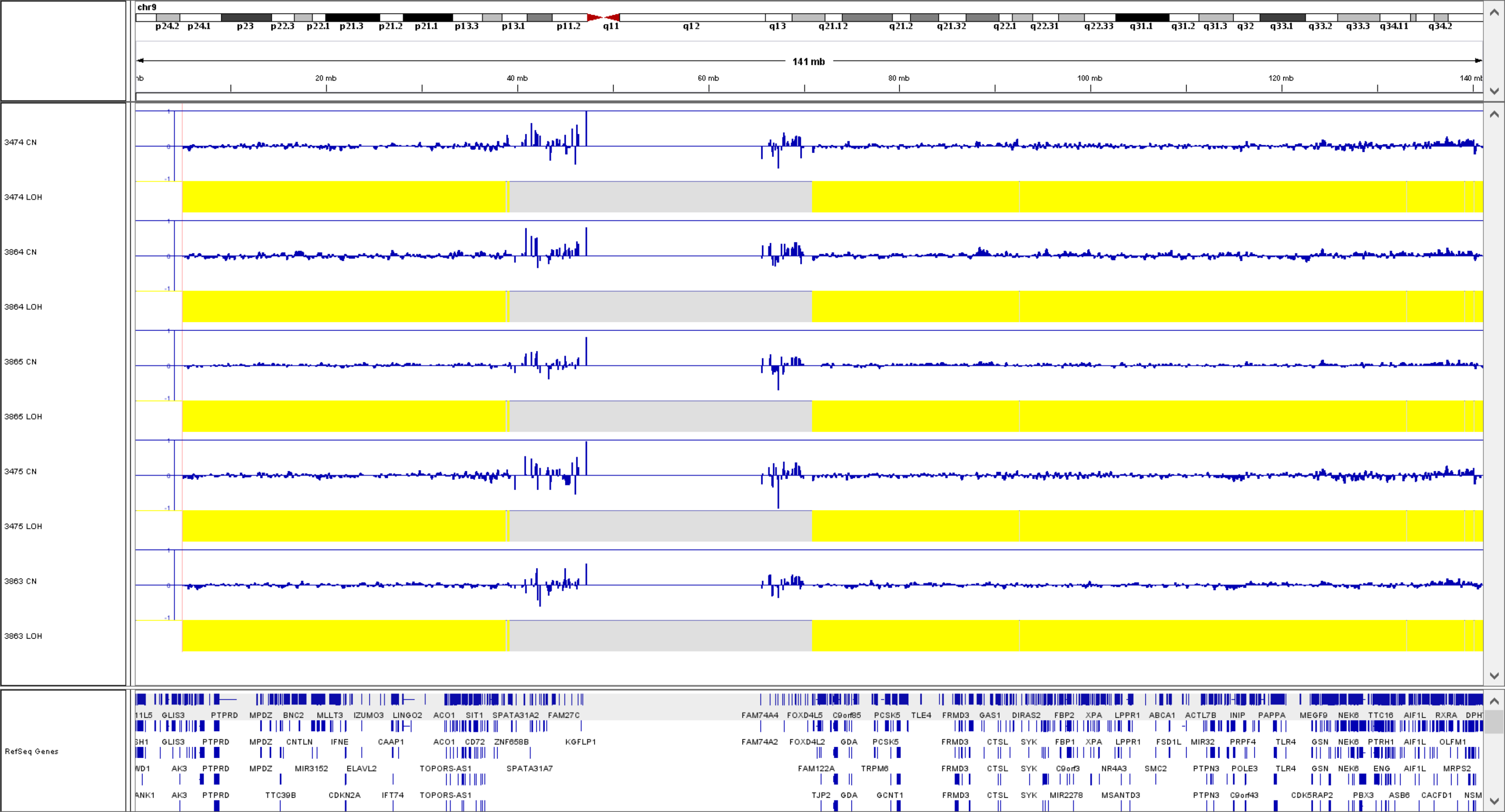

chr9
p24.2 p24.1 p23 p22.3 p22.1 p21.3 p21.2 p21.1 p13.3 p13.1 p11.2 q11 q12 q13 q21.12 q21.2 q21.32 q22.1 q22.31 q22.33 q31.1 q31.2 q31.3 q32 q33.1 q33.2 q33.3 q34.11 q34.2
141 mb
20 mb
40 mb
60 mb
80 mb
100 mb
120 mb
3474 CN
3474 LOH
3864 CN
3864 LOH
3865 CN
3865 LOH
3475 CN
3475 LOH
3863 CN
3863 LOH
RefSeq Genes
GLIS3 PTPRD MPDZ BNC2 MLLT3 IZUMO3 LINGO2 ACO1 SIT1 SPATA31A2 FAM27C FAM74A4 FOXD4L5 C9orf85 PCSK5 TLE4 FRMD3 GAS1 DIRAS2 FBP2 XPA LPPR1 ABCA1 ACTL7B INIP PAPPA MEGF9 NEK6 TTC16 AIF1L RXRA
GLIS3 PTPRD MPDZ CNTLN IFNE CAAP1 ACO1 CD72 ZNF658B KGFLP1 FAM74A2 FOXD4L2 GDA PCSK5 FRMD3 CTSL SYK FBP1 XPA LPPR1 FSD1L MIR32 PRPF4 TLR4 GSN NEK6 PTRH1 AIF1L OLFM1
AK3 PTPRD MPDZ MIR3152 ELAVL2 TOPORS-AS1 SPATA31A7 FAM122A TRPM6 FRMD3 CTSL SYK C9orf3 NR4A3 SMC2 PTPN3 POLE3 TLR4 GSN NEK6 ENG AIF1L MRPS2
AK3 PTPRD TTC39B CDKN2A IFT74 TOPORS-AS1 TJP2 GDA GCNT1 FRMD3 CTSL SYK MIR2278 MSANTD3 PTPN3 C9orf43 CDK5RAP2 PBX3 ASB6 CACFD1

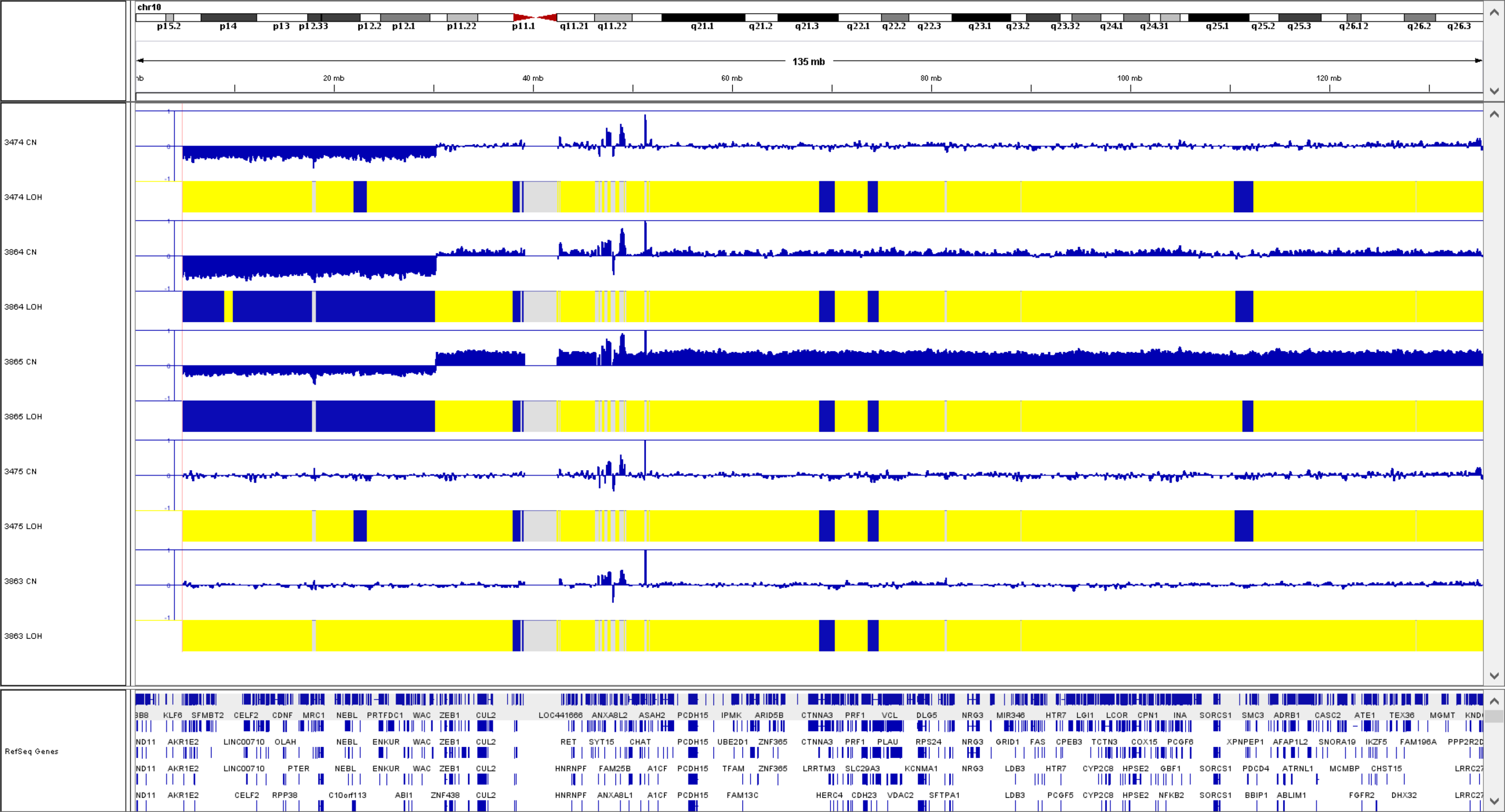
chr10
p15.2 p14 p13 p12.33 p12.2 p12.1 p11.22 p11.1 q11.21 q11.22 q21.1 q21.2 q21.3 q22.1 q22.2 q22.3 q23.1 q23.2 q23.32 q24.1 q24.31 q25.1 q25.2 q25.3 q26.12 q26.2 q26.3
135 mb
20 mb
40 mb
60 mb
80 mb
100 mb
120 mb
3474 CN
3474 LOH
3864 CN
3864 LOH
3865 CN
3865 LOH
3475 CN
3475 LOH
3863 CN
3863 LOH
RefSeq Genes
KLF6 SFMBT2 CELF2 CDNF MRC1 NEBL PRTFDC1 WAC ZEB1 CUL2 LOC441666 ANXA8L2 ASAH2 PCDH15 IPMK ARID5B CTNNA3 PRF1 VCL DLG5 NRG3 MIR346 HTR7 LGI1 LCOR CPN1 INA SORCS1 SMC3 ADRB1 CASC2 ATE1 TEX36 MGMT
AKR1E2 LINC00710 OLAH NEBL ENKUR WAC ZEB1 CUL2 RET SYT15 CHAT PCDH15 UBE2D1 ZNF365 CTNNA3 PRF1 PLAU RPS24 NRG3 GRID1 FAS CPEB3 TCTN3 COX15 PCGF6 XPNPEP1 AFAP1L2 SNORA19 IKZF5 FAM196A
AKR1E2 LINC00710 PTER NEBL ENKUR WAC ZEB1 CUL2 HNRNPF FAM25B A1CF PCDH15 TFAM ZNF365 LRRTM3 SLC29A3 KCNMA1 NRG3 LDB3 HTR7 CYP2C8 HPSE2 GBF1 SORCS1 PDCD4 ATRNL1 MCMBP CHST15
AKR1E2 CELF2 RPP38 C10orf113 ABI1 ZNF438 CUL2 HNRNPF ANXA8L1 A1CF PCDH15 FAM13C HERC4 CDH23 VDAC2 SFTPA1 LDB3 PCGF5 CYP2C8 HPSE2 NFKB2 SORCS1 BBIP1 ABLIM1 FGFR2 DHX32

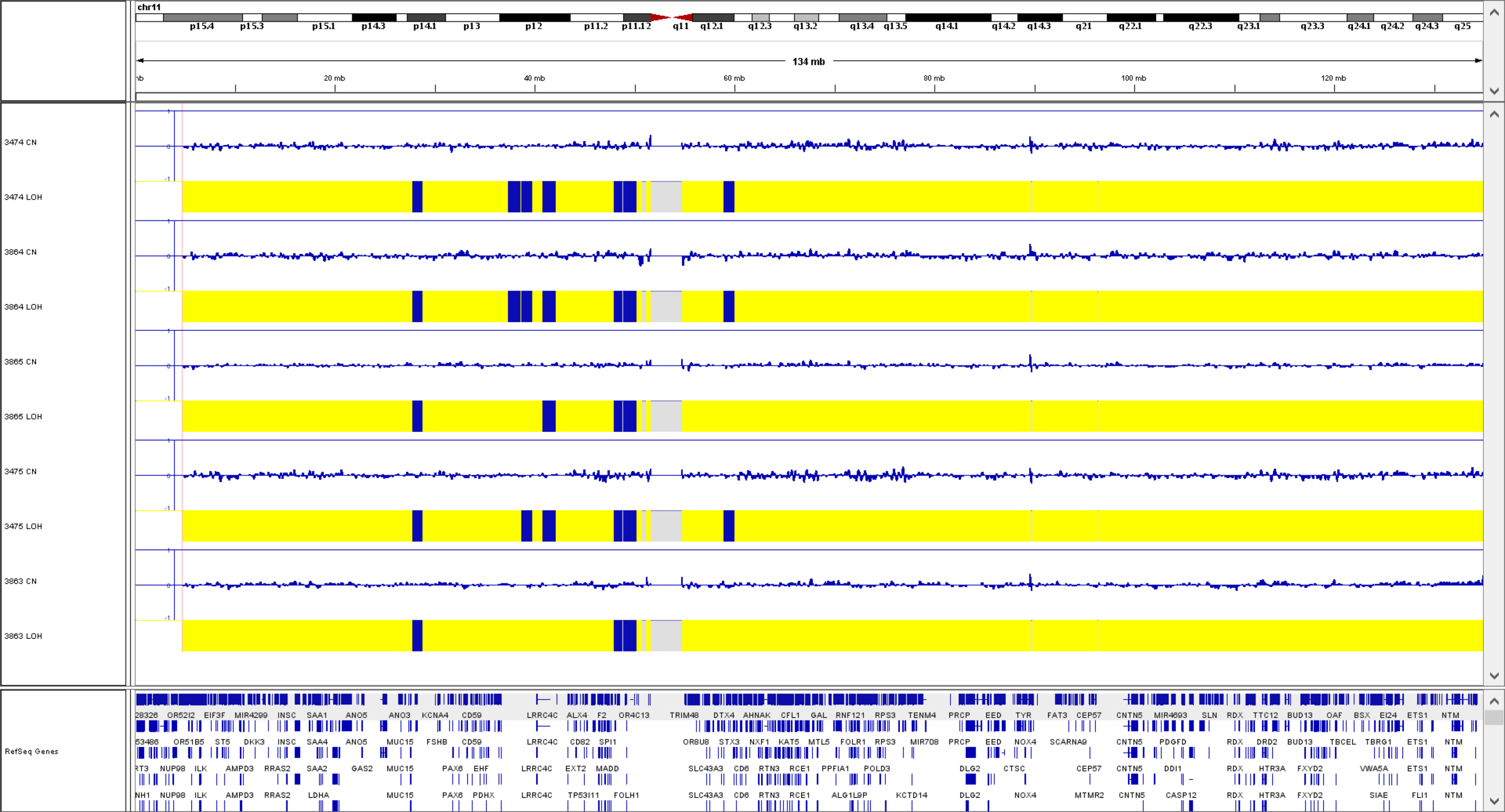
chr11
p15.4
p15.3
p15.1
p14.3
p14.1
p13
p12
p11.2
p11.12
q11
q12.1
q12.3
q13.2
q13.4
q13.5
q14.1
q14.2
q14.3
q21
q22.1
q22.3
q23.1
q23.3
q24.1
q24.2
q24.3
q25
134 mb
20 mb
40 mb
60 mb
80 mb
100 mb
120 mb
3474 CN
3474 LOH
3864 CN
3864 LOH
3865 CN
3865 LOH
3475 CN
3475 LOH
3863 CN
3863 LOH
RefSeq Genes

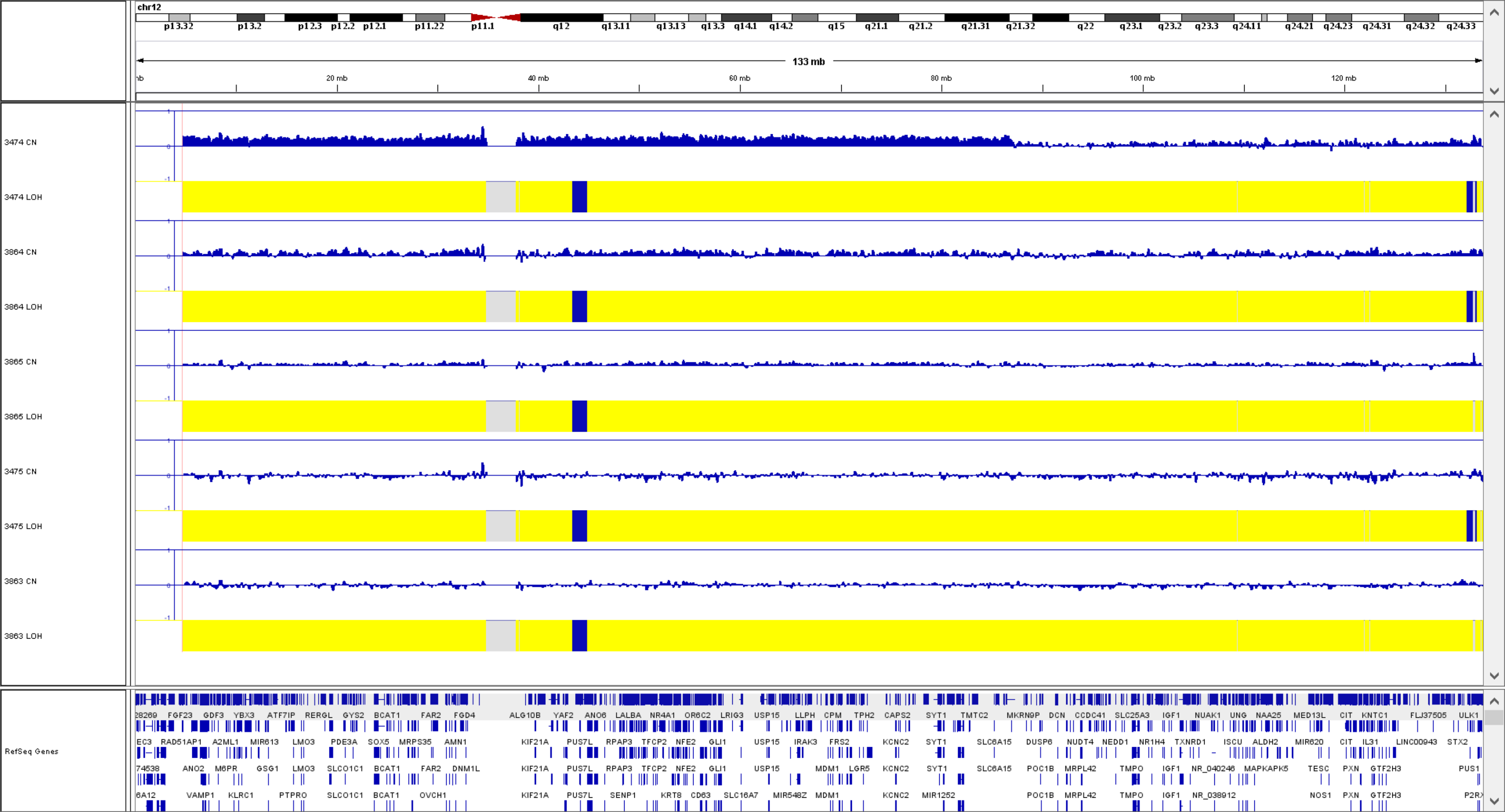
chr12
p13.32 p13.2 p12.3 p12.2 p12.1 p11.22 p11.1 q12 q13.11 q13.13 q13.3 q14.1 q14.2 q15 q21.1 q21.2 q21.31 q21.32 q22 q23.1 q23.2 q23.3 q24.11 q24.21 q24.23 q24.31 q24.32 q24.33
133 mb
20 mb
40 mb
60 mb
80 mb
100 mb
120 mb
3474 CN
3474 LOH
3864 CN
3864 LOH
3865 CN
3865 LOH
3475 CN
3475 LOH
3863 CN
3863 LOH
RefSeq Genes

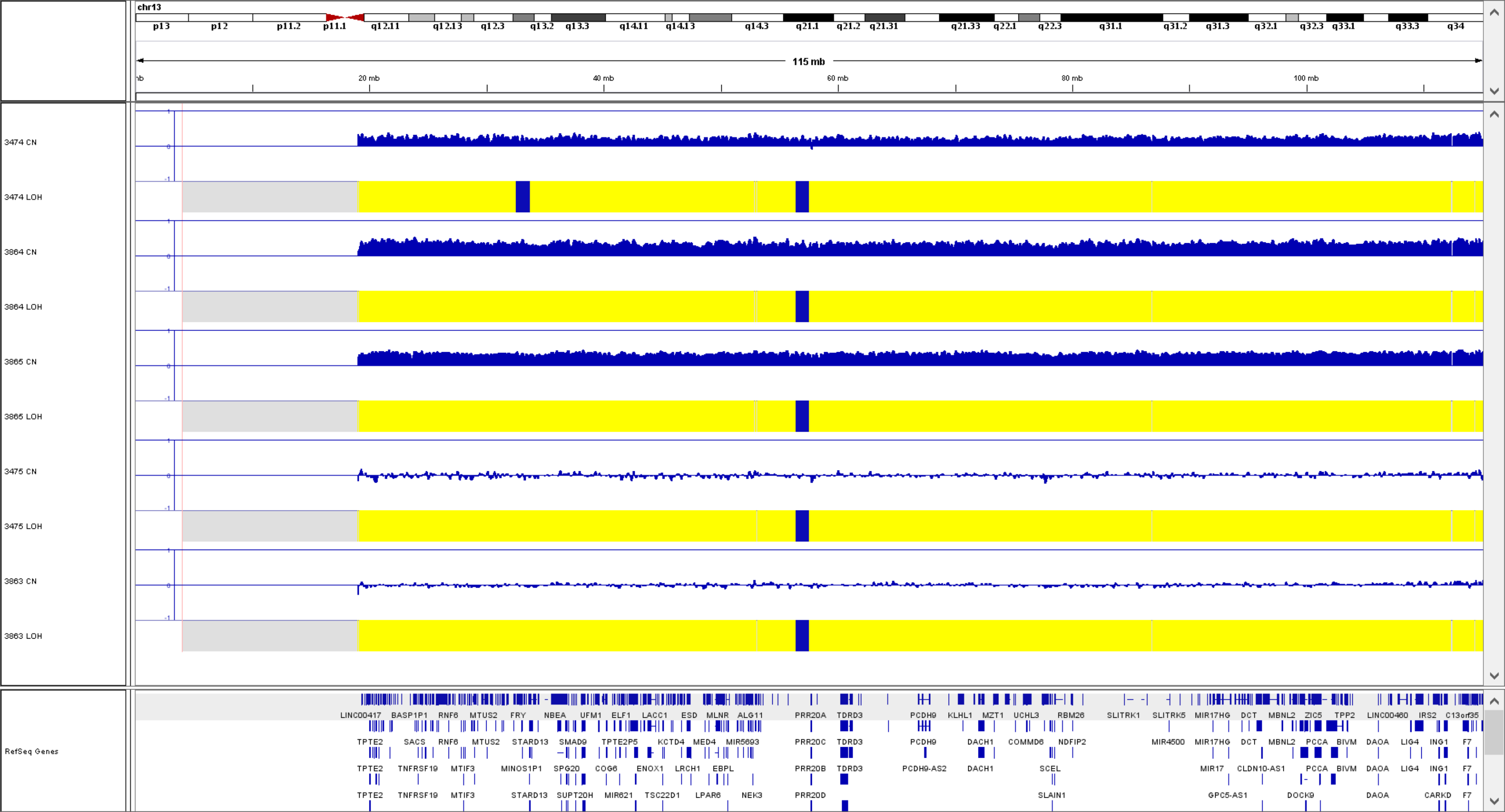

chr13
p13
p12
p11.2
p11.1
q12.11
q12.13
q12.3
q13.2
q13.3
q14.11
q14.13
q14.3
q21.1
q21.2
q21.31
q21.33
q22.1
q22.3
q31.1
q31.2
q31.3
q32.1
q32.3
q33.1
q33.3
q34
115 mb
20 mb
40 mb
60 mb
80 mb
100 mb
3474 CN
3474 LOH
3864 CN
3864 LOH
3865 CN
3865 LOH
3475 CN
3475 LOH
3863 CN
3863 LOH
RefSeq Genes

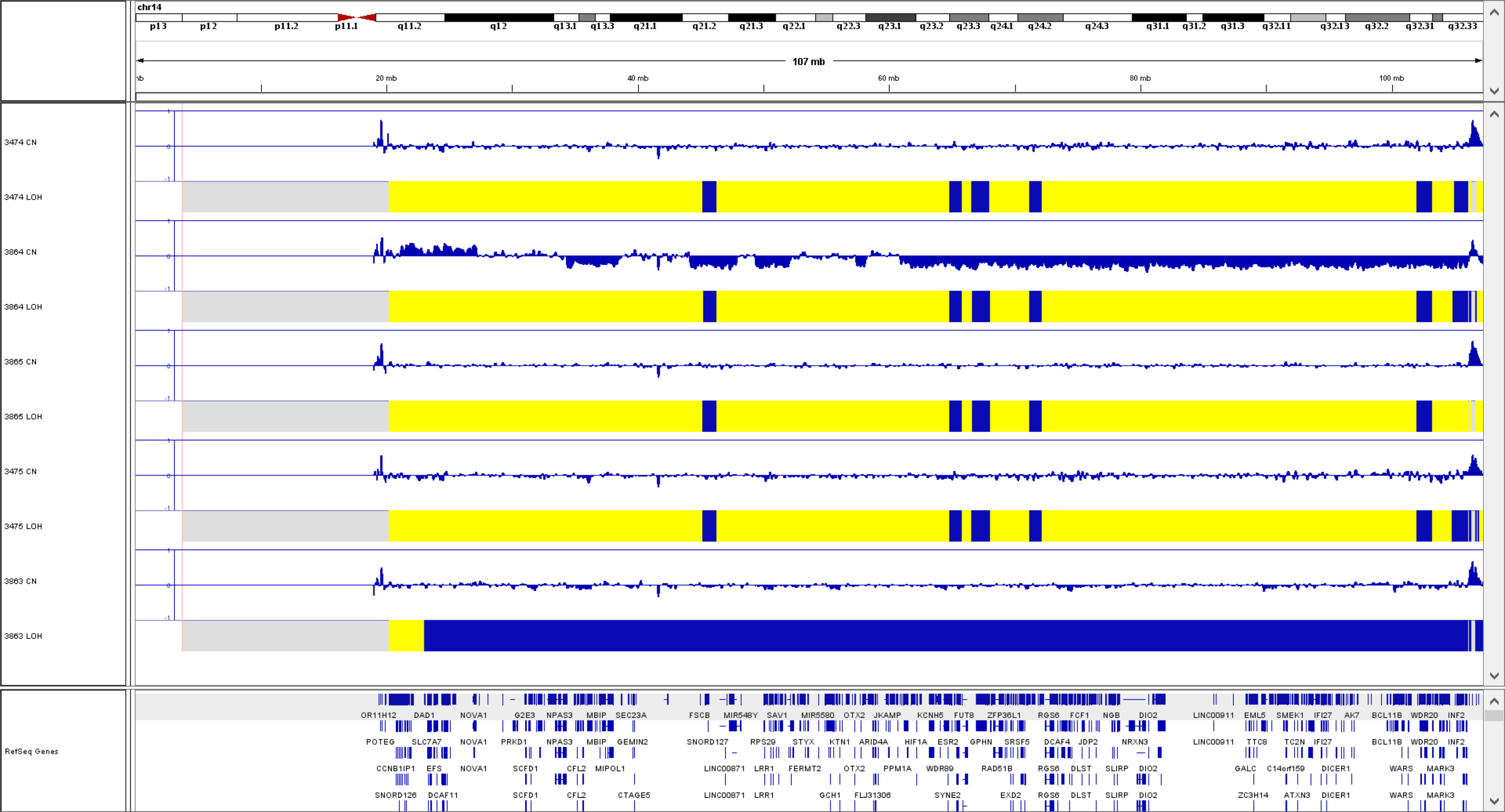

chr14
p13 p12 p11.2 p11.1 q11.2 q12 q13.1 q13.3 q21.1 q21.2 q21.3 q22.1 q22.3 q23.1 q23.2 q23.3 q24.1 q24.2 q24.3 q31.1 q31.2 q31.3 q32.11 q32.13 q32.2 q32.31 q32.33
107 mb
20 mb
40 mb
60 mb
80 mb
100 mb
3474 CN
3474 LOH
3864 CN
3864 LOH
3865 CN
3865 LOH
3475 CN
3475 LOH
3863 CN
3863 LOH
RefSeq Genes
OR11H12 DAD1 NOVA1 G2E3 NPAS3 MBIP SEC23A FSCB MIR548Y SAV1 MIR5580 OTX2 JKAMP KCNH5 FUT8 ZFP36L1 RGS6 FCF1 NGB DIO2 LINC00911 EML5 SMEK1 IFI27 AK7 BCL11B WDR20 INF2
POTEG SLC7A7 NOVA1 PRKD1 NPAS3 MBIP GEMIN2 SNORD127 RPS29 STYX KTN1 ARID4A HIF1A ESR2 GPHN SRSF5 DCAF4 JDP2 NRXN3 LINC00911 TTC8 TC2N IFI27 BCL11B WDR20 INF2
CCNB1IP1 EFS NOVA1 SCFD1 CFL2 MIPOL1 LINC00871 LRR1 FERMT2 OTX2 PPM1A WDR89 RAD51B RGS6 DLST SLIRP DIO2 GALC C14orf159 DICER1 WARS MARK3
SNORD126 DCAF11 SCFD1 CFL2 CTAGE5 LINC00871 LRR1 GCH1 FLJ31306 SYNE2 EXD2 RGS6 DLST SLIRP DIO2 ZC3H14 ATXN3 DICER1 WARS MARK3

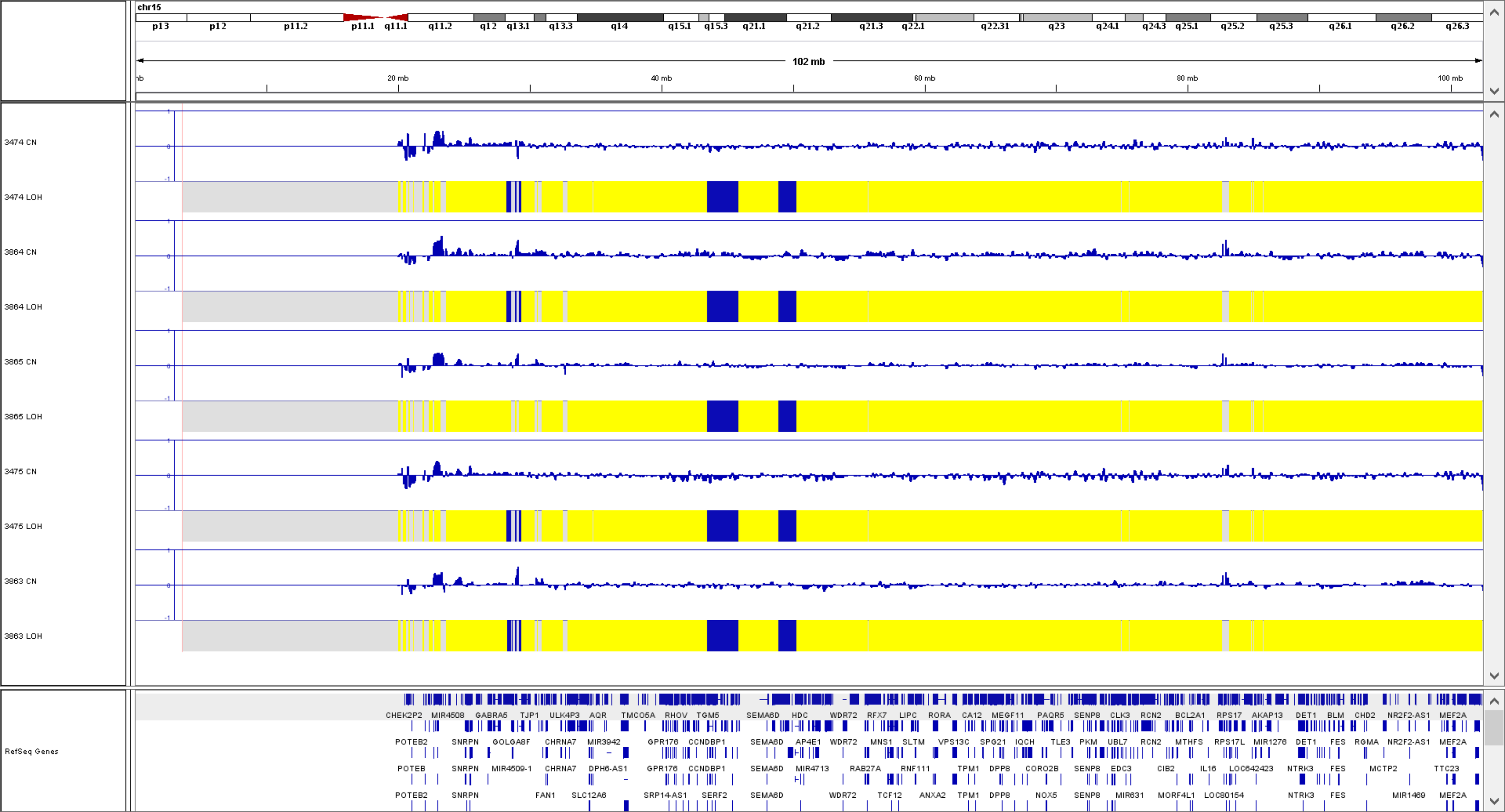
chr15
p13 p12 p11.2 p11.1 q11.1 q11.2 q12 q13.1 q13.3 q14 q15.1 q15.3 q21.1 q21.2 q21.3 q22.1 q22.31 q23 q24.1 q24.3 q25.1 q25.2 q25.3 q26.1 q26.2 q26.3
102 mb
20 mb
40 mb
60 mb
80 mb
100 mb
3474 CN
3474 LOH
3864 CN
3864 LOH
3865 CN
3865 LOH
3475 CN
3475 LOH
3863 CN
3863 LOH
RefSeq Genes
CHEK2P2 MIR4508 GABRA5 TJP1 ULK4P3 AQR TMCO5A RHOV TGM5 SEMA6D HDC WDR72 RFX7 LIPC RORA CA12 MEGF11 PAQR5 SENP8 CLK3 RCN2 BCL2A1 RPS17 AKAP13 DET1 BLM CHD2 NR2F2-AS1 MEF2A
POTEB2 SNRPN GOLGA8F CHRNA7 MIR3942 GPR176 CCNDBP1 SEMA6D AP4E1 WDR72 MNS1 SLTM VPS13C SPG21 IQCH TLE3 PKM UBL7 RCN2 MTHFS RPS17L MIR1276 DET1 FES RGMA NR2F2-AS1 MEF2A
POTEB SNRPN MIR4509-1 CHRNA7 DPH6-AS1 GPR176 CCNDBP1 SEMA6D MIR4713 RAB27A RNF111 TPM1 DPP8 CORO2B SENP8 EDC3 CIB2 IL16 LOC642423 NTRK3 FES MCTP2 TTC23
POTEB2 SNRPN FAN1 SLC12A6 SRP14-AS1 SERF2 SEMA6D WDR72 TCF12 ANXA2 TPM1 DPP8 NOX5 SENP8 MIR631 MORF4L1 LOC80154 NTRK3 FES MIR1469 MEF2A

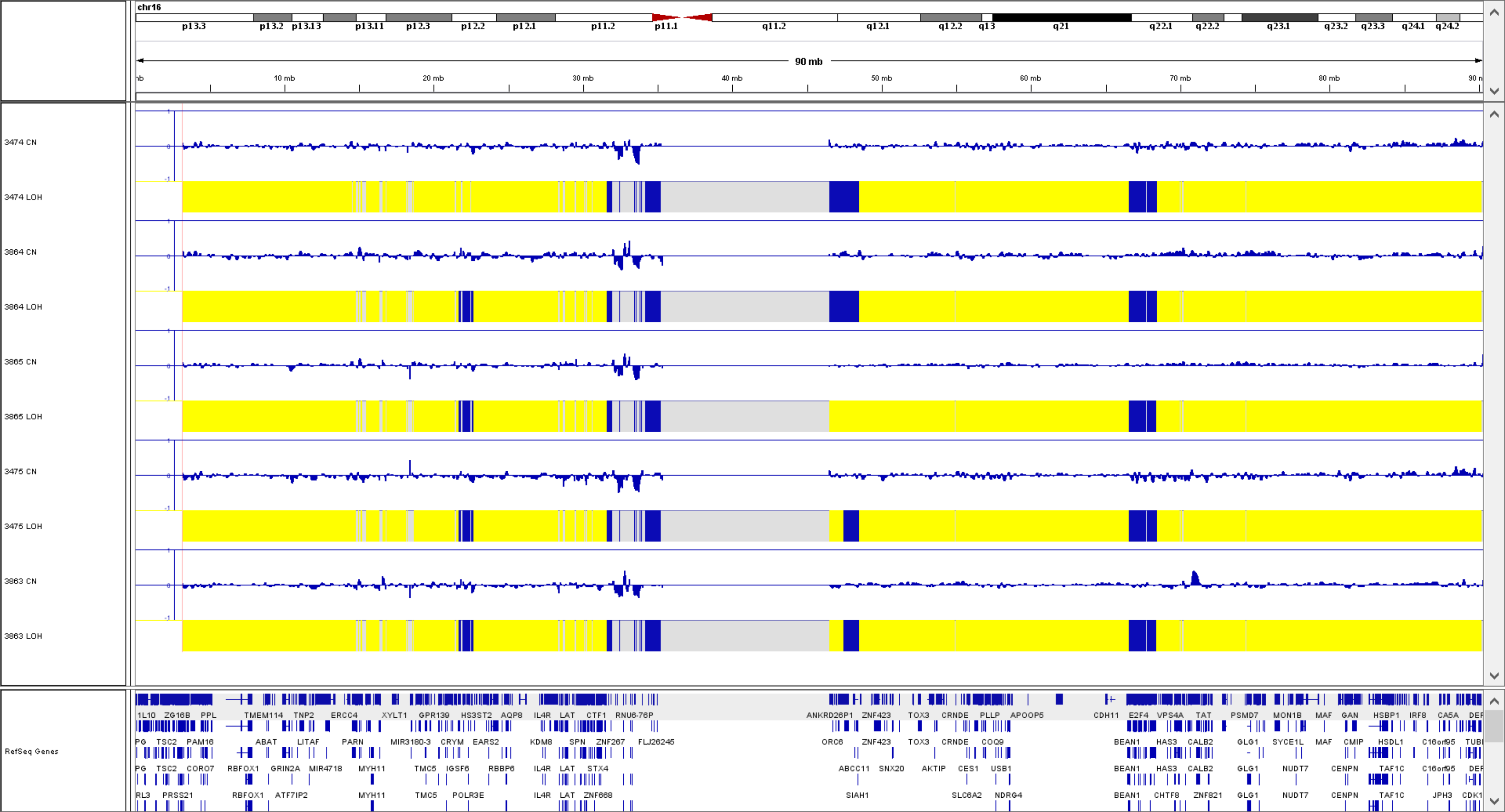
chr16
p13.3 p13.2 p13.13 p13.11 p12.3 p12.2 p12.1 p11.2 p11.1 q11.2 q12.1 q12.2 q13 q21 q22.1 q22.2 q23.1 q23.2 q23.3 q24.1 q24.2
90 mb
10 mb 20 mb 30 mb 40 mb 50 mb 60 mb 70 mb 80 mb
3474 CN
3474 LOH
3864 CN
3864 LOH
3865 CN
3865 LOH
3475 CN
3475 LOH
3863 CN
3863 LOH
RefSeq Genes
ZG16B PPL TMEM114 TNP2 ERCC4 XYLT1 GPR139 HS3ST2 AQP8 IL4R LAT CTF1 RNU6-76P ANKRD26P1 ZNF423 TOX3 CRNDE PLLP APOOP5 CDH11 E2F4 VPS4A TAT PSMD7 MON1B MAF GAN HSBP1 IRF8 CA5A
PG TSC2 PAM16 ABAT LITAF PARN MIR3180-3 CRYM EARS2 KDM8 SPN ZNF267 FLJ26245 ORC6 ZNF423 TOX3 CRNDE COQ9 BEAN1 HAS3 CALB2 GLG1 SYCE1L MAF CMIP HSDL1 C16orf95
PG TSC2 CORO7 RBFOX1 GRIN2A MIR4718 MYH11 TMC5 IGSF6 RBBP6 IL4R LAT STX4 ABCC11 SNX20 AKTIP CES1 USB1 BEAN1 HAS3 CALB2 GLG1 NUDT7 CENPN TAF1C C16orf95
RL3 PRSS21 RBFOX1 ATF7IP2 MYH11 TMC5 POLR3E IL4R LAT ZNF668 SIAH1 SLC6A2 NDRG4 BEAN1 CHTF8 ZNF821 GLG1 NUDT7 CENPN TAF1C JPH3 CDK1

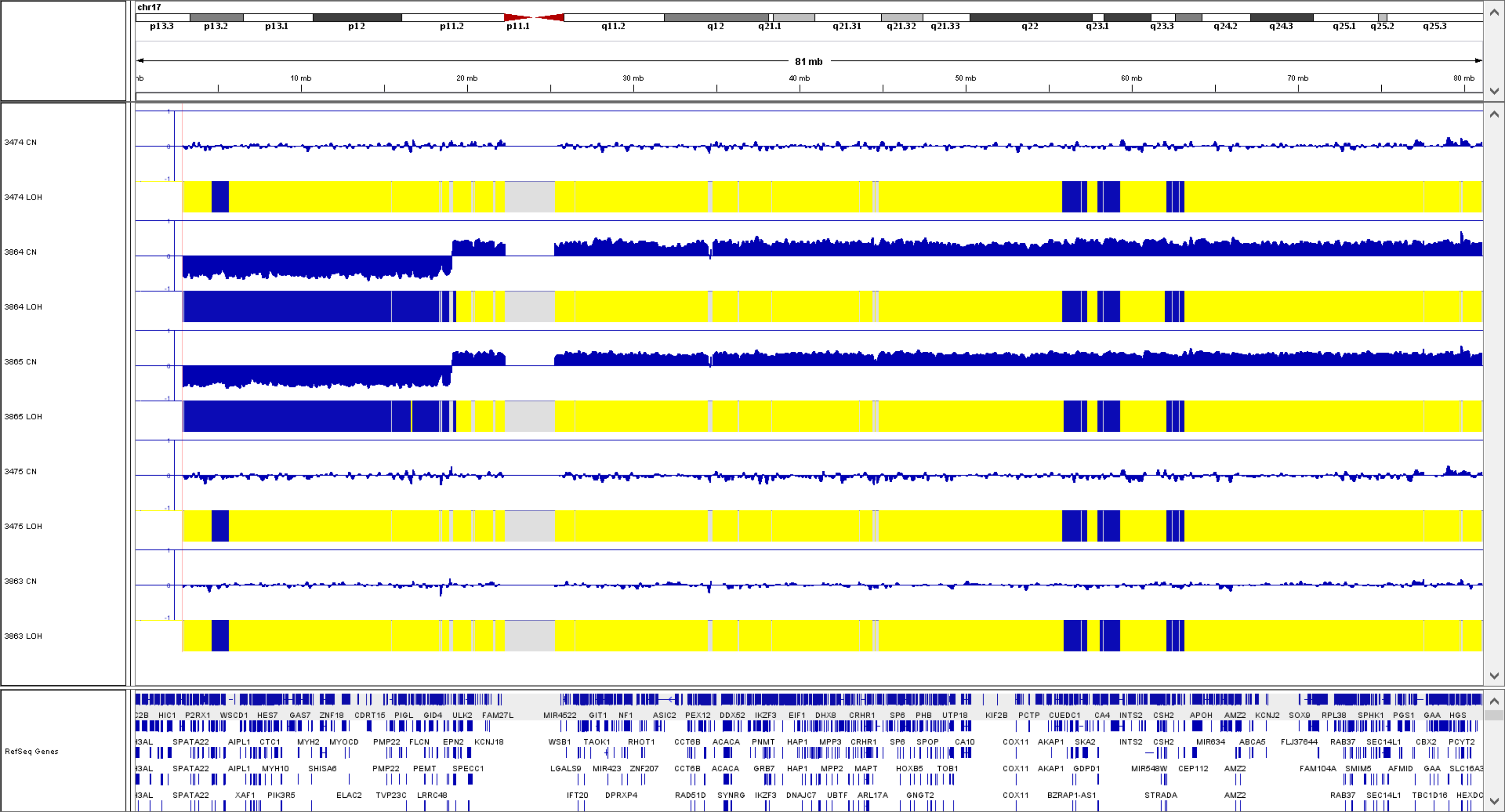
chr17
p13.3 p13.2 p13.1 p12 p11.2 p11.1 q11.2 q12 q21.1 q21.31 q21.32 q21.33 q22 q23.1 q23.3 q24.2 q24.3 q25.1 q25.2 q25.3
81 mb
10 mb 20 mb 30 mb 40 mb 50 mb 60 mb 70 mb 80 mb
3474 CN
3474 LOH
3864 CN
3864 LOH
3865 CN
3865 LOH
3475 CN
3475 LOH
3863 CN
3863 LOH
RefSeq Genes
HIC1 P2RX1 WSCD1 HES7 GAS7 ZNF18 CDRT15 PIGL GID4 ULK2 FAM27L MIR4522 GIT1 NF1 ASIC2 PEX12 DDX52 IKZF3 EIF1 DHX8 CRHR1 SP6 PHB UTP18 KIF2B PCTP CUEDC1 CA4 INTS2 CSH2 APOH AMZ2 KCNJ2 SOX9 RPL38 SPHK1 PGS1 GAA HGS

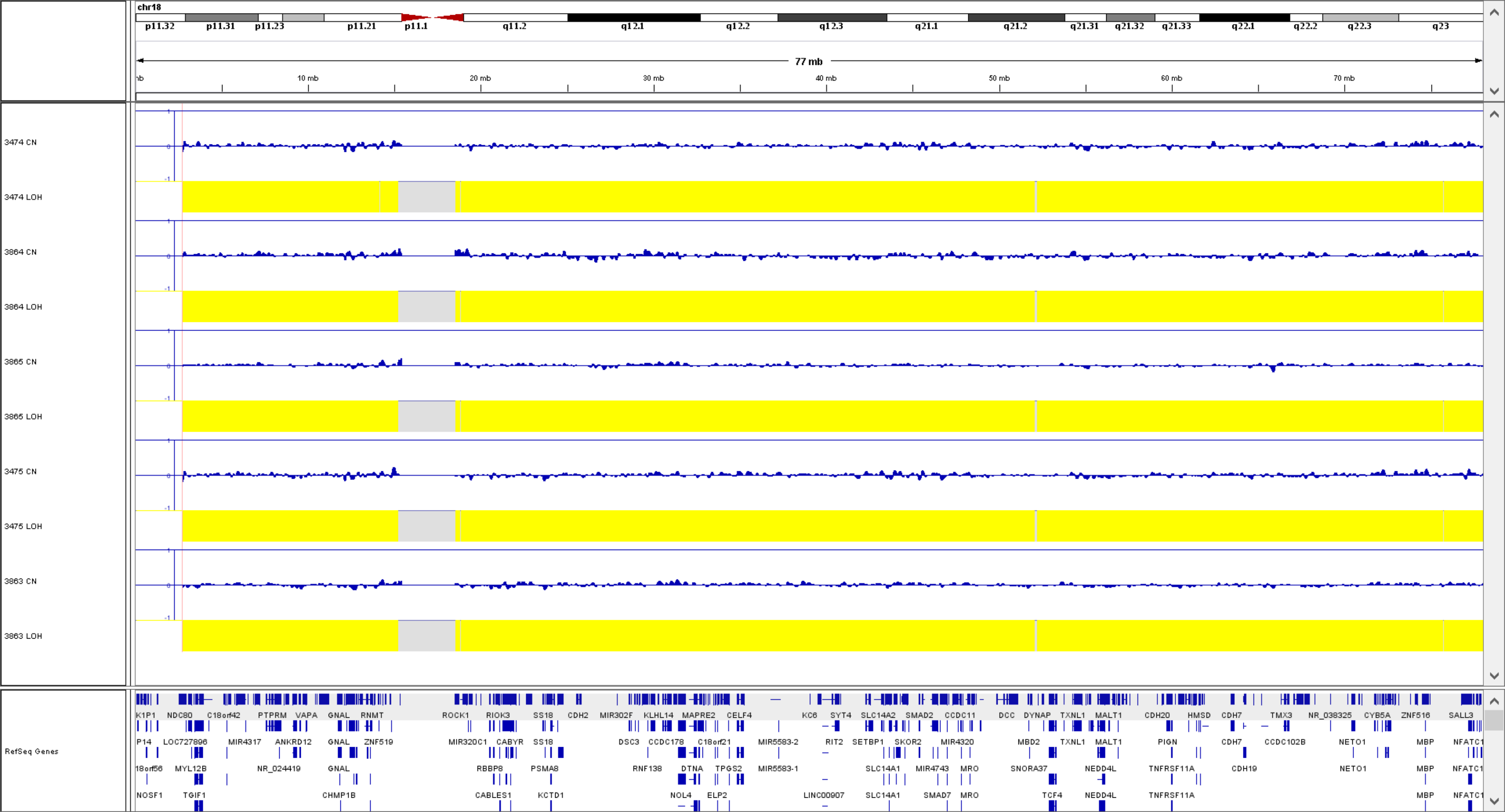

chr18
p11.32
p11.31
p11.23
p11.21
p11.1
q11.2
q12.1
q12.2
q12.3
q21.1
q21.2
q21.31
q21.32
q21.33
q22.1
q22.2
q22.3
q23
77 mb
10 mb
20 mb
30 mb
40 mb
50 mb
60 mb
70 mb
3474 CN
3474 LOH
3864 CN
3864 LOH
3865 CN
3865 LOH
3475 CN
3475 LOH
3863 CN
3863 LOH
RefSeq Genes
NDC80
C18orf42
PTPRM
VAPA
GNAL
RNMT
ROCK1
RIOK3
SS18
CDH2
MIR302F
KLHL14
MAPRE2
CELF4
SYT4
SLC14A2
SMAD2
CCDC11
DCC
DYNAP
TXNL1
MALT1
CDH20
HMSD
CDH7
TMX3
NR_038325
CYB5A
ZNF516
SALL3
LOC727896
MIR4317
ANKRD12
ZNF519
MIR320C1
CABYR
DSC3
CCDC178
C18orf21
MIR5583-2
RIT2
SETBP1
SKOR2
MIR4320
MBD2
PIGN
CCDC102B
NETO1
MBP
MYL12B
NR_024419
RBBP8
PSMA8
RNF138
DTNA
TPGS2
MIR5583-1
SLC14A1
MIR4743
MRO
SNORA37
NEDD4L
TNFRSF11A
CDH19
TGIF1
CHMP1B
CABLES1
KCTD1
NOL4
ELP2
LINC00907
SMAD7
TCF4

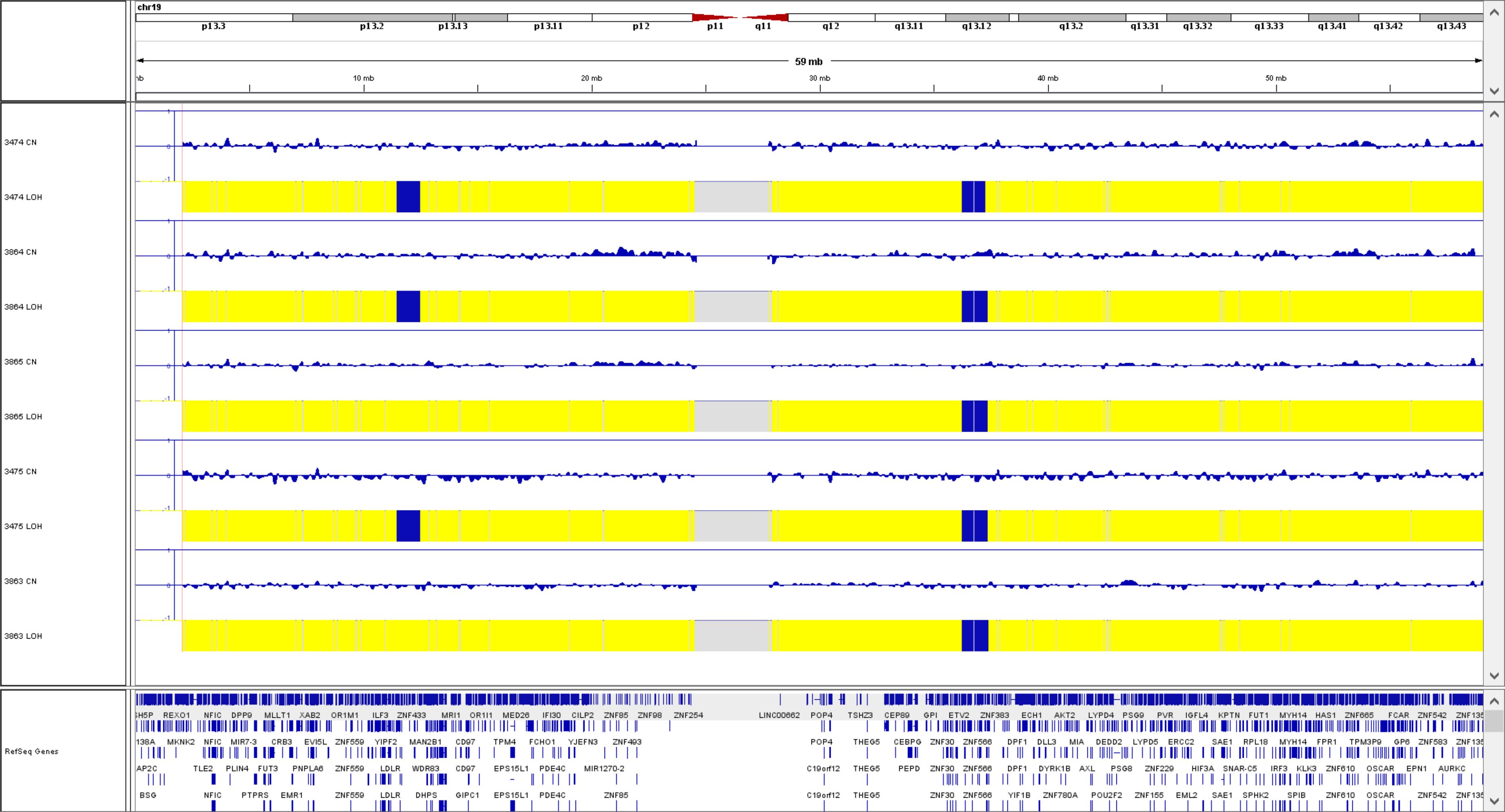
chr19
p13.3 p13.2 p13.13 p13.11 p12 p11 q11 q12 q13.11 q13.12 q13.2 q13.31 q13.32 q13.33 q13.41 q13.42 q13.43
59 mb
10 mb 20 mb 30 mb 40 mb 50 mb
3474 CN
3474 LOH
3864 CN
3864 LOH
3865 CN
3865 LOH
3475 CN
3475 LOH
3863 CN
3863 LOH
RefSeq Genes
REXO1 NFIC DPP9 MLLT1 XAB2 OR1M1 ILF3 ZNF433 MRI1 OR1I1 MED26 IFI30 CILP2 ZNF85 ZNF98 ZNF254 LINC00662 POP4 TSHZ3 CEP89 GPI ETV2 ZNF383 ECH1 AKT2 LYPD4 PSG9 PVR IGFL4 KPTN FUT1 MYH14 HAS1 ZNF665 FCAR ZNF542
MKNK2 NFIC MIR7-3 CRB3 EVI5L ZNF559 YIPF2 MAN2B1 CD97 TPM4 FCHO1 YJEFN3 ZNF493 POP4 THEG5 CEBPG ZNF30 ZNF566 DPF1 DLL3 MIA DEDD2 LYPD5 ERCC2 SAE1 RPL18 MYH14 FPR1 TPM3P9 GP6 ZNF583
TLE2 PLIN4 FUT3 PNPLA6 ZNF559 LDLR WDR83 CD97 EPS15L1 PDE4C MIR1270-2 C19orf12 THEG5 PEPD ZNF30 ZNF566 DPF1 DYRK1B AXL PSG8 ZNF229 HIF3A SNAR-C5 IRF3 KLK3 ZNF610 OSCAR EPN1
BSG NFIC PTPRS EMR1 ZNF559 LDLR DHPS GIPC1 EPS15L1 PDE4C ZNF85 C19orf12 THEG5 ZNF30 ZNF566 YIF1B ZNF780A POU2F2 ZNF155 EML2 SAE1 SPHK2 SPIB ZNF610 OSCAR ZNF542

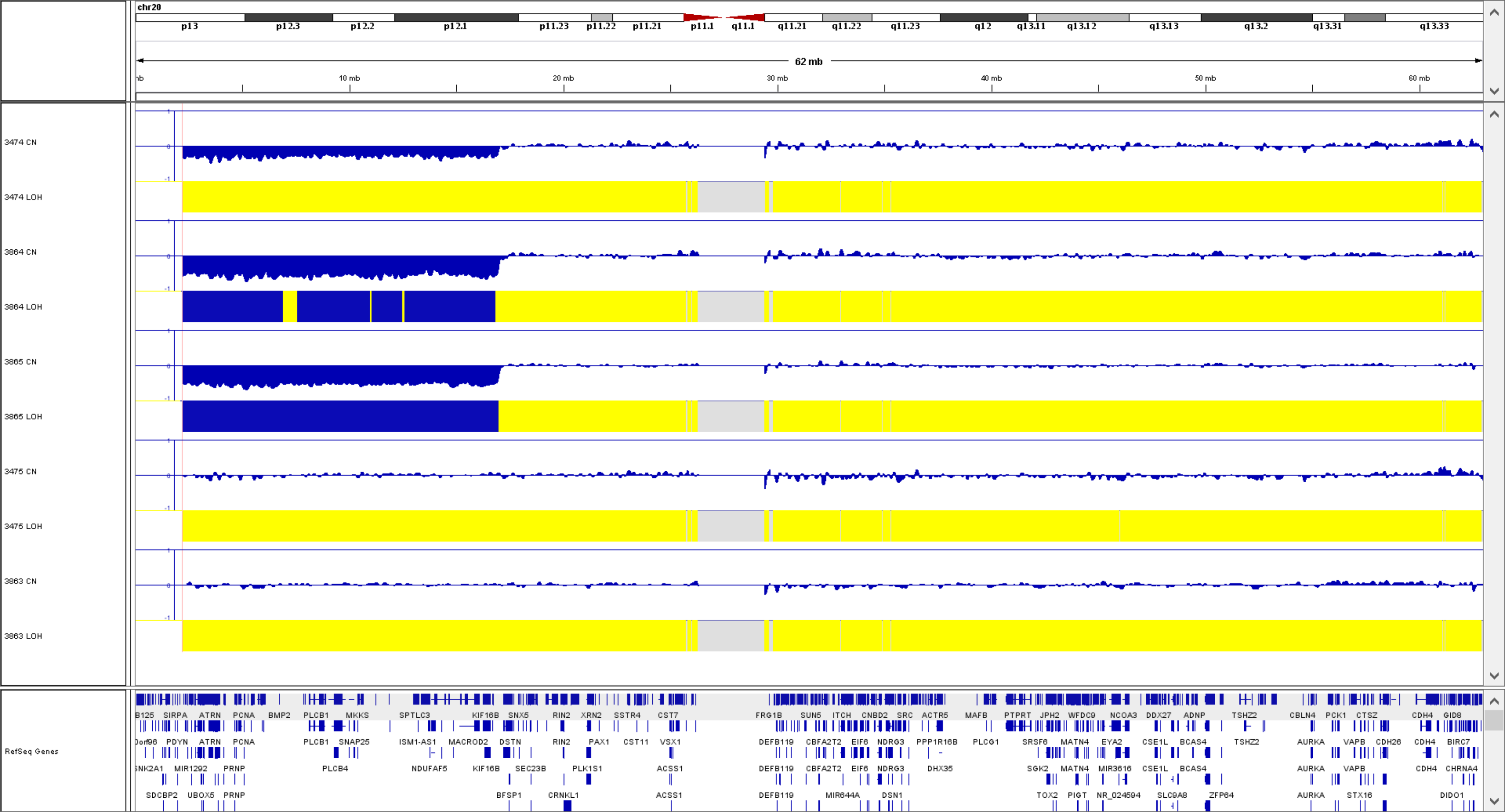
chr20
p13
p12.3
p12.2
p12.1
p11.23
p11.22
p11.21
p11.1
q11.1
q11.21
q11.22
q11.23
q12
q13.11
q13.12
q13.13
q13.2
q13.31
q13.33
62 mb
10 mb
20 mb
30 mb
40 mb
50 mb
60 mb
3474 CN
3474 LOH
3864 CN
3864 LOH
3865 CN
3865 LOH
3475 CN
3475 LOH
3863 CN
3863 LOH
RefSeq Genes

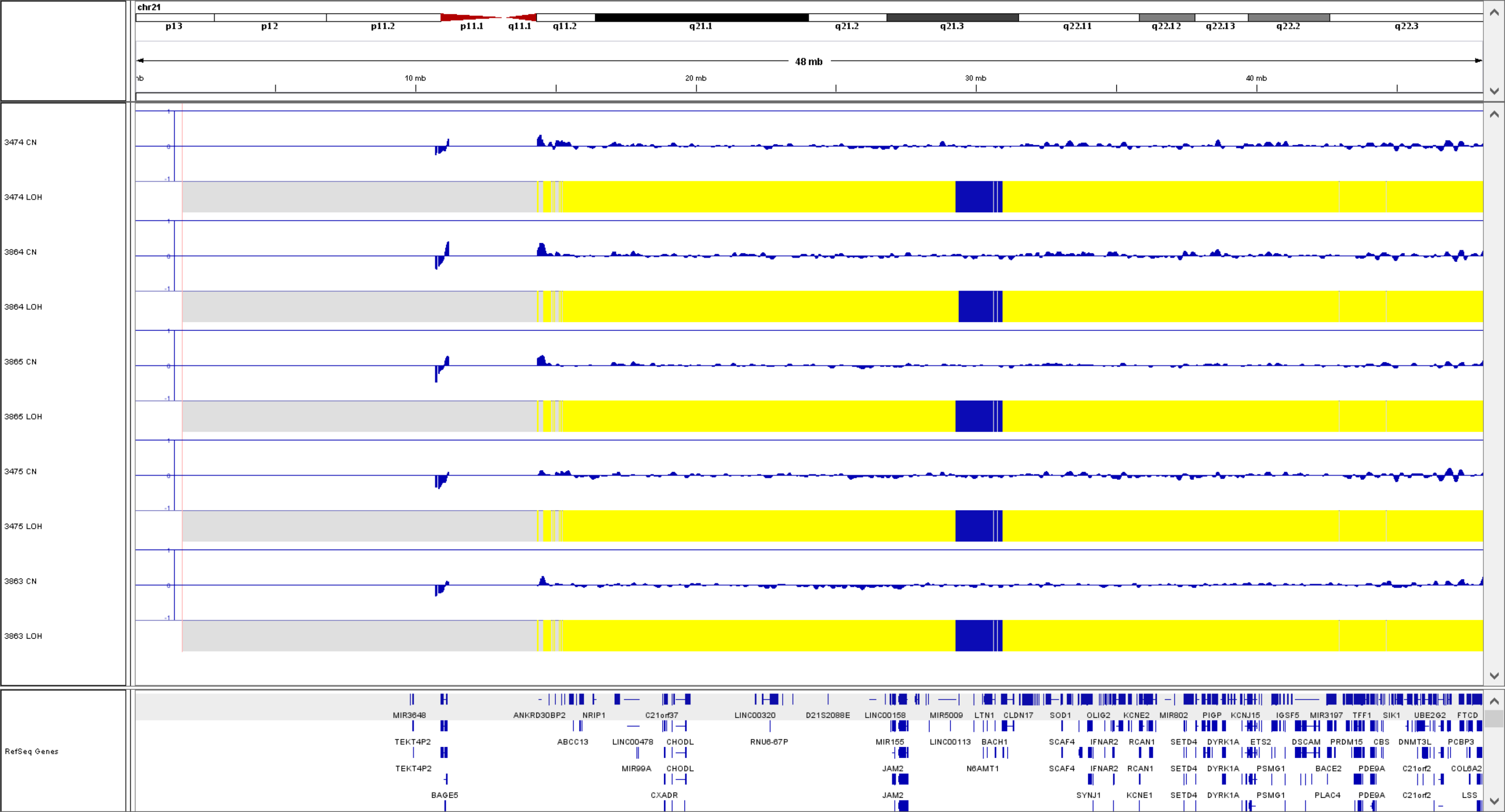
chr21
p13
p12
p11.2
p11.1
q11.1
q11.2
q21.1
q21.2
q21.3
q22.11
q22.12
q22.13
q22.2
q22.3
48 mb
10 mb
20 mb
30 mb
40 mb
3474 CN
3474 LOH
3864 CN
3864 LOH
3865 CN
3865 LOH
3475 CN
3475 LOH
3863 CN
3863 LOH
RefSeq Genes
MIR3648
ANKRD30BP2
NRIP1
C21orf37
LINC00320
D21S2088E
LINC00158
MIR5009
LTN1
CLDN17
SOD1
OLIG2
KCNE2
MIR802
PIGP
KCNJ15
IGSF5
MIR3197
TFF1
SIK1
UBE2G2
FTCD
TEKT4P2
ABCC13
LINC00478
CHODL
RNU6-67P
MIR155
LINC00113
BACH1
SCAF4
IFNAR2
RCAN1
SETD4
DYRK1A
ETS2
DSCAM
PRDM15
CBS
DNMT3L
PCBP3
MIR99A
JAM2
N6AMT1
PSMG1
BACE2
PDE9A
C21orf2
COL6A2
BAGE5
CXADR
SYNJ1
KCNE1
PLAC4
LSS

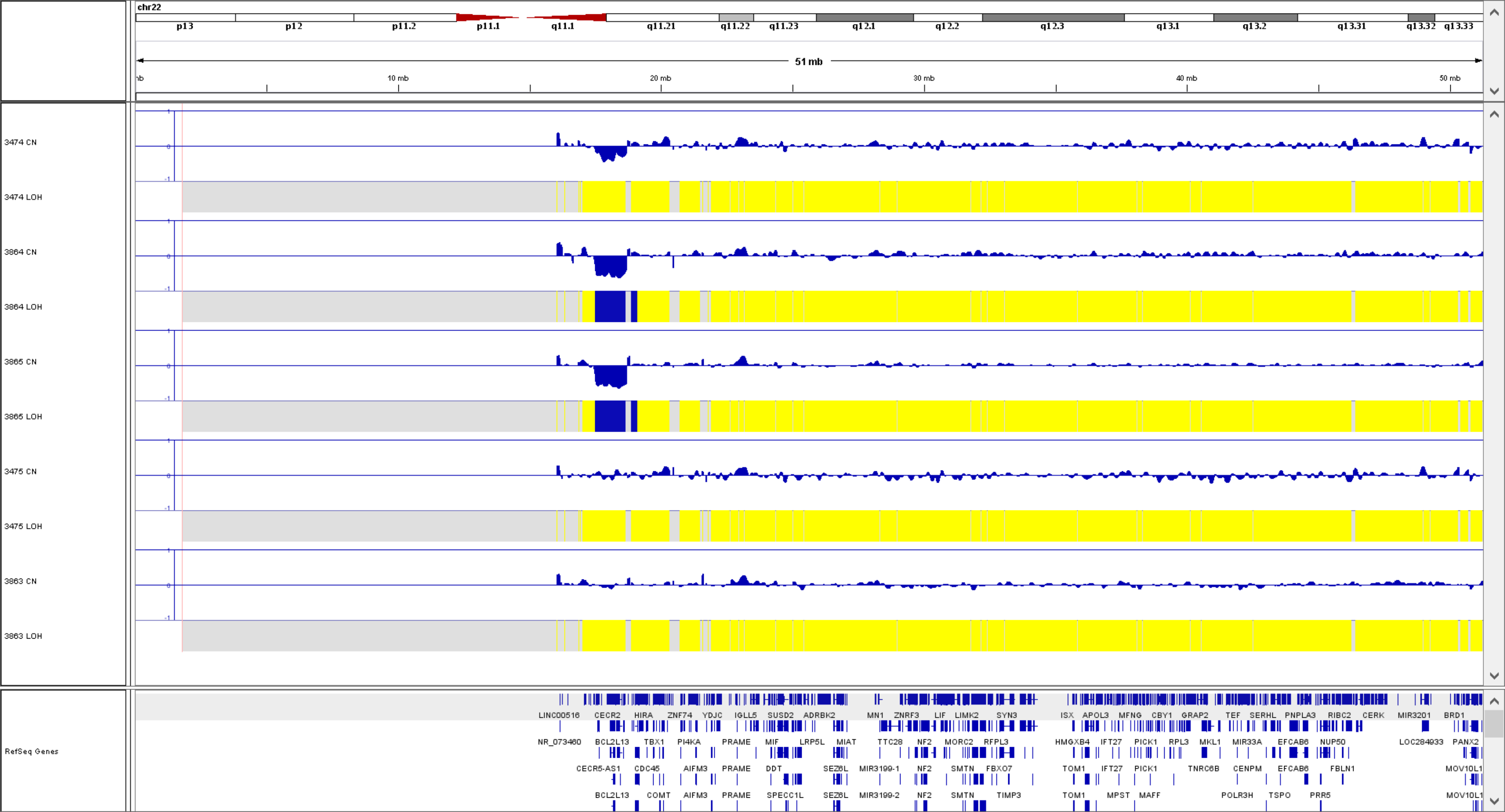
chr22
p13
p12
p11.2
p11.1
q11.1
q11.21
q11.22
q11.23
q12.1
q12.2
q12.3
q13.1
q13.2
q13.31
q13.32
q13.33
51 mb
10 mb
20 mb
30 mb
40 mb
50 mb
3474 CN
3474 LOH
3864 CN
3864 LOH
3865 CN
3865 LOH
3475 CN
3475 LOH
3863 CN
3863 LOH
RefSeq Genes
LINC00516
CECR2
HIRA
ZNF74
YDJC
IGLL5
SUSD2
ADRBK2
MN1
ZNRF3
LIF
LIMK2
SYN3
ISX
APOL3
MFNG
CBY1
GRAP2
TEF
SERHL
PNPLA3
RIBC2
CERK
MIR3201
BRD1
NR_073460
BCL2L13
TBX1
PI4KA
PRAME
MIF
LRP5L
MIAT
TTC28
NF2
MORC2
RFPL3
HMGXB4
IFT27
PICK1
RPL3
MKL1
MIR33A
EFCAB6
NUP50
LOC284933
PANX2
CECR5-AS1
CDC45
AIFM3
PRAME
DDT
SEZ6L
MIR3199-1
NF2
SMTN
FBXO7
TOM1
IFT27
PICK1
TNRC6B
CENPM
EFCAB6
FBLN1
MOV10L1
BCL2L13
COMT
AIFM3
PRAME
SPECC1L
SEZ6L
MIR3199-2
NF2
SMTN
TIMP3
TOM1
MPST
MAFF
POLR3H
TSPO
PRR5
MOV10L1

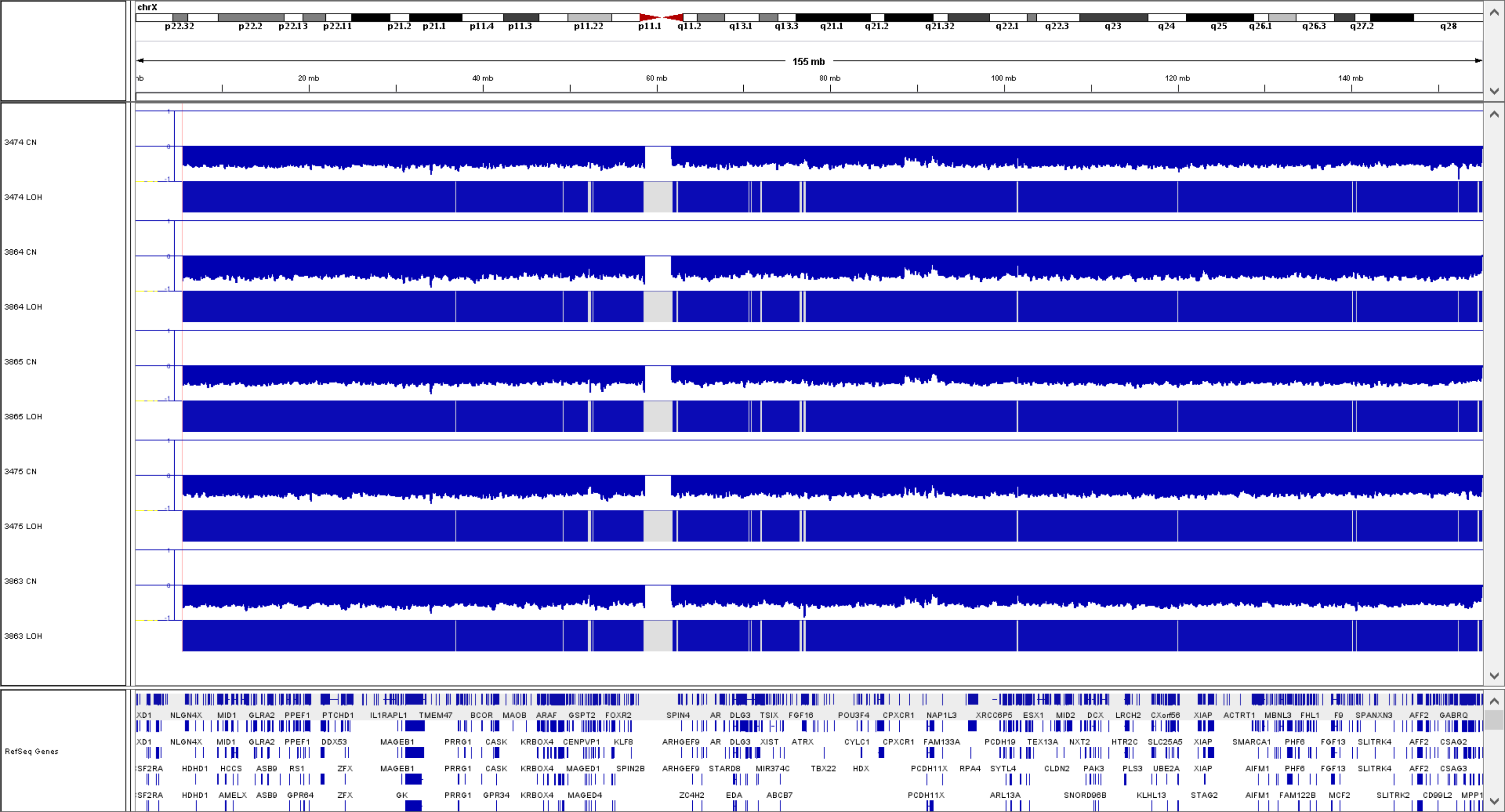
chrX
p22.32 p22.2 p22.13 p22.11 p21.2 p21.1 p11.4 p11.3 p11.22 p11.1 q11.2 q13.1 q13.3 q21.1 q21.2 q21.32 q22.1 q22.3 q23 q24 q25 q26.1 q26.3 q27.2 q28
155 mb
20 mb
40 mb
60 mb
80 mb
100 mb
120 mb
140 mb
3474 CN
3474 LOH
3864 CN
3864 LOH
3865 CN
3865 LOH
3475 CN
3475 LOH
3863 CN
3863 LOH
RefSeq Genes
NLGN4X MID1 GLRA2 PPEF1 PTCHD1 IL1RAPL1 TMEM47 BCOR MAOB ARAF GSPT2 FOXR2 SPIN4 AR DLG3 TSIX FGF16 POU3F4 CPXCR1 NAP1L3 XRCC6P5 ESX1 MID2 DCX LRCH2 CXorf56 XIAP ACTRT1 MBNL3 FHL1 F9 SPANXN3 AFF2 GABRQ
NLGN4X MID1 GLRA2 PPEF1 DDX53 MAGEB1 PRRG1 CASK KRBOX4 CENPVP1 KLF8 ARHGEF9 AR DLG3 XIST ATRX CYLC1 CPXCR1 FAM133A PCDH19 TEX13A NXT2 HTR2C SLC25A5 XIAP SMARCA1 PHF6 FGF13 SLITRK4 AFF2 CSAG2
HDHD1 HCCS ASB9 RS1 ZFX MAGEB1 PRRG1 CASK KRBOX4 MAGED1 SPIN2B ARHGEF9 STARD8 MIR374C TBX22 HDX PCDH11X RPA4 SYTL4 CLDN2 PAK3 PLS3 UBE2A XIAP AIFM1 PHF6 FGF13 SLITRK4 AFF2 CSAG3
HDHD1 AMELX ASB9 GPR64 ZFX GK PRRG1 GPR34 KRBOX4 MAGED4 ZC4H2 EDA ABCB7 PCDH11X ARL13A SNORD96B KLHL13 STAG2 AIFM1 FAM122B MCF2 SLITRK2 CD99L2

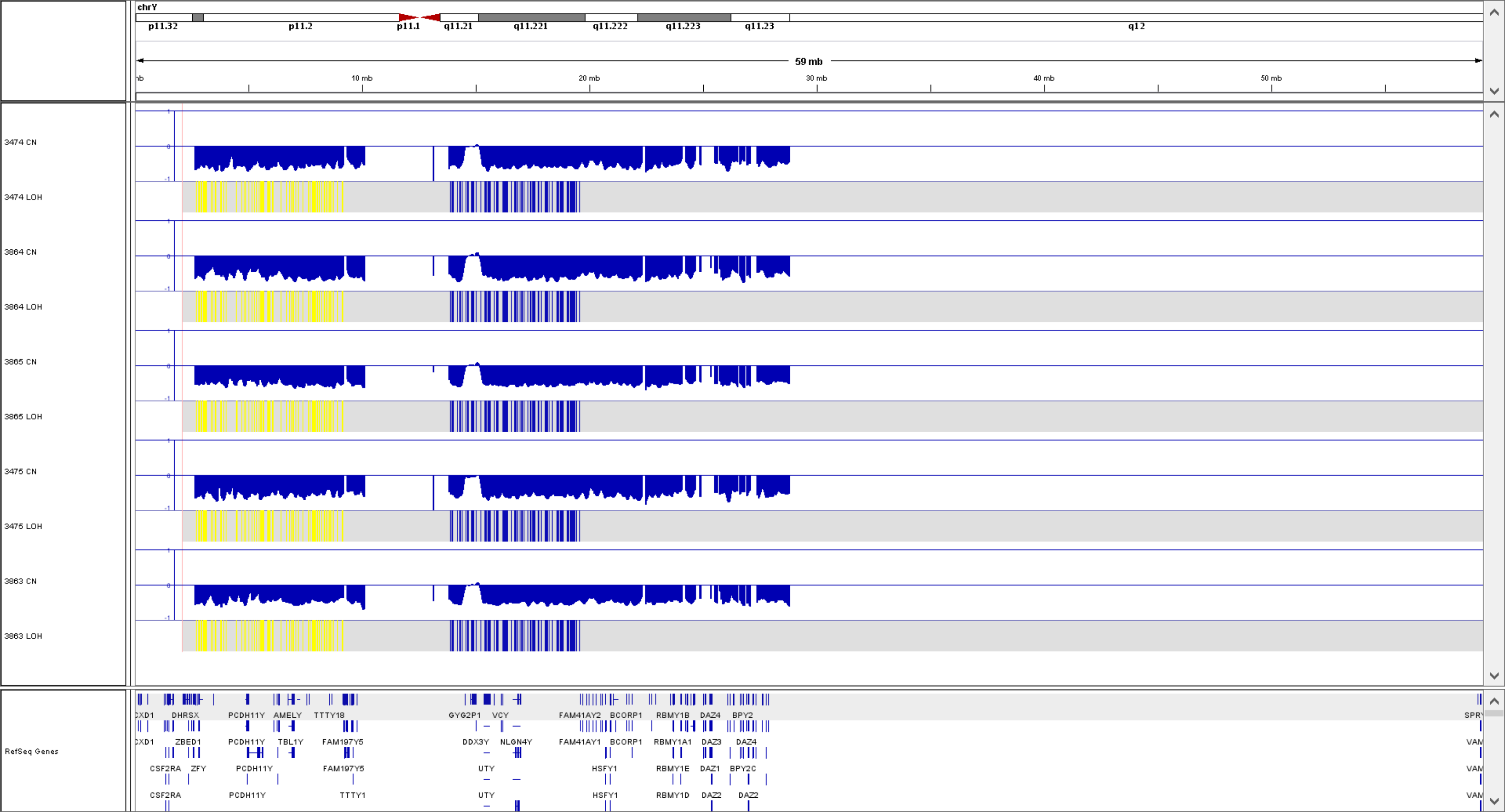
chrY
p11.32
p11.2
p11.1
q11.21
q11.221
q11.222
q11.223
q11.23
q12
59 mb
10 mb
20 mb
30 mb
40 mb
50 mb
3474 CN
3474 LOH
3864 CN
3864 LOH
3865 CN
3865 LOH
3475 CN
3475 LOH
3863 CN
3863 LOH
RefSeq Genes
DHRSX
PCDH11Y
AMELY
TTTY18
GYG2P1
VCY
FAM41AY2
BCORP1
RBMY1B
DAZ4
BPY2
ZBED1
TBL1Y
FAM197Y5
DDX3Y
NLGN4Y
FAM41AY1
RBMY1A1
DAZ3
CSF2RA
ZFY
UTY
HSFY1
RBMY1E
DAZ1
BPY2C
TTTY1
RBMY1D
DAZ2
